# Supplementary material for: Study of Novel Furocoumarin Derivatives on Anti-Vitiligo Activity, Molecular Docking and Mechanism of Action
Source: Int J Mol Sci. 2022 Jul 19;23(14):7959. doi: 10.3390/ijms23147959 (PMC9316487; doi:10.3390/ijms23147959)

Synthesis and anti-vitiligo activity of novel furocoumarin derivatives and their molecular docking study

Chao Niu[a,b,c], Deng Zang[a], Haji Akber Aisa[*a,b]

[a] *State Key Laboratory Basis of Xinjiang Indigenous Medicinal Plants Resource Utilization and Key Laboratory of Plant Resources and Chemistry of Arid Zone, Xinjiang Technical Institute of Physics and Chemistry, Chinese Academy of Sciences, Urumqi 830011, China;* [b] *University of Chinese Academy of Sciences, Beijing, 100049.* [c] *Nantong Chanyoo Pharmatech Co., Ltd., Nantong 226407, China.*

Correspondence e-mail: aisa@ms.xjb.ac.cn

NMR and other chemical characterizations of **7a-7ad**

(7-oxo-3-phenyl-7H-furo[3,2-g]chromen-5-yl)methyl-4-fluorobenzenesulfonate (**7a**)

Yield 84%, m.p. 170-171°C. ^{1}H NMR (400 MHz, $CDCl_3$) δ 7.97 – 7.92 (m, 2H), 7.87 (s, 1H), 7.85 (s, 1H), 7.56 (t, *J* = 7.5 Hz, 2H), 7.59 – 7.53 (m, 2H), 7.52 (s, 1H), 7.47 (t, *J* = 7.3 Hz, 1H), 7.23 – 7.16 (m, 2H), 6.42 (s, 1H), 5.35 (d, *J* = 1.1 Hz, 2H). ^{13}C NMR (101 MHz, $CDCl_3$) δ 167.52, 164.95, 160.06, 157.34, 151.95, 146.78, 143.43, 131.52 (d, *J* = 3.3 Hz), 130.99 (d, *J* = 9.7 Hz), 130.71, 129.52, 128.43, 127.75, 124.51, 122.44, 117.18, 116.95, 115.02, 113.33, 100.90, 66.59. HRMS (ESI) calcd for $C_{24}H_{16}FO_6S^+$ $[M+H]^+$ 451.0646, found 451.0650.

(7-oxo-3-phenyl-7H-furo[3,2-g]chromen-5-yl)methyl-4-methoxybenzenesulfonate (**7b**)

Yield 87%, m.p. 183-185°C. ^{1}H NMR (400 MHz, $CDCl_3$) δ 7.90 (s, 1H), 7.87 – 7.82 (m, 3H), 7.65 – 7.61 (m, 2H), 7.56 (t, *J* = 7.6 Hz, 2H), 7.52 (s, 1H), 7.47 (t, *J* = 7.3 Hz, 1H), 6.96 (d, J = 9.0 Hz, 2H), 6.40 (s, 1H), 5.30 (d, *J* = 1.1 Hz, 2H), 3.87 (s, 3H). ^{13}C NMR (101 MHz, $CDCl_3$) δ 160.16, 157.33, 151.96, 147.05, 145.90, 143.37, 132.48, 130.75, 130.23, 129.53, 128.42, 128.14, 127.76, 124.48, 115.16, 113.60, 113.44, 100.82, 66.44, 21.85. HRMS (ESI) calcd for

$C_{25}H_{19}O_7S^+$ [M+H]$^+$ 463.0846, found 463.0840.

(7-oxo-3-phenyl-7H-furo[3,2-g]chromen-5-yl)methyl -[1,1'-biphenyl]-4-sulfonate (**7c**)

Yield 81%, m.p. 171-173 °C. ^{1}H NMR (400 MHz, $CDCl_3$) δ 7.97 (d, J = 8.5 Hz, 2H), 7.91 (s, 1H), 7.85 (s, 1H), 7.69 (d, *J* = 8.4 Hz, 2H), 7.63 (d, *J* = 7.3 Hz, 2H), 7.59 – 7.53 (m, 4H), 7.52 – 7.43 (m, 5H), 6.46 (s, 1H), 5.37 (s, 2H). ^{13}C NMR (101 MHz, $CDCl_3$) δ 160.13, 157.33, 147.69, 146.93, 143.39, 138.88, 133.90, 130.75, 129.54, 129.30, 129.07, 128.62, 128.43, 128.17, 127.77, 127.53, 124.49, 122.49, 115.18, 113.82, 113.43, 100.86, 66.68. HRMS (ESI) calcd for $C_{30}H_{21}O_6S^+$ [M+H]$^+$ 509.1053, found 509.1061.

(7-oxo-3-phenyl-7H-furo[3,2-g]chromen-5-yl)methyl -4-methylbenzenesulfonate (**7d**)

Yield 80%, m.p. 181-182 °C. ^{1}H NMR (400 MHz, $CDCl_3$) δ 7.87 (s, 1H), 7.83 (s, 1H), 7.78 (d, *J* = 8.3 Hz, 2H), 7.63 – 7.59 (m, 2H), 7.54 (t, *J* = 7.6 Hz, 2H), 7.50 (s, 1H), 7.45 (t, *J* = 7.3 Hz, 1H), 7.29 (d, *J* = 8.1 Hz, 2H), 6.37 (s, 1H), 5.29 (d, *J* = 1.1 Hz, 2H), 2.42 (s, 3H). ^{13}C NMR (101 MHz, $CDCl_3$) δ 160.16, 157.33, 151.96, 147.05, 145.90, 143.37, 132.48, 130.75, 130.23, 129.53, 128.42, 128.14, 127.76, 124.48, 115.16, 113.60, 113.44, 100.82, 66.44, 21.85. HRMS (ESI) calcd for $C_{25}H_{19}O_6S^+$ [M+H]$^+$ 447.0897, found 447.0904.

(7-oxo-3-phenyl-7H-furo[3,2-g]chromen-5-yl)methyl-4-(trifluoromethoxy)benzenesulfonate (**7e**)

Yield 83%, m.p. 190-192 °C. ^{1}H NMR (400 MHz, $CDCl_3$) δ 7.97 (d, J = 8.9 Hz, 2H), 7.88 (s, 1H), 7.86 (s, 1H), 7.65 – 7.60 (m, 2H), 7.59 – 7.52 (m, 3H), 7.47 (t, *J* = 7.3 Hz, 1H), 7.33 (d, *J* = 8.2 Hz, 2H), 6.45 (s, 1H), 5.37 (d, *J* = 1.0 Hz, 2H). ^{13}C NMR (101 MHz, $CDCl_3$) δ 160.03, 157.38, 153.59, 151.98, 146.63, 143.47, 133.70, 130.73, 130.38, 129.54, 128.46, 127.78, 124.56, 122.45, 121.23, 115.02, 113.84, 113.30, 100.96, 66.79. HRMS (ESI) calcd for $C_{25}H_{16}F_3O_7S^+$ [M+H]$^+$ 517.0563, found 517.0556.

(7-oxo-3-phenyl-7H-furo[3,2-g]chromen-5-yl)methyl -4-bromobenzenesulfonate (**7f**)

Yield 82%, m.p. 182-183 °C. ^{1}H NMR (400 MHz, $CDCl_3$) δ 7.84 (d, *J* = 4.4 Hz, 2H), 7.75 (d, *J* = 8.8 Hz, 2H), 7.66 – 7.58 (m, 4H), 7.57 – 7.51 (m, 3H), 7.47 (d, *J* = 7.0 Hz, 1H), 6.42 (s, 1H), 5.33 (d, *J* = 1.0 Hz, 2H). ^{13}C NMR (101 MHz, $CDCl_3$) δ 160.46, 157.32, 154.01, 146.67, 145.38, 143.46 137.25, 134.50, 132.99, 129.54, 129.49, 128.47, 127.79, 124.33, 122.23, 115.05, 113.85, 100.96, 66.56. HRMS (ESI) calcd for $C_{24}H_{16}BrO_6S^+$ [M+H]$^+$ 510.9845, found 510.9837.

(7-oxo-3-phenyl-7H-furo[3,2-g]chromen-5-yl)methyl 4-chlorobenzenesulfonate (**7g**)

Yield 88%, m.p. 204-206 °C. ^1H NMR (400 MHz, $CDCl_3$) δ 7.88 – 7.83 (m, 4H), 7.64 – 7.60 (m, 2H), 7.59 – 7.52 (m, 3H), 7.49 (d, *J* = 8.7 Hz, 3H), 6.44 (s, 1H), 5.35 (s, 2H). ^{13}C NMR (101 MHz, $CDCl_3$) δ 160.06, 157.37, 151.98, 146.69, 143.45, 141.51, 133.96, 130.73, 130.00, 129.54, 129.48, 128.46, 127.78, 124.55, 122.46, 115.04, 113.81, 113.32, 100.94, 66.76. HRMS (ESI) calcd for $C_{24}H_{16}ClO_6S^+$ [M+H]$^+$ 467.0351, found 467.0360.

(7-oxo-3-phenyl-7H-furo[3,2-g]chromen-5-yl)methyl benzenesulfonate (**7h**)

Yield 81%, m.p. 155-156 °C. ^1H NMR (400 MHz, $CDCl_3$) δ 7.93 (d, *J* = 7.3 Hz, 2H), 7.89 (s, 1H), 7.85 (s, 1H), 7.69 – 7.60 (m, 3H), 7.59 – 7.50 (m, 5H), 7.47 (t, *J* = 7.3 Hz, 1H), 6.40 (s, 1H), 5.34 (d, J = 1.0 Hz, 2H). ^{13}C NMR (101 MHz, $CDCl_3$) δ 160.13, 157.34, 151.97, 146.91, 143.39, 135.55, 134.64, 130.74, 129.64, 129.53, 128.42, 128.07, 127.76, 124.50, 122.49, 115.10, 113.65, 113.40, 100.88, 66.58. HRMS (ESI) calcd for $C_{24}H_{17}O_6S^+$ [M+H]$^+$ 433.0740, found 433.0733.

(7-oxo-3-phenyl-7H-furo[3,2-g]chromen-5-yl)methyl-4-(trifluoromethyl)benzenesulfonate (**7i**)

Yield 80%, 213-215 °C. ^1H NMR (400 MHz, $CDCl_3$) δ 8.05 (d, *J* = 8.2 Hz, 2H), 7.86 (d, *J* = 4.4 Hz, 2H), 7.79 (s, 1H), 7.77 (s, 1H), 7.65 – 7.60 (m, 2H), 7.56 (t, *J* = 7.5 Hz, 2H), 7.53 (s, 1H), 7.48 (t, *J* = 7.3 Hz, 1H), 6.45 (s, 1H), 5.39 (s, 2H). ^{13}C NMR (101 MHz, $CDCl_3$) δ 159.98, 157.39, 151.97, 146.43, 143.50, 139.17, 130.72, 129.54, 128.65, 128.48, 127.78, 126.78 (q, J = 3.7 Hz), 124.57, 122.44, 114.98, 113.95, 113.24, 100.99, 67.08. HRMS (ESI) calcd for $C_{25}H_{16}F_3O_6S^+$ [M+H]$^+$ 501.0614, found 501.0622.

(7-oxo-3-phenyl-7H-furo[3,2-g]chromen-5-yl)methyl-3-chlorobenzenesulfonate (**7j**)

Yield 90%, m.p. 145-147 °C. ^1H NMR (400 MHz, $CDCl_3$) δ 7.91 – 7.87 (m, 2H), 7.86 (s, 1H), 7.81 (ddd, *J* = 7.9, 1.6, 1.0 Hz, 1H), 7.65 – 7.60 (m, 3H), 7.59 – 7.52 (m, 3H), 7.53 (s, 1H), 7.47 (t, *J* = 7.9 Hz, 2H), 6.44 (s, 1H), 5.37 (d, *J* = 1.0 Hz, 2H). ^{13}C NMR (101 MHz, $CDCl_3$) δ 160.04, 157.39, 151.98, 146.58, 143.45, 137.26, 135.93, 134.77, 130.90.130.71, 129.54, 128.45, 128.15, 127.77, 126.10, 124.57, 122.49, 115.07, 113.89, 113.28, 100.94, 67.04. HRMS (ESI) calcd for $C_{24}H_{16}ClO_6S^+$ [M+H]$^+$ 467.0351, found 467.0355.

(7-oxo-3-phenyl-7H-furo[3,2-g]chromen-5-yl)methyl -3-bromobenzenesulfonate (**7k**)

Yield 79%, 181-182 °C. ^1H NMR (400 MHz, $CDCl_3$) δ 8.04 (t, *J* = 1.8 Hz, 1H), 7.89 (s, 1H), 7.85 (s, 1H), 7.85 – 7.83 (m, 1H), 7.77 (ddd, *J* = 8.0, 1.7, 0.9 Hz, 1H), 7.65 – 7.60 (m, 2H), 7.59 – 7.51 (m, 3H), 7.47 (t, *J* = 7.3 Hz, 1H), 7.40 (t, *J* = 8.0 Hz, 1H), 6.43 (s, 1H), 5.36 (d, *J* =

1.0 Hz, 2H), 3.49 (s, 1H). ^{13}C NMR (101 MHz, $CDCl_3$) δ 160.02, 157.38, 151.98, 146.54, 143.44, 137.66, 137.40, 131.05, 130.95, 130.71, 129.54, 128.45, 127.76, 126.53, 124.57, 123.53, 122.49, 115.09, 113.95, 113.27, 100.94, 67.10. HRMS (ESI) calcd for $C_{24}H_{16}BrO_6S^+$ [M+H]$^+$ 510.9845, found 510.9837.

(7-oxo-3-phenyl-7H-furo[3,2-g]chromen-5-yl)methyl -2-chlorobenzenesulfonate (**7l**)

Yield 86%, m.p. 185-187 °C. ^{1}H NMR (400 MHz, $CDCl_3$) δ 8.11 (dd, *J* = 7.9, 1.6 Hz, 1H), 7.92 (s, 1H), 7.85 (s, 1H), 7.65 – 7.60 (m, 2H), 7.59 – 7.52 (m, 3H), 7.51 (s, 1H), 7.49 – 7.41 (m, 3H), 6.50 (s, 1H), 5.44 (d, *J* = 1.1 Hz, 2H). ^{13}C NMR (101 MHz, $CDCl_3$) δ 160.12, 157.36, 151.96, 146.55, 143.39, 135.44, 133.26, 132.32, 131.90, 130.73, 129.52, 128.40, 127.78, 127.38, 124.50, 122.53, 115.27, 114.03, 113.36, 100.82, 67.35. HRMS (ESI) calcd for $C_{24}H_{16}ClO_6S^+$ [M+H]$^+$ 467.0351, found 467.0344.

(7-oxo-3-phenyl-7H-furo[3,2-g]chromen-5-yl)methyl -2-fluorobenzenesulfonate (**7m**)

Yield 84%, m.p.159-160 °C. ^{1}H NMR (400 MHz, $CDCl_3$) δ 7.97 – 7.91 (m, 2H), 7.85 (s, 1H), 7.66 – 7.60 (m, 3H), 7.56 (t, *J* = 7.6 Hz, 2H), 7.52 (s, 1H), 7.46 (t, *J* = 7.4 Hz, 1H), 7.31 (td, *J* = 7.9, 0.8 Hz, 1H), 7.15 (t, *J* = 9.2 Hz, 1H), 6.48 (s, 1H), 5.48 (d, *J* = 0.8 Hz, 2H). ^{13}C NMR (101 MHz, $CDCl_3$) δ 160.11, 157.37, 151.97, 146.69, 143.40, 137.01 (d, J = 8.5 Hz), 130.91, 130.72, 129.53, 128.40, 127.78, 124.86 (d, J = 4.0 Hz), 124.52, 122.55, 117.69. 117.49, 115.19, 113.89, 100.85, 67.51.HRMS (ESI) calcd for $C_{24}H_{16}FO_6S^+$ [M+H]$^+$ 451.0646, found 451.0652.

(7-oxo-3-phenyl-7H-furo[3,2-g]chromen-5-yl)methyl -3-fluorobenzenesulfonate (**7n**)

Yield 82%, m.p. 157-159 °C. ^{1}H NMR (400 MHz, $CDCl_3$) δ 7.88 (s, 1H), 7.85 (s, 1H), 7.74 (d, *J* = 7.9 Hz, 1H), 7.66 – 7.59 (m, 3H), 7.59 – 7.50 (m, 4H), 7.47 (t, *J* = 7.3 Hz, 1H), 7.37 (td, *J* = 8.0, 0.8 Hz, 1H), 6.43 (s, 1H), 5.36 (d, *J* = 1.0 Hz, 2H). ^{13}C NMR (101 MHz, $CDCl_3$) δ 163.76, 161.24, 160.04, 157.37, 151.97, 146.63, 143.44, 137.41 (d, *J* = 7.2 Hz), 131.59 (d, *J* = 7.8 Hz), 130.70, 129.52, 128.44, 127.76, 124.55, 123.87 (d, *J* = 3.5 Hz), 122.48, 121.99 (d, *J* = 21.1 Hz), 115.67, 115.22 (d, *J* = 41.9 Hz), 113.51 (d, *J* = 43.9 Hz), 100.94, 66.91. HRMS (ESI) calcd for $C_{24}H_{16}FO_6S^+$ [M+H]$^+$ 451.0646, found 451.0641.

(7-oxo-3-phenyl-7H-furo[3,2-g]chromen-5-yl)methyl -2-bromobenzenesulfonate (**7o**)

Yield 87%, m.p. 188-190 °C. ^{1}H NMR (400 MHz, $CDCl_3$) δ 8.14 (dd, *J* = 7.2, 2.4 Hz, 1H), 7.93 (s, 1H), 7.85 (s, 1H), 7.70 (dd, *J* = 7.5, 1.7 Hz, 1H), 7.65 – 7.61 (m, 2H), 7.55 (t, *J* = 7.5 Hz, 2H), 7.51 (s, 1H), 7.50 – 7.43 (m, 3H), 6.51 (s, 1H), 5.43 (d, *J* = 1.0 Hz, 2H).^{13}C NMR (101 MHz, $CDCl_3$) δ 160.13, 157.36, 151.97, 146.51, 143.39, 135.89, 135.69, 135.35, 132.20, 130.75,

129.52, 128.40, 127.94, 127.80, 124.51, 122.53, 121.18, 115.32, 114.12, 113.38, 100.83, 67.29. HRMS (ESI) calcd for $C_{24}H_{16}BrO_6S^+$ [M+H]$^+$ 510.9845, found 510.9840.

(7-oxo-3-phenyl-7H-furo[3,2-g]chromen-5-yl)methyl- 3-methylbenzenesulfonate (**7p**)

Yield 82%, m.p. 176-177 °C. ^{1}H NMR (400 MHz, $CDCl_3$) δ 7.90 (s, 1H), 7.85 (s, 1H), 7.73 – 7.70 (m, 2H), 7.65 – 7.61 (m, 2H), 7.56 (t, J = 7.5 Hz, 2H), 7.51 (s, 1H), 7.49 – 7.42 (m, 2H), 7.39 (t, J = 7.9 Hz, 1H), 6.41 (s, 1H), 5.33 (d, J = 1.1 Hz, 2H), 2.38 (s, 3H). ^{13}C NMR (101 MHz, $CDCl_3$) δ 160.14, 157.33, 151.97, 146.95, 143.38, 140.09, 135.42, 130.74, 129.53, 129.42, 128.42, 128.38, 127.74, 125.21, 124.46, 122.49, 115.18, 113.72, 113.43, 100.83, 87.60, 66.57, 21.40. HRMS (ESI) calcd for $C_{25}H_{19}O_6S^+$ [M+H]$^+$ 447.0897, found 447.0886.

(7-oxo-3-phenyl-7H-furo[3,2-g]chromen-5-yl)methyl-4-isopropylbenzenesulfonate (**7q**)

Yield 76%, m.p. 185-187 °C. ^{1}H NMR (400 MHz, $CDCl_3$) δ 7.91 (s, 1H), 7.85 (s, 1H), 7.82 (d, J = 8.5 Hz, 2H), 7.65 – 7.61 (m, 2H), 7.56 (t, J = 7.6 Hz, 2H), 7.51 (s, 1H), 7.47 (t, J = 7.3 Hz, 1H), 7.35 (d, J = 8.3 Hz, 2H), 6.40 (s, 1H), 5.33 (d, J = 1.1 Hz, 2H), 3.49 (s, 1H), 1.26 (d, J = 6.9 Hz, 6H). ^{13}C NMR (101 MHz, $CDCl_3$) δ 160.15, 157.32, 156.49, 151.97, 147.03, 143.37, 132.76, 130.76, 129.54, 128.43, 128.28, 127.77, 127.72, 124.47, 122.51, 115.20, 113.67, 113.46, 100.83, 66.47, 34.47, 23.66. HRMS (ESI) calcd for $C_{27}H_{23}O_6S^+$ [M+H]$^+$ 475.1210, found 475.1200.

(7-oxo-3-phenyl-7H-furo[3,2-g]chromen-5-yl)methyl-4-(tert-butyl)benzenesulfonate (**7r**)

Yield 81%, m.p. 216-218 °C. ^{1}H NMR (400 MHz, $CDCl_3$) δ 7.91 (s, 1H), 7.85 (s, 1H), 7.82 (d, J = 8.7 Hz, 2H), 7.65 – 7.61 (m, 2H), 7.57 (t, J = 7.6 Hz, 2H), 7.53 – 7.45 (m, 4H), 6.40 (s, 1H), 5.34 (d, J = 1.0 Hz, 2H), 1.32 (s, 9H). ^{13}C NMR (101 MHz, $CDCl_3$) δ 160.14, 158.78, 157.32, 151.96, 143.36, 132.44, 130.76, 124.47, 122.51, 129.55, 128.43, 127.99, 127.76, 126.62, 115.22, 113.70, 113.47, 110.18, 100.82, 66.50, 35.52, 31.10. HRMS (ESI) calcd for $C_{28}H_{25}O_6S^+$ [M+H]$^+$ 489.1366, found 489.1359.

(7-oxo-3-phenyl-7H-furo[3,2-g]chromen-5-yl)methyl naphthalene-2-sulfonate (**7s**)

Yield 80%, m.p. 191-193 °C. ^{1}H NMR (400 MHz, $CDCl_3$) δ 8.50 (d, J = 1.2 Hz, 1H), 7.96 – 7.88 (m, 4H), 7.87 – 7.82 (m, 2H), 7.70 (td, J = 7.9, 0.8 Hz, 2H), 7.66 – 7.60 (m, 3H), 7.55 (t, J = 7.5 Hz, 2H), 7.47 (t, J = 7.3 Hz, 1H), 7.43 (s, 1H), 6.44 (s, 1H), 5.36 (d, J = 1.0 Hz, 2H). ^{13}C NMR (101 MHz, $CDCl_3$) δ 160.09, 157.27, 151.59, 146.89, 143.34, 135.61, 132.21, 131.95,

130.75, 130.22, 130.06, 129.95, 129.53, 129.47, 128.42, 128.19, 128.19, 127.74, 124.41, 122.46, 122.34, 115.16, 113.82, 113.39, 100.75, 66.81. HRMS (ESI) calcd for $C_{28}H_{19}O_6S^+$ [M+H]$^+$ 483.0897, found 483.0901.

(7-oxo-3-phenyl-7H-furo[3,2-g]chromen-5-yl)methyl-2-(trifluoromethyl)benzenesulfonate (**7t**)

Yield 88%, m.p. 153-154 ºC. ^{1}H NMR (400 MHz, $CDCl_3$) δ 8.29 (d, *J* = 7.5 Hz, 1H), 7.93 – 7.89 (m, 2H), 7.85 (s, 1H), 7.83 – 7.72 (m, 2H), 7.65 – 7.60 (m, 2H), 7.58 – 7.51 (m, 3H), 7.46 (t, *J* = 7.3 Hz, 1H), 6.47 (s, 1H), 5.45 (d, *J* = 1.0 Hz, 2H). ^{13}C NMR (101 MHz, $CDCl_3$) δ 160.13, 157.37, 151.97, 150.35, 146.57, 143.39, 134.65, 132.71, 132.28, 130.71, 129.50, 128.97 (q, *J* = 6.2 Hz), 128.40, 127.77, 124.54, 122.54, 120.94, 115.13, 113.89, 113.31, 100.89, 67.02. HRMS (ESI) calcd for $C_{25}H_{16}F_3O_6S^+$ [M+H]$^+$ 501.0614, found 501.0607.

(7-oxo-3-phenyl-7H-furo[3,2-g]chromen-5-yl)methyl-3-(trifluoromethyl)benzenesulfonate (**7u**)

Yield 92%, m.p. 180-182 ºC. ^{1}H NMR (400 MHz, $CDCl_3$) δ 8.17 (s, 1H), 8.11 (d, *J* = 8.0 Hz, 1H), 7.93 – 7.84 (m, 3H), 7.69 (t, *J* = 7.9 Hz, 1H), 7.64 – 7.59 (m, 2H), 7.55 (t, *J* = 7.5 Hz, 2H), 7.51 (s, 1H), 7.47 (t, *J* = 7.3 Hz, 1H), 6.43 (s, 1H), 5.40 (d, *J* = 1.0 Hz, 2H). ^{13}C NMR (101 MHz, $CDCl_3$) δ 159.94, 157.39, 151.95, 146.42, 143.48, 136.97, 132.57, 132.23, 131.25 (dd, *J* = 6.8, 3.2 Hz), 130.68, 130.52, 129.52, 128.46, 127.74, 125.16 (q, *J* = 3.8 Hz), 124.59, 122.46, 115.01, 113.97, 113.19, 100.94, 67.17. HRMS (ESI) calcd for $C_{25}H_{16}F_3O_6S^+$ [M+H]$^+$ 501.0614, found 501.0620.

(7-oxo-3-phenyl-7H-furo[3,2-g]chromen-5-yl)methyl -2-methylbenzenesulfonate (**7v**)

Yield 90%, m.p. 173-175 ºC. ^{1}H NMR (400 MHz, $CDCl_3$) δ 8.00 (d, *J* = 8.0 Hz, 1H), 7.89 (s, 1H), 7.85 (s, 1H), 7.65 – 7.59 (m, 2H), 7.58 – 7.44 (m, 6H), 7.34 (t, *J* = 8.0 Hz, 1H), 6.41 (s, 1H), 5.31 (s, 2H), 2.58 (s, 3H). ^{13}C NMR (101 MHz, $CDCl_3$) δ 160.13, 157.34, 151.98, 146.88, 143.39, 138.71, 134.54, 134.07, 132.90, 130.,23, 130.06, 129.52, 128.41, 128.13, 127.74, 126.54, 124.47, 122.49, 115.19, 113.85, 100.84, 66.52, 20.49. HRMS (ESI) calcd for $C_{25}H_{19}O_6S^+$ [M+H]$^+$ 447.0897, found 447.0910.

(7-oxo-3-phenyl-7H-furo[3,2-g]chromen-5-yl)methyl-3,5-difluorobenzenesulfonate (**7w**)

Yield 86%, m.p. 172-174 ºC. ^{1}H NMR (400 MHz, $CDCl_3$) δ 7.86 (d, *J* = 3.0 Hz, 2H), 7.64 – 7.60 (m, 2H), 7.56 (t, *J* = 7.5 Hz, 3H), 7.50 – 7.46 (m, 3H), 7.16 – 7.09 (m, 1H), 6.46 (s, 1H),

5.39 (d, *J* = 0.7 Hz, 2H). ^{13}C NMR (101 MHz, $CDCl_3$) *δ* 163.95, 159.99, 157.44, 152.02, 146.37, 143.51, 130.68, 129.55, 128.49, 127.78, 124.64, 122.50, 114.93, 113.84, 113.22, 111.89 (d, *J* = 6.5 Hz), 111.74 (d, *J* = 6.5 Hz), 110.44 (t, *J* = 24.8 Hz), 101.04, 67.25. HRMS (ESI) calcd for $C_{24}H_{15}F_2O_6S^+$ $[M+H]^+$ 469.0552, found 469.0539.

(7-oxo-3-phenyl-7H-furo[3,2-g]chromen-5-yl)methyl-3,5-dichlorobenzenesulfonate (**7x**)

Yield 83%, m.p. 161-163 °C. ^1H NMR (400 MHz, $CDCl_3$) *δ* 7.88 (s, 1H), 7.86 (s, 1H), 7.78 (d, *J* = 1.8 Hz, 2H), 7.65 – 7.60 (m, 3H), 7.59 – 7.53 (m, 3H), 7.47 (t, *J* = 7.3 Hz, 1H), 6.47 (s, 1H), 5.39 (d, *J* = 0.9 Hz, 2H). ^{13}C NMR (101 MHz, $CDCl_3$) *δ* 159.92, 157.42, 152.01, 146.26, 143.50, 138.39, 136.72, 134.61, 130.67, 129.55, 128.48, 127.76, 126.36, 124.63, 122.50, 115.03, 114.11, 113.19, 101.00, 67.47. HRMS (ESI) calcd for $C_{24}H_{15}Cl_2O_6S^+$ $[M+H]^+$ 500.9961, found 500.9954.

(7-oxo-3-phenyl-7H-furo[3,2-g]chromen-5-yl)methyl-2,4-difluorobenzenesulfonate (**7y**)

Yield 82%, m.p. 202-203 °C. ^1H NMR (400 MHz, $CDCl_3$) δ 7.99 – 7.92 (m, 1H), 7.91 (s, 1H), 7.86 (s, 1H), 7.65 – 7.61 (m, 2H), 7.58 – 7.52 (m, 3H), 7.46 (t, *J* = 7.3 Hz, 1H), 7.06 – 7.00 (m, 1H), 6.93 – 6.86 (m, 1H), 6.50 (s, 1H), 5.48 (d, *J* = 1.0 Hz, 2H). ^{13}C NMR (101 MHz, $CDCl_3$) *δ* 160.05, 157.39, 151.97, 146.56, 143.45, 132.78 (d, *J* = 10.9 Hz), 130.70, 129.52, 128.42, 127.78, 124.55, 122.51, 115.10, 113.93, 113.27, 112.65 (d, J = 3.7 Hz), 112.42 (d, *J* = 3.8 Hz), 106.40 (d, *J* = 24.3 Hz), 106.14 (d, *J* = 24.4 Hz), 100.90, 67.52. HRMS (ESI) calcd for $C_{24}H_{15}F_2O_6S^+$ $[M+H]^+$ 469.0552, found 469.0561.

(7-oxo-3-phenyl-7H-furo[3,2-g]chromen-5-yl)methyl-2,6-dichlorobenzenesulfonate (**7z**)

Yield 77%, m.p. 190-191 °C. ^1H NMR (400 MHz, $CDCl_3$) *δ* 7.95 (s, 1H), 7.85 (s, 1H), 7.65 – 7.61 (m, 2H), 7.55 (t, *J* = 7.5 Hz, 2H), 7.50 (s, 1H), 7.46 (t, *J* = 7.3 Hz, 1H), 7.42 – 7.38 (m, 2H), 7.37 – 7.32 (m, 1H), 6.53 (s, 1H), 5.47 (d, *J* = 1.0 Hz, 2H). ^{13}C NMR (101 MHz, $CDCl_3$) *δ* 160.01, 157.35, 151.97, 146.18, 143.40, 136.16, 134.08, 132.33, 131.64, 130.69, 129.53, 128.42, 127.76, 124.49, 122.54, 115.46, 114.54, 113.30, 100.78, 67.81. HRMS (ESI) calcd for $C_{24}H_{15}Cl_2O_6S^+$ $[M+H]^+$ 500.9961, found 500.9973.

(7-oxo-3-phenyl-7H-furo[3,2-g]chromen-5-yl)methyl-2,4-dichlorobenzenesulfonate (**7aa**)

Yield 85%, m.p. 210-212 °C. ^{1}H NMR (400 MHz, $CDCl_3$) δ 8.01 (d, *J* = 8.5 Hz, 1H), 7.90 (s, 1H), 7.86 (s, 1H), 7.64 – 7.60 (m, 2H), 7.58 – 7.52 (m, 3H), 7.49 – 7.44 (m, 2H), 7.40 (dd, *J* = 8.5, 2.0 Hz, 1H), 6.52 (s, 1H), 5.44 (d, *J* = 1.0 Hz, 2H). ^{13}C NMR (101 MHz, $CDCl_3$) δ 160.03, 157.39, 151.95, 146.37, 143.46, 141.68, 134.27, 132.71, 132.47, 132.16, 130.72, 129.52, 128.43, 127.79, 127.75, 124.54, 122.51, 115.19, 114.20, 113.28, 100.88, 67.55. HRMS (ESI) calcd for $C_{24}H_{15}Cl_2O_6S^+$ [M+H]$^+$ 500.9961, found 500.9949.

(7-oxo-3-phenyl-7H-furo[3,2-g]chromen-5-yl)methyl-2,5-difluorobenzenesulfonate (**7ab**)

Yield 81%, m.p. 150-152 °C. ^{1}H NMR (400 MHz, $CDCl_3$) δ 8.06 (s, 1H), 7.86 (s, 1H), 7.76 – 7.67 (m, 3H), 7.65 – 7.61 (m, 2H), 7.57 – 7.51 (m, 3H), 7.45 (t, *J* = 7.4 Hz, 1H), 6.57 (s, 1H), 4.75 (d, *J* = 0.7 Hz, 2H). ^{13}C NMR (101 MHz, $CDCl_3$) δ 160.32 (d, *J* = 2.5 Hz), 157.67 (d, *J* = 2.0 Hz), 152.21, 149.94, 143.34, 137.71 (t, *J* = 11.1 Hz), 130.93, 129.47, 128.33, 127.71, 124.39, 122.50, 115.72, 114.58, 114.06, 113.89 (d, *J* = 3.3 Hz), 113.80 (d, *J* = 26.0 Hz), 113.71 (d, *J* = 3.2 Hz), 100.85, 41.89. HRMS (ESI) calcd for $C_{24}H_{15}F_2O_6S^+$ [M+H]$^+$ 469.0552, found 469.0564.

(7-oxo-3-phenyl-7H-furo[3,2-g]chromen-5-yl)methyl-3-fluoro-4-methylbenzenesulfonate (**7ac**)

Yield 80%, m.p. 198-200 °C. ^{1}H NMR (400 MHz, $CDCl_3$) δ 7.88 (s, 1H), 7.86 (s, 1H), 7.65 – 7.59 (m, 3H), 7.59 – 7.51 (m, 4H), 7.47 (t, *J* = 7.3 Hz, 1H), 7.36 (t, *J* = 7.5 Hz, 1H), 6.42 (s, 1H), 5.34 (d, *J* = 0.8 Hz, 2H), 2.36 (d, *J* = 1.7 Hz, 3H). ^{13}C NMR (151 MHz, $CDCl_3$) δ 161.77, 160.69, 157.37, 151.98, 146.79, 143.43, 134.37 (d, *J* = 6.8 Hz), 133.18 (d, *J* = 17.4 Hz), 132.75 (d, *J* = 4.7 Hz), 130.72, 129.54, 128.45 127.77, 124.54, 123.68 (d, *J* = 3.8 Hz), 122.50, 115.08 (t, *J* = 13.0 Hz), 113.67, 113.35, 100.89, 66.75, 15.09. HRMS (ESI) calcd for $C_{25}H_{18}FO_6S^+$ [M+H]$^+$ 465.0803, found 465.0789.

(7-oxo-3-phenyl-7H-furo[3,2-g]chromen-5-yl)methyl-2,5-dimethylbenzenesulfonate (**7ad**)

Yield 82%, m.p. 167-168 °C. ^{1}H NMR (400 MHz, $CDCl_3$) δ 7.91 (s, 1H), 7.85 (s, 1H), 7.80 (s, 1H), 7.64 – 7.60 (m, 2H), 7.55 (t, *J* = 7.5 Hz, 2H), 7.51 (s, 1H), 7.47 (t, *J* = 7.4 Hz, 1H), 7.30 (d, *J* = 7.8 Hz, 1H), 7.14 (d, *J* = 7.7 Hz, 1H), 6.42 (s, 1H), 5.30 (d, *J* = 0.8 Hz, 2H), 2.51 (s, 3H), 2.37 (s, 3H). ^{13}C NMR (101 MHz, $CDCl_3$) δ 160.14, 157.35, 151.99, 146.91, 143.38, 136.67, 135.48, 135.24, 133.72, 132.78, 130.73, 130.38, 129.53, 128.42, 127.75, 124.46, 122.50,

115.24, 113.94, 113.51, 100.82, 66.50, 20.90, 20.00. HRMS (ESI) calcd for $C_{26}H_{21}O_6S^+$ [M+H]$^+$ 461.1053, found 461.1043.

NMR and other chemical characterizations of **8a-8ag**

(7-oxo-3-phenyl-7H-furo[3,2-g]chromen-5-yl)methyl- 4-fluorobenzoate (**8a**)

Yield 92%, m.p. 173-175 °C. 1H NMR (600 MHz, $CDCl_3$) δ 8.18 – 8.13 (m, 2H), 7.96 (s, 1H), 7.85 (s, 1H), 7.62 – 7.57 (m, 3H), 7.51 (t, *J* = 7.6 Hz, 2H), 7.44 (t, *J* = 7.4 Hz, 1H), 7.21 – 7.13 (m, 2H), 6.59 (s, 1H), 5.63 (d, *J* = 1.0 Hz, 2H). ^{13}C NMR (151 MHz, $CDCl_3$) δ 167.25, 164.87, 160.72, 157.32, 152.04, 149.55, 143.34, 132.63 (d, *J* = 9.4 Hz), 130.84, 129.46, 128.36, 127.72, 125.33 (d, *J* = 2.9 Hz), 124.49, 122.48, 116.12 (d, *J* = 22.1 Hz), 114.87, 111.82, 100.91, 62.13. HRMS (ESI) calcd for $C_{25}H_{16}FO_5^+$ [M+H]$^+$ 415.0976, found 415.0950.

(7-oxo-3-phenyl-7H-furo[3,2-g]chromen-5-yl)methyl- 4-methoxybenzoate (**8b**)

Yield 78%, m.p. 186-187 °C. ^{1}H NMR (400 MHz, $CDCl_3$) δ 8.09 (d, *J* = 8.6 Hz, 2H), 7.98 (s, 1H), 7.85 (s, 1H), 7.62 – 7.56 (m, 3H), 7.50 (t, *J* = 7.5 Hz, 2H), 7.43 (t, *J* = 7.2 Hz, 1H), 6.97 (d, *J* = 8.6 Hz, 2H), 6.61 (s, 1H), 5.61 (s, 2H), 3.89 (s, 3H). ^{13}C NMR (151 MHz, $CDCl_3$) δ 165.56, 164.21, 160.84, 157.30, 152.05, 149.99, 143.27, 132.99, 132.13, 130.87, 129.47, 128.34, 127.73, 124.44, 122.51, 121.40, 114.97, 114.13, 111.76, 100.85, 61.78, 55.70. HRMS (ESI) calcd for $C_{26}H_{19}O_6^+$ [M+H]$^+$ 427.1176, found 427.1190.

(7-oxo-3-phenyl-7H-furo[3,2-g]chromen-5-yl)methyl-[1,1'-biphenyl]-4-carboxylate (**8c**)

Yield 80%, m.p. 187-189 °C. 1H NMR (600 MHz, $CDCl_3$) δ 8.20 (d, *J* = 7.8 Hz, 2H), 8.00 (s, 1H), 7.86 (s, 1H), 7.72 (d, *J* = 7.8 Hz, 2H), 7.66 – 7.58 (m, 5H), 7.50 (dd, *J* = 16.3, 8.1 Hz, 4H), 7.43 (dd, *J* = 16.8, 8.0 Hz, 2H), 6.64 (s, 1H), 5.66 (s, 2H). ^{13}C NMR (151 MHz, $CDCl_3$) δ 165.75, 160.79, 157.32, 152.07, 149.73, 146.77, 143.30, 139.88, 131.30, 130.86, 130.54, 129.47, 129.16, 128.55, 128.36, 127.74, 127.51, 127.48, 124.48, 122.52, 114.97, 114.00, 111.90, 100.89, 62.09. HRMS (ESI) calcd for $C_{31}H_{21}O_5^+$ [M+H]$^+$ 473.1384, found 473.1366.

(7-oxo-3-phenyl-7H-furo[3,2-g]chromen-5-yl)methyl -4-methylbenzoate (**8d**)

Yield 85%,m.p. 154-155 °C. ^{1}H NMR (600 MHz, $CDCl_3$) δ 8.02 (d, *J* = 8.2 Hz, 2H), 7.97 (s, 1H), 7.85 (s, 1H), 7.61 – 7.56 (m, 3H), 7.50 (t, *J* = 7.6 Hz, 2H), 7.43 (t, *J* = 7.4 Hz, 1H), 7.29 (d, *J* = 8.0 Hz, 2H), 6.61 (s, 1H), 5.62 (d, *J* = 1.2 Hz, 2H), 2.45 (s, 3H). ^{13}C NMR (151 MHz, $CDCl^3$) δ 165.90, 160.81, 157.29, 152.04, 149.84, 144.87, 143.27, 130.86, 130.03, 129.56, 129.46,

128.33, 127.72, 126.34, 124.43, 122.50, 114.96, 114.00, 111.81, 100.85, 61.89, 21.91. HRMS (ESI) calcd for $C_{26}H_{19}O_5^+$ [M+H]$^+$ 411.1227, found 411.1242.

(7-oxo-3-phenyl-7H-furo[3,2-g]chromen-5-yl)methyl-4-(trifluoromethoxy)benzoate (**8e**)

Yield 82%, m.p. 150-152 °C. ^{1}H NMR (600 MHz, $CDCl_3$) δ 8.18 (d, *J* = 8.8 Hz, 2H), 7.96 (s, 1H), 7.86 (s, 1H), 7.62 – 7.57 (m, 3H), 7.51 (t, *J* = 7.5 Hz, 2H), 7.44 (t, *J* = 7.4 Hz, 1H), 7.33 (d, *J* = 8.4 Hz, 2H), 6.58 (s, 1H), 5.65 (d, *J* = 0.9 Hz, 2H). ^{13}C NMR (151 MHz, $CDCl_3$) δ 164.68, 160.68, 157.35, 153.41, 152.06, 149.39, 143.37, 132.38, 132.06, 130.84, 129.47, 128.38, 127.74, 127.40, 124.52, 122.49, 120.68, 114.85, 113.87, 111.90, 100.95, 62.30. HRMS (ESI) calcd for $C_{26}H_{16}F_3O_6^+$ [M+H]$^+$ 481.0893, found 481.0907.

(7-oxo-3-phenyl-7H-furo[3,2-g]chromen-5-yl)methyl -4-bromobenzoate (**8f**)

Yield 83%, m.p. 174-176 °C. ^{1}H NMR (400 MHz, $CDCl_3$) δ 7.99 (d, J = 8.2 Hz, 2H), 7.95 (s, 1H), 7.85 (s, 1H), 7.64 (d, *J* = 8.3 Hz, 2H), 7.61 – 7.57 (m, 3H), 7.51 (t, *J* = 7.5 Hz, 2H), 7.44 (t, *J* = 7.7 Hz, 1H), 6.58 (s, 1H), 5.63 (s, 2H). ^{13}C NMR (151 MHz, $CDCl_3$) δ 165.17, 160.67, 157.33, 152.05, 149.39, 143.35, 132.26, 131.45, 130.83, 129.47, 129.29, 128.38, 127.96, 127.73, 124.50, 122.48, 114.86, 113.88, 111.90, 100.93, 62.26. HRMS (ESI) calcd for $C_{25}H_{16}BrO_5^+$ [M+H]$^+$ 475.0176, found 475.0193.

(7-oxo-3-phenyl-7H-furo[3,2-g]chromen-5-yl)methyl -4-chlorobenzoate (**8g**)

Yield 88%, m.p. 210-211°C. ^{1}H NMR (600 MHz, $CDCl_3$) δ 8.07 (d, J = 8.6 Hz, 2H), 7.96 (s, 1H), 7.86 (s, 1H), 7.61 – 7.57 (m, 3H), 7.51 (t, *J* = 7.5 Hz, 2H), 7.48 (d, *J* = 8.6 Hz, 2H), 7.44 (t, *J* = 7.4 Hz, 1H), 6.58 (s, 1H), 5.63 (d, *J* = 1.2 Hz, 2H). ^{13}C NMR (151 MHz, $CDCl_3$) δ 165.02, 160.69, 157.33, 152.04, 149.43, 143.35, 140.59, 131.36, 130.83, 129.47, 129.26, 128.38, 127.73, 127.51, 124.50, 122.48, 114.86, 113.88, 111.88, 100.93, 62.23. HRMS (ESI) calcd for $C_{25}H_{16}ClO_5^+$ [M+H]$^+$ 431.0681, found 431.0695.

(7-oxo-3-phenyl-7H-furo[3,2-g]chromen-5-yl)methyl benzoate (**8h**)

Yield 79%, m.p. 146-147 °C. ^{1}H NMR (400 MHz, $CDCl_3$) δ 8.14 (d, *J* = 7.1 Hz, 2H), 7.98 (s, 1H), 7.85 (s, 1H), 7.64 (t, *J* = 7.4 Hz, 1H), 7.62 – 7.56 (m, 3H), 7.50 (td, *J* = 7.7, 3.3 Hz, 4H), 7.43 (t, *J* = 7.3 Hz, 1H), 6.62 (s, 1H), 5.64 (d, *J* = 1.3 Hz, 2H). ^{13}C NMR (101 MHz, $CDCl_3$) δ 165.83, 160.78, 157.27, 152.00, 149.68, 143.28, 133.98, 130.82, 129.98, 129.45, 129.05, 128.85, 128.33, 127.70, 124.44, 122.47, 114.92, 113.93, 111.80, 100.87, 77.48, 77.16, 76.84, 62.05. HRMS (ESI) calcd for $C_{25}H_{17}O_5^+$ [M+H]$^+$ 397.1071, found 397.1059.

(7-oxo-3-phenyl-7H-furo[3,2-g]chromen-5-yl)methyl-4-(trifluoromethyl)benzoate (**8i**)

Yield 81%, m.p. 175-176 °C. ^{1}H NMR (400 MHz, $CDCl_3$) δ 8.25 (d, *J* = 8.1 Hz, 2H), 7.96 (s, 1H), 7.86 (s, 1H), 7.77 (d, *J* = 8.2 Hz, 2H), 7.63 – 7.57 (m, 3H), 7.51 (t, *J* = 7.6 Hz, 2H), 7.44 (t, *J* = 6.9 Hz, 1H), 6.59 (s, 1H), 5.67 (s, 2H). ^{13}C NMR (151 MHz, $CDCl_3$) δ 164.69, 160.60, 157.36, 152.06, 149.15, 143.39, 135.45 (q, *J* = 33.1 Hz), 132.29, 130.82, 130.42, 129.47, 128.40, 127.73, 125.92 (q, *J* = 3.7 Hz), 124.54, 122.48, 114.82, 113.83, 111.98, 100.98, 62.52. HRMS (ESI) calcd for $C_{26}H_{16}F_3O_5^+$ [M+H]$^+$ 465.0944, found 465.0923.

(7-oxo-3-phenyl-7H-furo[3,2-g]chromen-5-yl)methyl -3-chlorobenzoate (**8j**)

Yield 76%, m.p. 84-86 °C. ^{1}H NMR (600 MHz, $CDCl_3$) δ 8.10 (s, 1H), 8.02 (d, *J* = 7.6 Hz, 1H), 7.96 (s, 1H), 7.85 (s, 1H), 7.63 – 7.56 (m, 4H), 7.51 (t, *J* = 7.2 Hz, 2H), 7.45 (t, *J* = 6.7 Hz, 2H), 6.58 (s, 1H), 5.64 (s, 2H). ^{13}C NMR (151 MHz, $CDCl_3$) δ 164.70, 160.65, 157.33, 152.06, 149.27, 143.35, 135.10, 134.03, 130.83, 130.20, 130.02, 129.48, 128.38, 128.09, 127.74, 124.52, 122.50, 114.89, 113.88, 112.03, 100.93, 62.40. HRMS (ESI) calcd for $C_{25}H_{16}ClO_5^+$ [M+H]$^+$ 431.0681, found 431.0668.

(7-oxo-3-phenyl-7H-furo[3,2-g]chromen-5-yl)methyl -3-bromobenzoate (**8k**)

Yield 89%, m.p. 87-89 °C. ^{1}H NMR (400 MHz, $CDCl_3$) δ 8.26 (s, 1H), 8.06 (dd, *J* = 7.8, 0.6 Hz, 1H), 7.96 (s, 1H), 7.85 (s, 1H), 7.77 (dd, *J* = 8.0, 0.9 Hz, 1H), 7.63 – 7.57 (m, 3H), 7.52 (t, *J* = 7.5 Hz, 2H), 7.44 (t, *J* = 7.1 Hz, 1H), 7.38 (t, *J* = 7.9 Hz, 1H), 6.58 (s, 1H), 5.64 (s, 2H). ^{13}C NMR (101 MHz, $CDCl_3$) δ 164.57, 160.64, 157.33, 152.06, 149.25, 143.35, 136.94, 132.92, 130.99, 130.83, 130.42, 129.48, 128.53, 128.38, 127.74, 124.52, 122.97, 122.50, 114.89, 113.88, 112.05, 100.94, 62.41. HRMS (ESI) calcd for $C_{25}H_{16}BrO_5^+$ [M+H]$^+$ 475.0176, found 475.0164.

(7-oxo-3-phenyl-7H-furo[3,2-g]chromen-5-yl)methyl- 2-chlorobenzoate (**8l**)

Yield 90%, m.p. 147-149 °C. ^{1}H NMR (400 MHz, $CDCl_3$) δ 7.98 – 7.93 (m, 2H), 7.85 (s, 1H), 7.63 – 7.56 (m, 3H), 7.55 – 7.48 (m, 4H), 7.44 (t, *J* = 7.3 Hz, 1H), 7.37 (td, *J* = 6.9 Hz, *J* = 6.9 Hz, 2.2 Hz, 1H), 6.65 (s, 1H), 5.65 (s, 2H). ^{13}C NMR (101 MHz, $CDCl_3$) δ 164.87, 160.69, 157.27, 151.98, 149.09, 143.29, 134.37, 133.58, 131.90, 131.59, 130.80, 129.47, 128.75, 128.34, 127.69, 126.99, 124.43, 122.46, 114.90, 113.85, 112.15, 100.86, 62.57. HRMS (ESI) calcd for $C_{25}H_{16}ClO_5^+$ [M+H]$^+$ 431.0681, found 431.0662.

(7-oxo-3-phenyl-7H-furo[3,2-g]chromen-5-yl)methyl -2-fluorobenzoate (**8m**)

Yield 86%,m.p. 149-151 °C. ^{1}H NMR (400 MHz, $CDCl_3$) δ 7.98 (s, 1H), 7.93 – 7.89 (m, 1H), 7.86 (s, 1H), 7.74 – 7.70 (m, 1H), 7.63 – 7.57 (m, 3H), 7.53 (t, *J* = 7.6 Hz, 2H), 7.48 – 7.39

(m, 3H), 6.66 (s, 1H), 5.65 (s, 2H). ^{13}C NMR (101 MHz, $CDCl_3$) δ 165.35, 160.70, 157.30, 152.01, 149.04, 143.31, 134.89, 133.55, 131.79, 130.82 (d, J = 1.4 Hz), 129.50, 128.37, 127.72, 127.57, 124.47, 122.49, 122.30, 114.94, 113.88, 112.25, 100.89, 62.63. HRMS (ESI) calcd for $C_{25}H_{16}FO_5^+$ [M+H]$^+$ 415.0976, found 415.0987.

(7-oxo-3-phenyl-7H-furo[3,2-g]chromen-5-yl)methyl- 3-fluorobenzoate (**8n**)

Yield 84%, m.p. 126-127 °C. ^{1}H NMR (400 MHz, $CDCl_3$) δ 7.97 (s, 1H), 7.93 (d, *J* = 7.8 Hz, 1H), 7.85 (s, 1H), 7.81 (dd, *J* = 9.1, 1.3 Hz, 1H), 7.62 – 7.57 (m, 3H), 7.54 – 7.44 (m, 4H), 7.35 (td, *J* = 8.3, 2.5 Hz, 1H), 6.59 (s, 1H), 5.64 (s, 2H). ^{13}C NMR (101 MHz, $CDCl_3$) δ 164.75 (d, J = 3.0 Hz), 164.03, 161.56, 160.65, 157.33, 152.06, 149.31, 143.34, 130.83, 130.58 (d, *J* = 7.8 Hz), 129.47, 128.38, 127.74, 125.73 (d, *J* = 3.1 Hz), 124.52, 122.51, 121.13 (d, *J* = 21.2 Hz), 116.90 (d, *J* = 23.2 Hz), 114.89, 113.89, 112.01, 100.93, 62.39. HRMS (ESI) calcd for $C_{25}H_{16}FO_5^+$ [M+H]$^+$ 415.0976, found 415.0992.

(7-oxo-3-phenyl-7H-furo[3,2-g]chromen-5-yl)methyl -2-bromobenzoate (**8o**)

Yield 88%,m.p. 123-125 °C. ^{1}H NMR (400 MHz, $CDCl_3$) δ 7.98 (s, 1H), 7.93 – 7.89 (m, 1H), 7.86 (s, 1H), 7.74 – 7.70 (m, 1H), 7.63 – 7.56 (m, 3H), 7.52 (t, *J* = 7.5 Hz, 2H), 7.48 – 7.39 (m, 3H), 6.65 (s, 1H), 5.65 (s, 2H). ^{13}C NMR (101 MHz, $CDCl_3$) δ 165.34, 160.70, 157.29, 152.00, 149.04, 143.30, 134.88, 133.55, 131.79, 130.80, 129.49, 128.36, 127.71, 127.56, 124.46, 122.48, 122.29, 114.93, 113.86, 112.23, 100.88, 62.63. HRMS (ESI) calcd for $C_{25}H_{16}BrO_5^+$ [M+H]$^+$ 475.0176, found 475.0158.

(7-oxo-3-phenyl-7H-furo[3,2-g]chromen-5-yl)methyl -3-methylbenzoate (**8p**)

Yield 80%, m.p. 183-185 °C. ^{1}H NMR (400 MHz, $CDCl_3$) δ 7.99 (s, 1H), 7.94 (s, 2H), 7.85 (s, 1H), 7.63 – 7.57 (m, 3H), 7.53 – 7.35 (m, 5H), 6.62 (s, 1H), 5.63 (s, 2H), 2.43 (s, 3H). ^{13}C NMR (101 MHz, $CDCl_3$) δ 166.03, 160.79, 157.30, 152.05, 149.75, 143.28, 138.75, 134.75, 130.86, 130.45, 129.46, 129.02, 128.74, 128.34, 127.72, 127.16, 124.45, 122.51, 114.97, 114.00, 111.87, 100.87, 62.06. 21.46. HRMS (ESI) calcd for $C_{26}H_{19}O_5^+$ [M+H]$^+$ 411.1227, found 411.1210.

(7-oxo-3-phenyl-7H-furo[3,2-g]chromen-5-yl)methyl -4-nitrobenzoate (**8q**)

Yield 85%, m.p. 194-196 °C. ^{1}H NMR (600 MHz, $CDCl_3$) δ8.34 (d, *J* = 8.3 Hz, 2H), 8.30 (d, *J* = 8.5 Hz, 2H), 7.96 (s, 1H), 7.86 (s, 1H), 7.60 (d, *J* = 9.3 Hz, 3H), 7.52 (t, *J* = 7.4 Hz, 2H), 7.45 (t, *J* = 6.9 Hz, 1H), 6.58 (s, 1H), 5.69 (s, 2H). ^{13}C NMR (151 MHz, $CDCl_3$) δ 164.05, 160.53, 157.38, 152.05, 151.17, 148.87, 143.45, 134.37, 131.15, 130.81, 129.49, 128.42, 127.74,

124.58, 124.01, 122.46, 114.75, 113.74, 112.05, 101.03, 62.80. HRMS (ESI) calcd for $C_{25}H_{16}NO_7^+$ [M+H]$^+$ 442.0921, found 442.0902

(7-oxo-3-phenyl-7H-furo[3,2-g]chromen-5-yl)methyl -4-(tert-butyl)benzoate (**8r**)

Yield 82%, m.p. 108-110 °C. ^{1}H NMR (400 MHz, $CDCl_3$) δ 8.06 (d, *J* = 8.4 Hz, 2H), 7.99 (s, 1H), 7.85 (s, 1H), 7.62 – 7.56 (m, 3H), 7.54 – 7.40 (m, 5H), 6.60 (s, 1H), 5.63 (s, 2H), 1.36 (s, 9H). ^{13}C NMR (151 MHz, $CDCl_3$) δ 165.86, 160.78, 157.84, 157.30, 152.06, 149.87, 143.26, 130.87, 129.92, 129.45, 128.32, 127.73, 126.27, 125.84, 124.42, 122.51, 114.99, 114.02, 111.83, 100.84, 61.90, 35.37, 31.23. HRMS (ESI) calcd for $C_{29}H_{25}O_5^+$ [M+H]$^+$ 453.1697, found 453.1712.

(7-oxo-3-phenyl-7H-furo[3,2-g]chromen-5-yl)methyl- 2-naphthoate (**8s**)

Yield 77%, m.p. 146-147 °C. ^{1}H NMR (400 MHz, $CDCl_3$) δ 8.71 (s, 1H), 8.13 (d, *J* = 8.7 Hz, 1H), 8.03 (s, 1H), 8.00 – 7.90 (m, 3H), 7.86 (s, 1H), 7.67 – 7.56 (m, 5H), 7.49 (t, *J* = 7.6 Hz, 2H), 7.43 (t, *J* = 7.1 Hz, 1H), 6.69 (s, 1H), 5.70 (s, 2H). ^{13}C NMR (101 MHz, $CDCl_3$) δ 166.03, 160.79, 157.32, 152.08, 149.72, 143.31, 136.02, 132.60, 131.76, 130.86, 129.63, 129.47, 128.94, 128.74, 128.35, 128.00, 127.73, 127.14, 126.28, 125.17, 124.48, 122.52, 115.00, 114.03, 111.99, 100.90, 62.24. HRMS (ESI) calcd for $C_{29}H_{19}O_5^+$ [M+H]$^+$ 447.1227, found 447.1212.

(7-oxo-3-phenyl-7H-furo[3,2-g]chromen-5-yl)methyl -3,5-dichlorobenzoate (**8t**)

Yield 75%, m.p. 163-165 °C. ^{1}H NMR (400 MHz, $CDCl^3$) δ 7.98 (d, *J* = 0.9 Hz, 2H), 7.94 (s, 1H), 7.86 (s, 1H), 7.63 – 7.57 (m, 4H), 7.53 (t, *J* = 7.5 Hz, 2H), 7.45 (t, *J* = 7.3 Hz, 1H), 6.55 (s, 1H), 5.64 (s, 2H). ^{13}C NMR (101 MHz, $CDCl_3$) δ 163.64, 160.57, 157.32, 152.02, 148.89, 143.39, 135.86, 133.83, 131.82, 130.79, 129.48, 128.40, 128.30, 127.73, 124.55, 122.47, 114.80, 113.76, 112.11, 100.98, 62.72. HRMS (ESI) calcd for $C_{25}H_{15}Cl_2O_5^+$ [M+H]$^+$ 465.0291, found 465.0314.

(7-oxo-3-phenyl-7H-furo[3,2-g]chromen-5-yl)methyl-3-(trifluoromethyl)benzoate (**8u**)

Yield 80%, m.p. 137-138 °C. ^{1}H NMR (400 MHz, $CDCl_3$) δ 8.38 (s, 1H), 8.32 (d, *J* = 7.8 Hz, 1H), 7.97 (s, 1H), 7.90 (d, *J* = 7.8 Hz, 1H), 7.86 (s, 1H), 7.66 (t, *J* = 7.9 Hz, 1H), 7.62 – 7.57 (m, 3H), 7.51 (t, *J* = 7.5 Hz, 2H), 7.44 (t, *J* = 7.3 Hz, 1H), 6.57 (s, 1H), 5.68 (s, 2H). ^{13}C NMR (101 MHz, $CDCl_3$) δ 164.61, 160.59, 157.35, 152.06, 149.14, 143.37, 133.11, 131.83, 131.51, 130.82, 130.48 (d, J = 3.6 Hz), 129.98, 129.61, 129.46, 128.38, 127.73, 126.92 (q, *J* = 3.9

Hz), 124.54, 122.50, 114.84, 113.85, 112.09, 100.96, 62.50. HRMS (ESI) calcd for $C_{26}H_{16}F_3O_5^+$ $[M+H]^+$ 465.0944, found 465.0965.

(7-oxo-3-phenyl-7H-furo[3,2-g]chromen-5-yl)methyl -2-methylbenzoate (**8v**)

Yield 83%, m.p. 139-141 °C. ^1H NMR (400 MHz, $CDCl_3$) δ 8.06 (d, *J* = 8.0 Hz, 1H), 7.98 (s, 1H), 7.85 (s, 1H), 7.62 – 7.56 (m, 3H), 7.53 – 7.42 (m, 4H), 7.31 (d, *J* = 7.6 Hz, 2H), 6.61 (s, 1H), 5.61 (s, 2H), 2.64 (s, 3H). ^{13}C NMR (101 MHz, $CDCl_3$) δ 166.40, 160.79, 157.26, 152.01, 149.79, 143.27, 141.30, 133.06, 132.21, 130.90, 130.83, 129.45, 128.32, 128.08, 127.70, 126.17, 124.42, 122.47, 114.93, 113.99, 111.90, 100.85, 61.84, 22.09. HRMS (ESI) calcd for $C_{26}H_{19}O_5^+$ $[M+H]^+$ 411.1227, found 411.1240.

(7-oxo-3-phenyl-7H-furo[3,2-g]chromen-5-yl)methyl -3-nitrobenzoate (**8w**)

Yield 86%, m.p. 172-174 °C. ^1H NMR (400 MHz, $CDCl_3$) δ 8.95 (s, 1H), 8.50 (d, *J* = 8.1 Hz, 1H), 8.45 (d, *J* = 7.5 Hz, 1H), 7.97 (s, 1H), 7.86 (s, 1H), 7.73 (t, *J* = 8.0 Hz, 1H), 7.64 – 7.57 (m, 3H), 7.52 (t, *J* = 7.4 Hz, 2H), 7.45 (t, *J* = 7.3 Hz, 1H), 6.57 (s, 1H), 5.71 (s, 2H). ^{13}C NMR (101 MHz, $CDCl_3$) δ 163.86, 160.51, 157.37, 152.07, 148.87, 148.61, 143.42, 135.49, 130.83 (d, J = 4.1 Hz), 130.20, 129.49, 128.39 (d, *J* = 5.2 Hz), 127.75, 124.99, 124.59, 122.49, 114.80, 113.78, 112.21, 101.02, 62.78. HRMS (ESI) calcd for $C_{25}H_{16}NO_7^+$ $[M+H]^+$ 442.0921, found 442.0939.

(7-oxo-3-phenyl-7H-furo[3,2-g]chromen-5-yl)methyl -3-methoxybenzoate (**8x**)

Yield 88%, m.p. 107-108 °C. ^1H NMR (400 MHz, $CDCl_3$) δ 7.99 (s, 1H), 7.85 (s, 1H), 7.73 (d, *J* = 7.1 Hz, 1H), 7.65 – 7.56 (m, 4H), 7.51 (t, *J* = 7.6 Hz, 2H), 7.47 – 7.38 (m, 2H), 7.17 (dd, *J* = 8.3, 2.3 Hz, 1H), 6.60 (s, 1H), 5.64 (s, 2H), 3.86 (s, 3H). ^{13}C NMR (101 MHz, $CDCl_3$) δ 165.73, 160.71, 159.91, 157.30, 152.05, 149.61, 143.29, 130.85, 130.34, 129.89, 129.46, 128.33, 127.72, 124.44, 122.49, 122.31, 120.28, 114.98, 114.60, 113.98, 111.98, 100.87, 77.48, 62.18, 55.65. HRMS (ESI) calcd for $C_{26}H_{19}O_6^+$ $[M+H]^+$ 427.1176, found 427.1153.

(7-oxo-3-phenyl-7H-furo[3,2-g]chromen-5-yl)methyl-3-(trifluoromethoxy)benzoate (**8y**)

Yield 92%, m.p. 121-123 °C. ^1H NMR (400 MHz, $CDCl_3$) δ 8.08 (d, J = 7.5 Hz, 1H), 7.96 (s, 2H), 7.86 (s, 1H), 7.61 – 7.48 (m, 7H), 7.43 (t, *J* = 7.1 Hz, 1H), 6.58 (s, 1H), 5.66 (s, 2H). ^{13}C NMR (101 MHz, $CDCl_3$) δ 164.54, 160.61, 157.35, 152.06, 149.58, 149.18, 143.36, 131.11, 130.83, 130.48, 129.46, 128.38, 128.25, 127.73, 126.37, 124.53, 122.50, 114.85, 113.86, 112.06, 100.96, 62.48. HRMS (ESI) calcd for $C_{26}H_{16}F_3O_6^+$ $[M+H]^+$ 481.0893, found 481.0879.

(7-oxo-3-phenyl-7H-furo[3,2-g]chromen-5-yl)methyl-3,5-bis(trifluoromethyl)benzoate (**8z**)

Yield 75%,m.p. 188-190 °C. ^{1}H NMR (400 MHz, $CDCl_3$) δ 8.55 (s, 2H), 8.14 (s, 1H), 7.96 (s, 1H), 7.86 (s, 1H), 7.63 – 7.58 (m, 3H), 7.52 (t, *J* = 7.5 Hz, 2H), 7.45 (t, *J* = 7.3 Hz, 1H), 6.53 (s, 1H), 5.72 (s, 2H). ^{13}C NMR (101 MHz, $CDCl_3$) δ 163.38, 160.45, 157.37, 152.04, 148.59, 143.46, 132.77 (q, J = 34.3 Hz). 131.25, 130.78, 130.03 (d, J = 3.4 Hz), 129.46, 128.42, 127.73, 127.35 (t, J = 4.0 Hz), 124.61, 122.46, 121.45, 114.74, 113.71, 112.34, 110.16, 101.05, 62.99. HRMS (ESI) calcd for $C_{27}H_{15}F_6O_5^+$ [M+H]$^+$ 533.0818, found 533.0836.

(7-oxo-3-phenyl-7H-furo[3,2-g]chromen-5-yl)methyl-2-chloro-6-fluorobenzoate (**8aa**)

Yield 81%, m.p. 156-157 °C. ^{1}H NMR (400 MHz, $CDCl_3$) δ 7.95 (s, 1H), 7.86 (s, 1H), 7.63 – 7.58 (m, 2H), 7.58 – 7.51 (m, 3H), 7.48 – 7.38 (m, 2H), 7.27 (d, *J* = 8.4 Hz, 1H), 7.10 (t, *J* = 8.7 Hz, 1H), 6.65 (s, 1H), 5.67 (s, 2H). ^{13}C NMR (101 MHz, $CDCl_3$) δ 162.64, 161.39, 160.63, 158.86, 157.30, 152.00, 148.42, 143.31, 132.59 (d, J = 9.4 Hz), 130.81, 129.48, 128.35, 127.74, 125.99 (d, *J* = 3.6 Hz), 124.46, 122.51, 114.94, 114.89, 114.73, 112.53, 100.89, 63.09. HRMS (ESI) calcd for $C_{25}H_{15}ClFO_5^+$ [M+H]$_+$ 449.0587, found 449.0568.

(7-oxo-3-phenyl-7H-furo[3,2-g]chromen-5-yl)methyl -3,4-dichlorobenzoate (**8ab**)

Yield 78%, m.p. 183-185 °C. ^{1}H NMR (400 MHz, $CDCl_3$) δ 8.20 (d, *J* = 1.2 Hz, 1H), 7.97 – 7.93 (m, 2H), 7.86 (s, 1H), 7.62 – 7.56 (m, 4H), 7.52 (t, *J* = 7.5 Hz, 2H), 7.45 (t, *J* = 7.3 Hz, 1H), 6.56 (s, 1H), 5.64 (s, 2H). ^{13}C NMR (101 MHz, $CDCl_3$) δ 164.07, 160.61, 157.32, 152.01, 149.09, 143.38, 138.81, 133.57, 131.82, 131.04, 130.80, 129.48, 128.95, 128.83, 128.39, 127.73, 124.53, 122.47, 114.79, 113.78, 111.98, 100.97, 62.52. HRMS (ESI) calcd for $C_{25}H_{15}Cl_2O_5^+$ [M+H]$^+$ 465.0291, found 465.0275.

(7-oxo-3-phenyl-7H-furo[3,2-g]chromen-5-yl)methyl -3,5-difluorobenzoate (**8ac**)

Yield 87%, m.p. 225-227 °C. ^{1}H NMR (400 MHz, $CDCl_3$) δ 7.94 (s, 1H), 7.86 (s, 1H), 7.68 – 7.57 (m, 5H), 7.53 (t, *J* = 7.5 Hz, 2H), 7.45 (t, *J* = 7.2 Hz, 1H), 7.13 – 7.07 (m, 1H), 6.56 (s, 1H), 5.65 (s, 2H). ^{13}C NMR (101 MHz, $CDCl_3$) δ 164.24, 160.58, 157.33, 152.03, 148.95, 143.39, 130.80, 129.48, 128.41, 127.74, 124.56, 122.48, 114.80, 113.77, 113.17 (d, *J* = 7.6 Hz), 112.98 (d, *J* = 7.7 Hz), 112.06, 109.52 (t, *J* = 25.1 Hz), 100.99, 62.69. HRMS (ESI) calcd for $C_{25}H_{15}F_2O_5^+$ [M+H]$^+$ 433.0882, found 433.0868.

(7-oxo-3-phenyl-7H-furo[3,2-g]chromen-5-yl)methyl -3,4-difluorobenzoate (**8ad**)

Yield 92%, m.p. 190-191°C. ^1H NMR (400 MHz, $CDCl_3$) δ 7.99 – 7.90 (m, 3H), 7.86 (s, 1H), 7.63 – 7.56 (m, 3H), 7.52 (t, J = 7.6 Hz, 2H), 7.48 – 7.42 (m, 1H), 7.33 – 7.27 (m, 1H), 6.56 (s, 1H), 5.64 (s, 2H). ^{13}C NMR (101 MHz, $CDCl_3$) δ 163.94, 160.62, 157.31, 155.57 (d, J = 12.5 Hz), 153.01 (d, J = 12.6 Hz), 151.99, 151.66 (d, J = 13.1 Hz), 149.19, 143.38, 130.79, 129.46, 128.38, 127.71, 127.05 (dd, J = 7.6, 3.8 Hz), 124.51, 122.45, 119.39 (dd, J = 18.8, 1.7 Hz), 117.96 (d, J = 17.9 Hz), 114.77, 113.77, 111.85, 100.95, 62.43. HRMS (ESI) calcd for $C_{25}H_{15}F_2O_5^+$ $[M+H]^+$ 433.0882, found 433.0911.

(7-oxo-3-phenyl-7H-furo[3,2-g]chromen-5-yl)methyl-3,4,5-trifluorobenzoate (**8ae**)

Yield 83%, m.p. 204-206 °C. ^1H NMR (400 MHz, $CDCl_3$) δ 7.93 (s, 1H), 7.86 (s, 1H), 7.77 (t, J = 6.9 Hz, 2H), 7.62 – 7.58 (m, 3H), 7.53 (t, J = 7.5 Hz, 2H), 7.45 (t, J = 7.3 Hz, 1H), 6.53 (s, 1H), 5.64 (d, J = 0.6 Hz, 2H). ^{13}C NMR (101 MHz, $CDCl_3$) δ 160.52, 157.34, 152.02, 148.84, 143.43, 130.78, 129.48, 128.42, 127.74, 124.58, 122.45, 114.79, 114.73, 114.63, 114.56, 113.70, 112.02, 101.02, 62.79. HRMS (ESI) calcd for $C_{25}H_{14}F_3O_5^+$ $[M+H]^+$ 451.0788, found 451.0765.

(7-oxo-3-phenyl-7H-furo[3,2-g]chromen-5-yl)methyl cyclohexanecarboxylate (**8af**)

Yield 70%,m.p. 119-120°C. ^1H NMR (400 MHz, $CDCl_3$) δ 7.88 (s, 1H), 7.85 (s, 1H), 7.63 – 7.58 (m, 2H), 7.57 – 7.50 (m, 3H), 7.45 (t, J = 7.4 Hz, 1H), 6.49 (s, 1H), 5.37 (s, 2H), 2.46 (tt, J = 11.5, 3.6 Hz, 1H), 1.98 (dd, J = 13.0, 2.4 Hz, 2H), 1.82 – 1.73 (m, 2H), 1.50 (ddd, J = 14.7, 12.4, 3.1 Hz, 2H), 1.39 – 1.16 (m, 4H). ^{13}C NMR (101 MHz, $CDCl_3$) δ 175.32, 160.81, 157.23, 151.97, 149.85, 143.25, 130.85, 129.45, 128.34, 127.72, 124.38, 122.46, 114.87, 113.97, 111.71, 100.81, 61.33, 43.26, 29.16, 25.75, 25.49. HRMS (ESI) calcd for $C_{25}H_{23}O_5^+$ $[M+H]^+$ 403.1540, found 403.1557.

(7-oxo-3-phenyl-7H-furo[3,2-g]chromen-5-yl)methyl furan-2-carboxylate (**8ag**)

Yield 68%,m.p. 161-163 °C. ^1H NMR (400 MHz, $CDCl_3$) δ 7.94 (s, 1H), 7.85 (s, 1H), 7.68 – 7.65 (m, 1H), 7.63 – 7.59 (m, 2H), 7.57 (s, 1H), 7.52 (t, J = 7.6 Hz, 2H), 7.44 (t, J = 7.3 Hz, 1H), 7.34 (d, J = 3.5 Hz, 1H), 6.61 – 6.57 (m, 2H), 5.61 (d, J = 0.6 Hz, 2H). ^{13}C NMR (101 MHz, $CDCl_3$) δ 160.70, 157.72, 157.27, 151.97, 149.28, 147.42, 143.64, 143.30, 130.82, 129.47, 128.34, 127.70, 124.41, 122.46, 119.54, 114.82, 113.79, 112.40, 111.76, 100.87, 61.67. HRMS (ESI) calcd for $C_{23}H_{15}O_6^+$ $[M+H]^+$ 387.0863, found 387.0876.

^{1}H NMR spectra of intermediate **3**

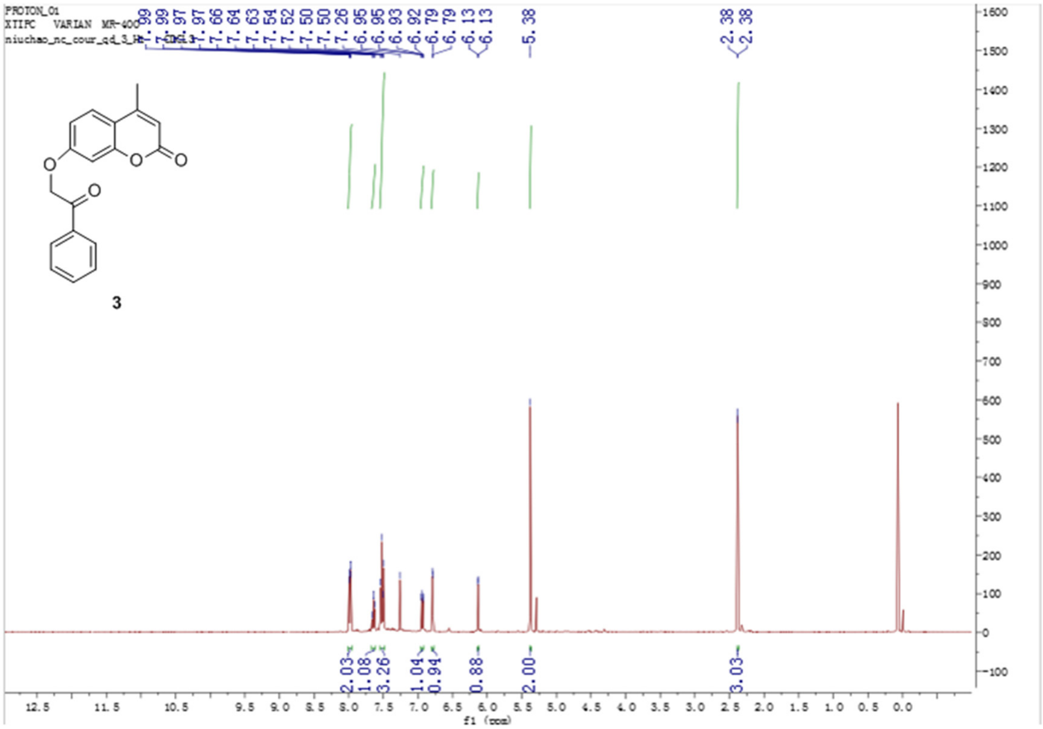

^{1}H NMR spectra of compound **4**

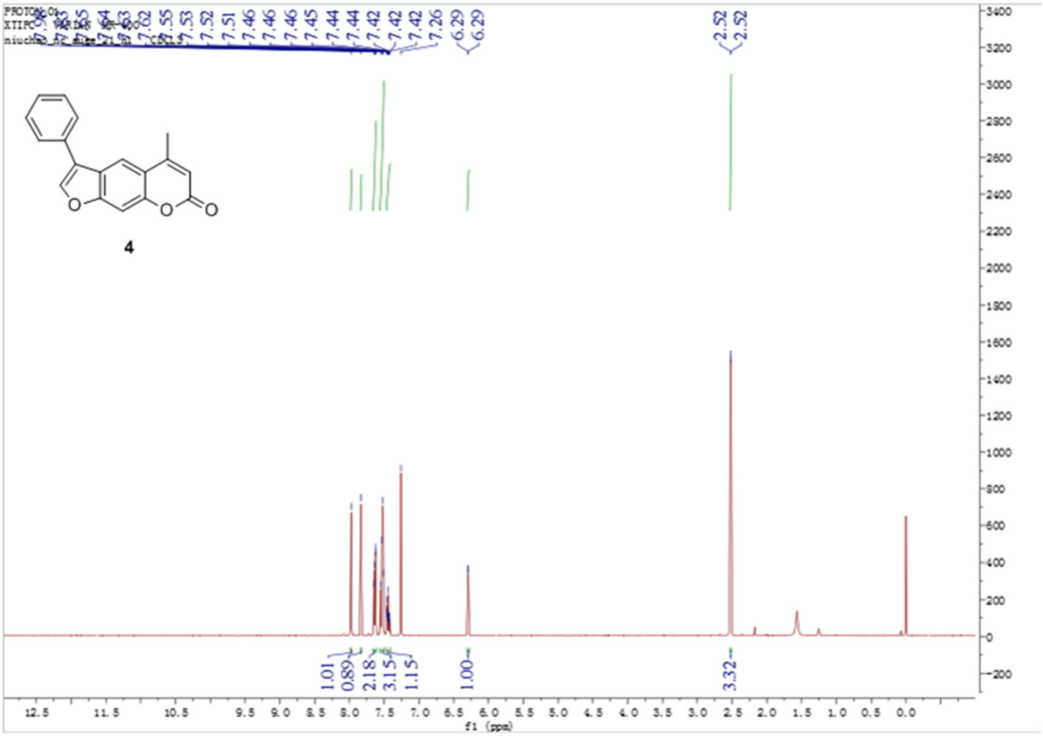

¹H NMR spectra of compound **5**

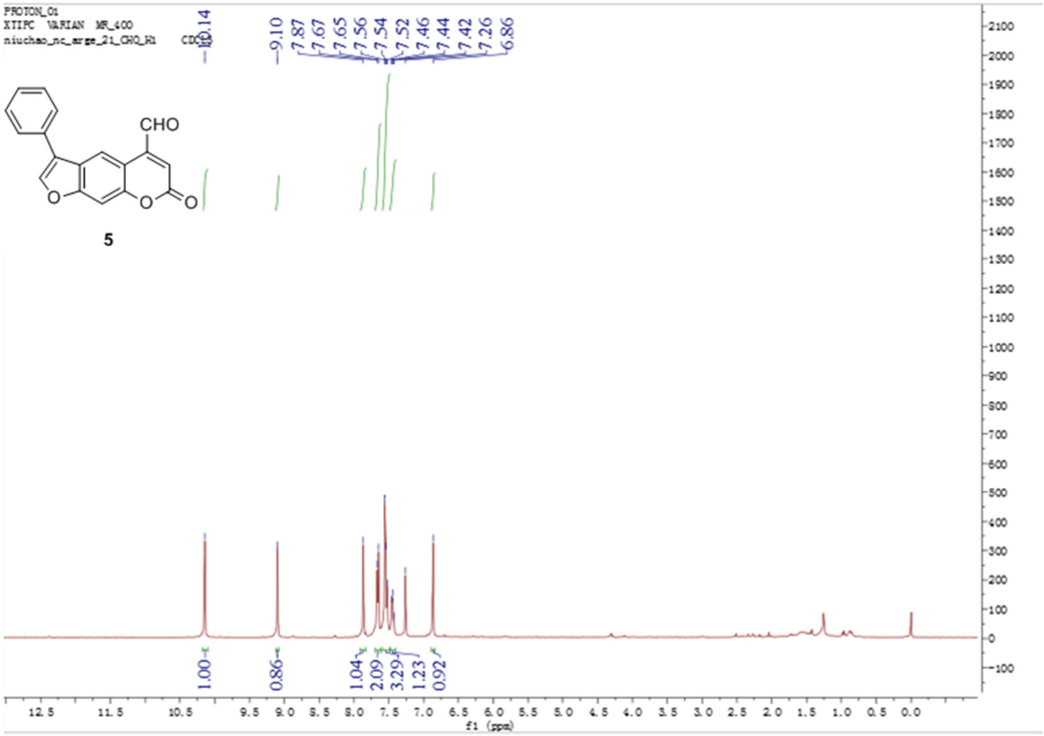

¹H NMR spectra of compound **6**

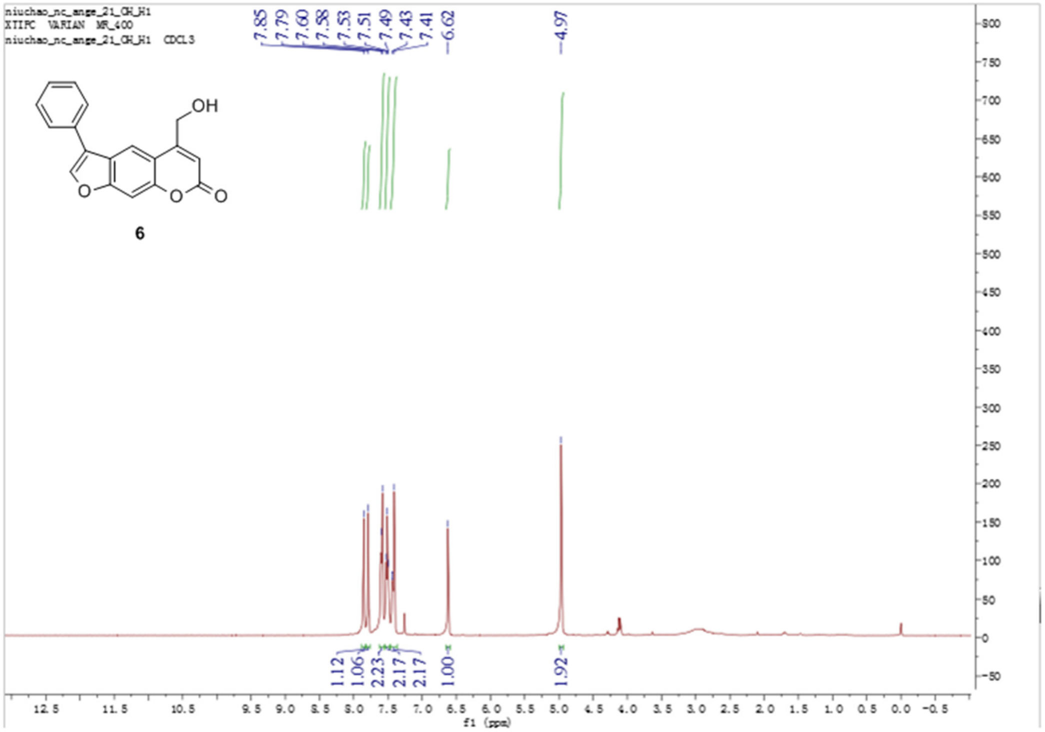

^1H and ^{13}C NMR spectra of **7**

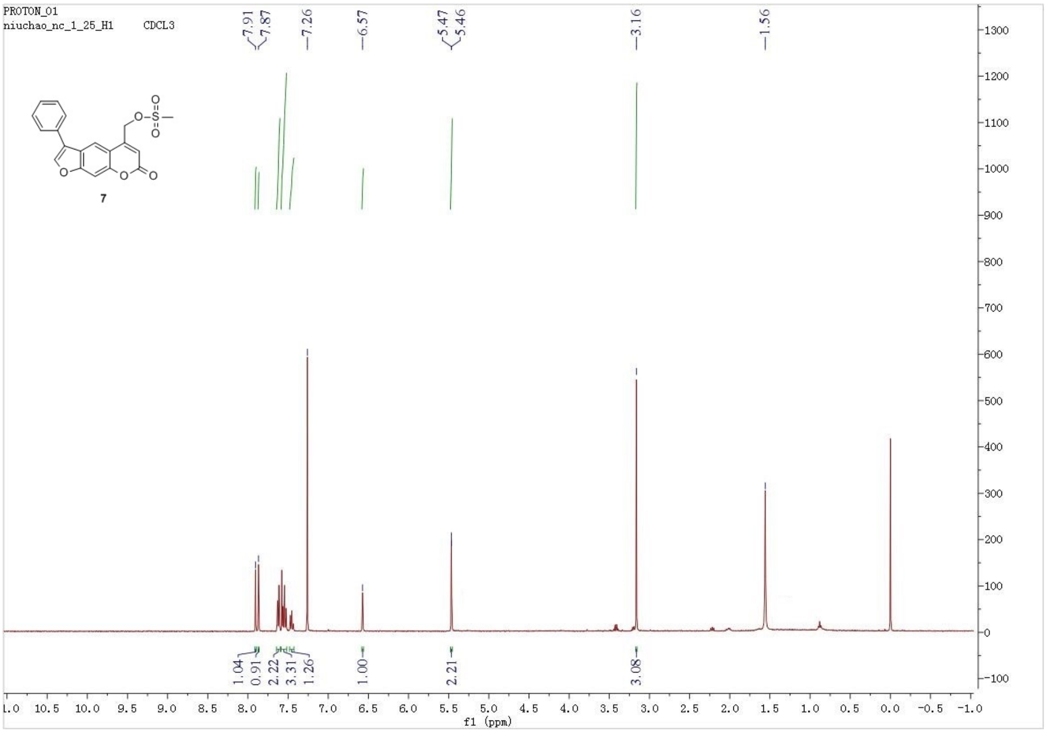

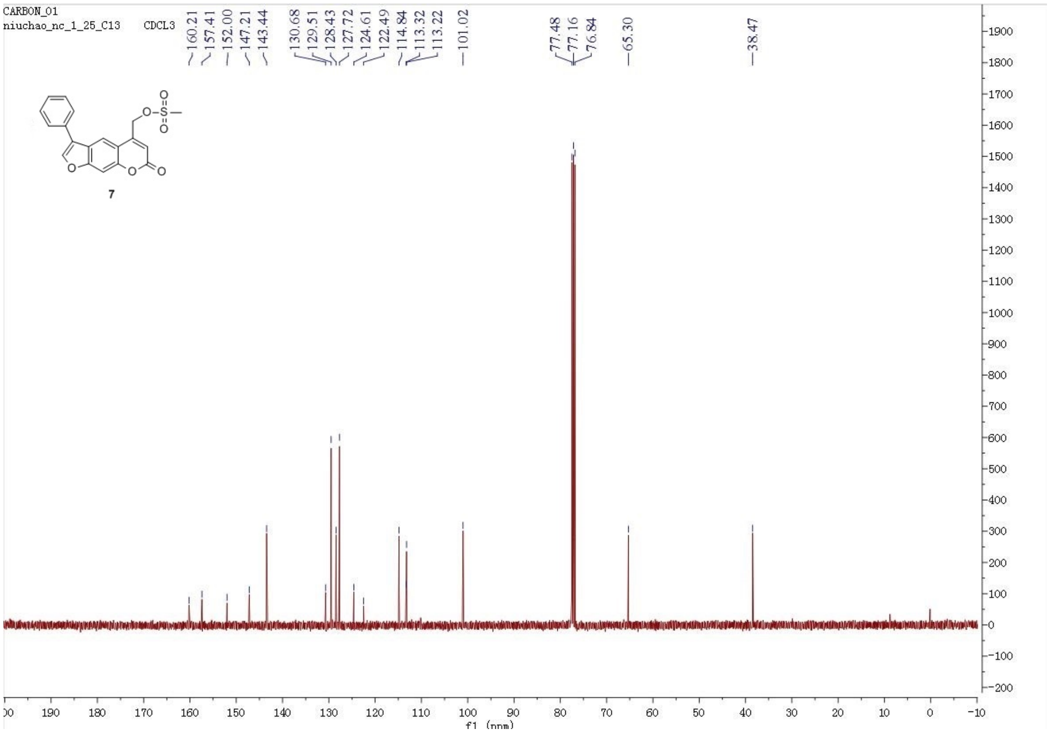

^1H and ^{13}C NMR spectra of **7a**

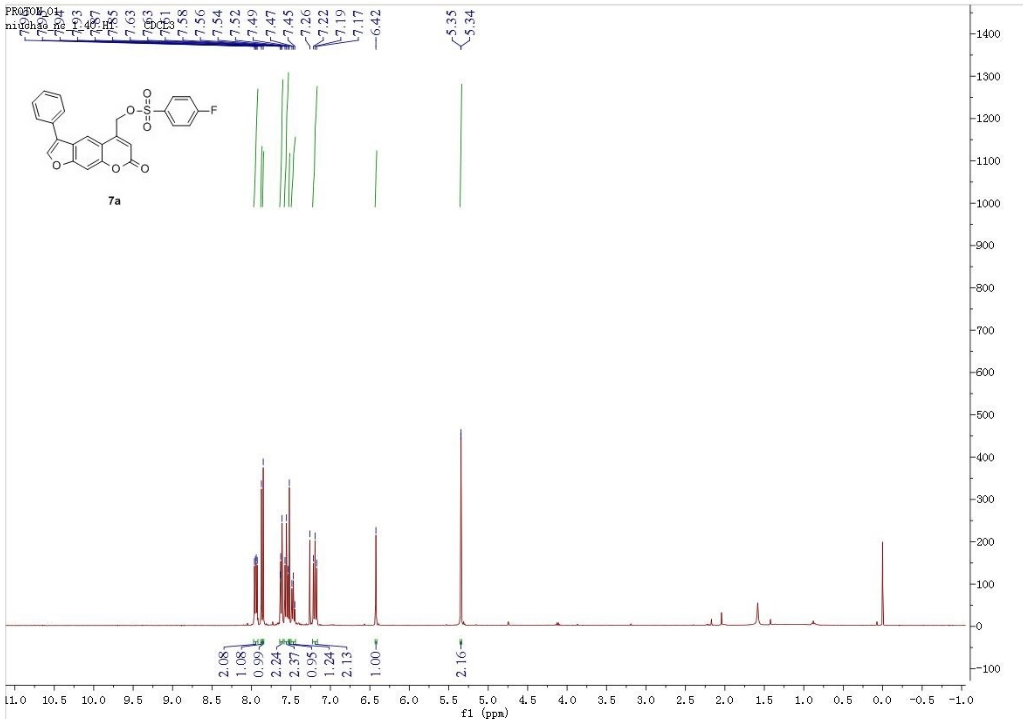

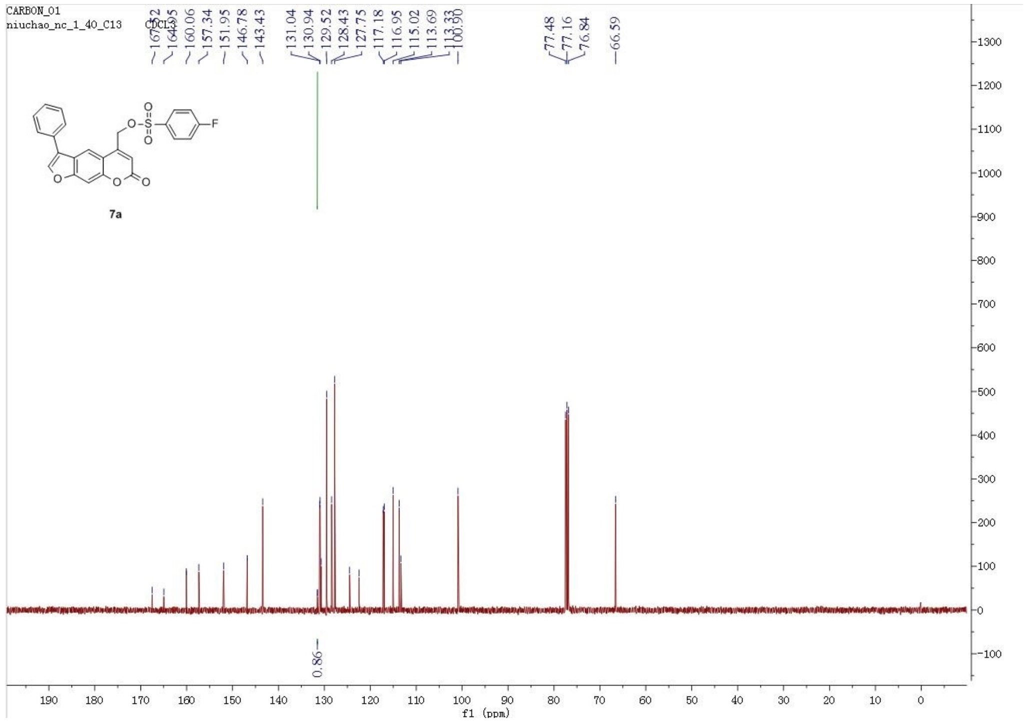

^{1}H NMR and ^{13}C NMR spectra of **7b**

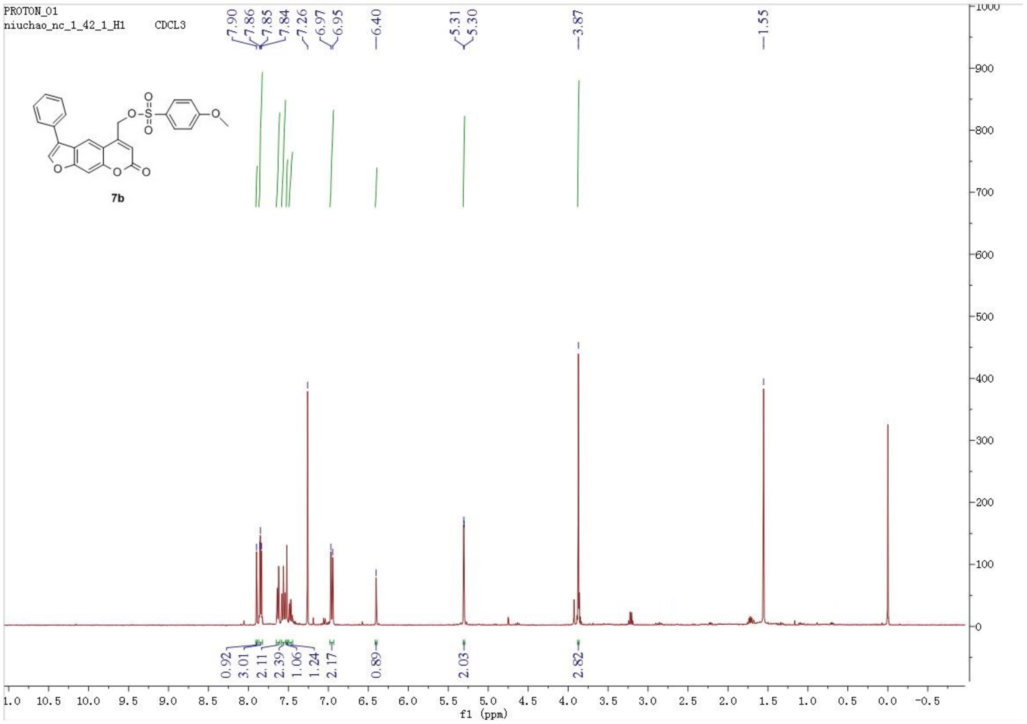

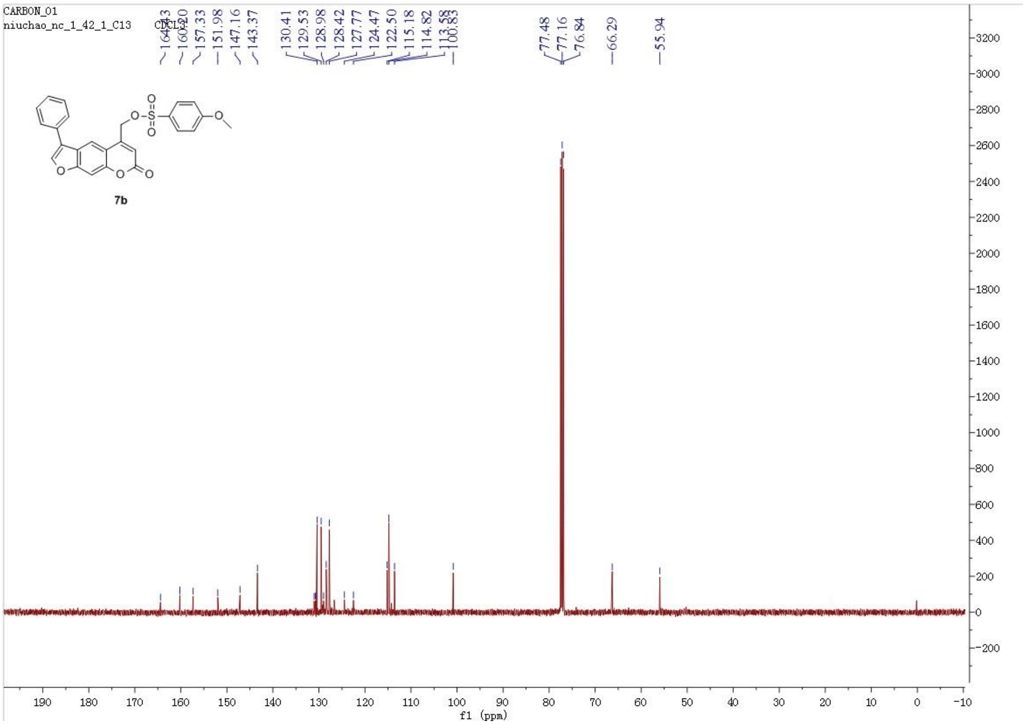

^{1}H NMR and ^{13}C NMR spectra of **7c**

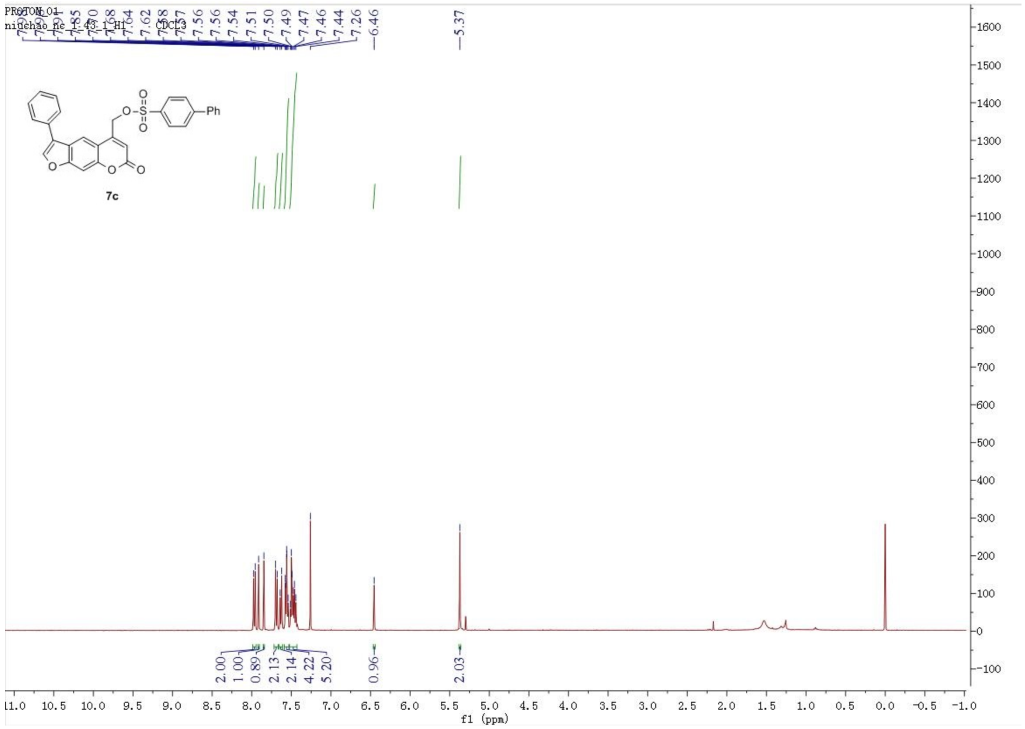

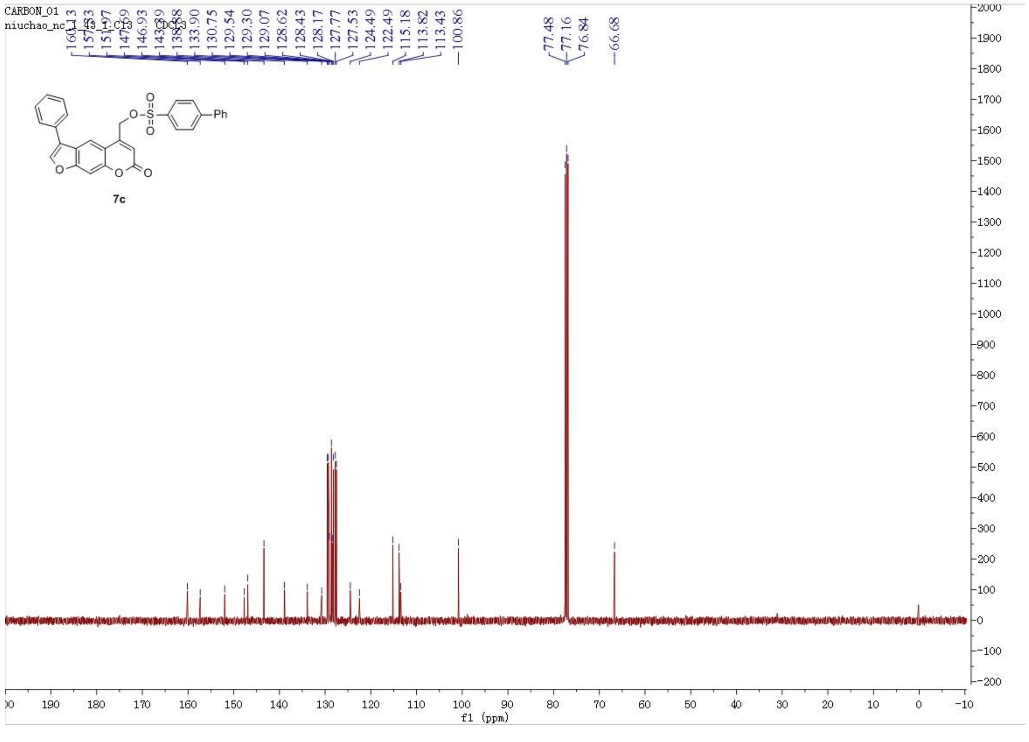

^{1}H NMR and ^{13}C NMR spectra of **7d**

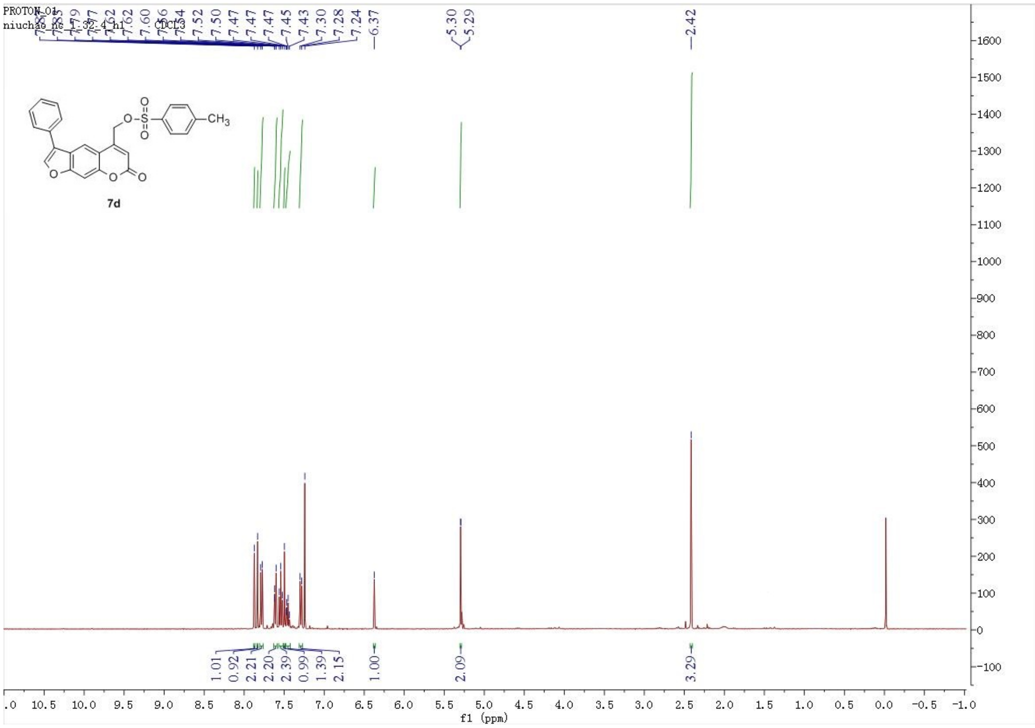

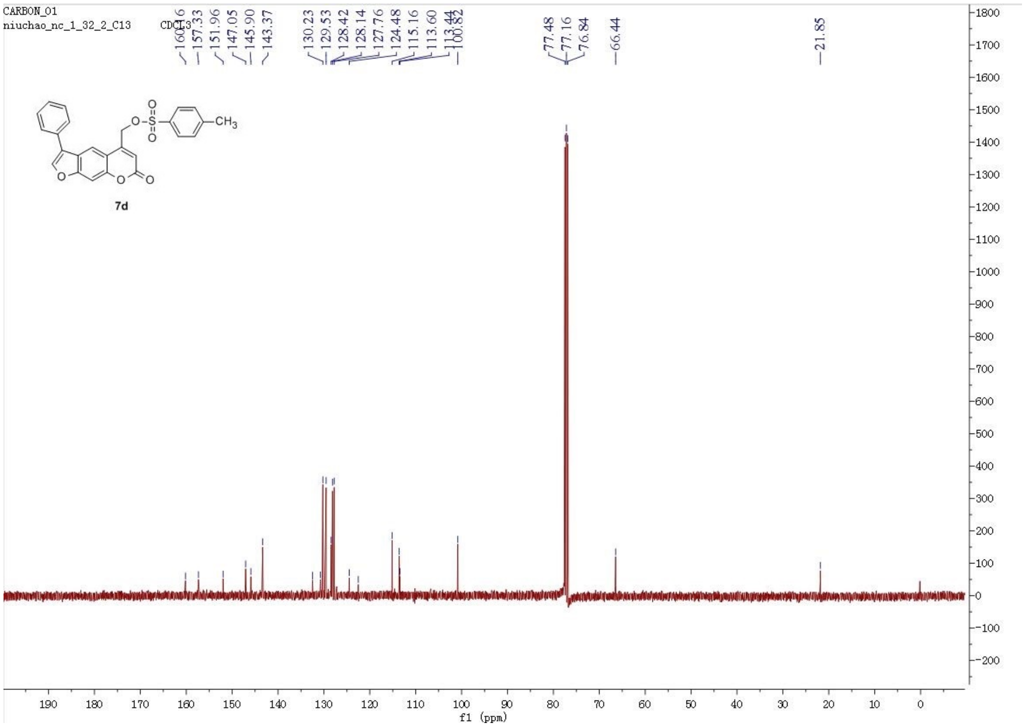

^{1}H NMR and ^{13}C NMR spectra of **7e**

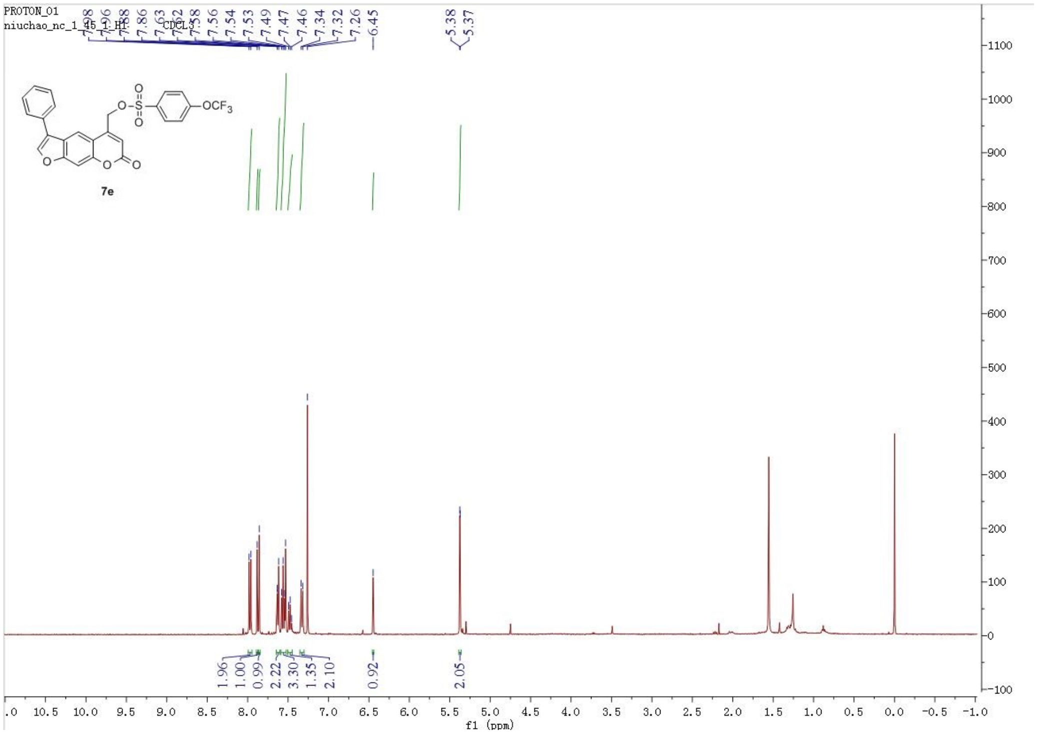

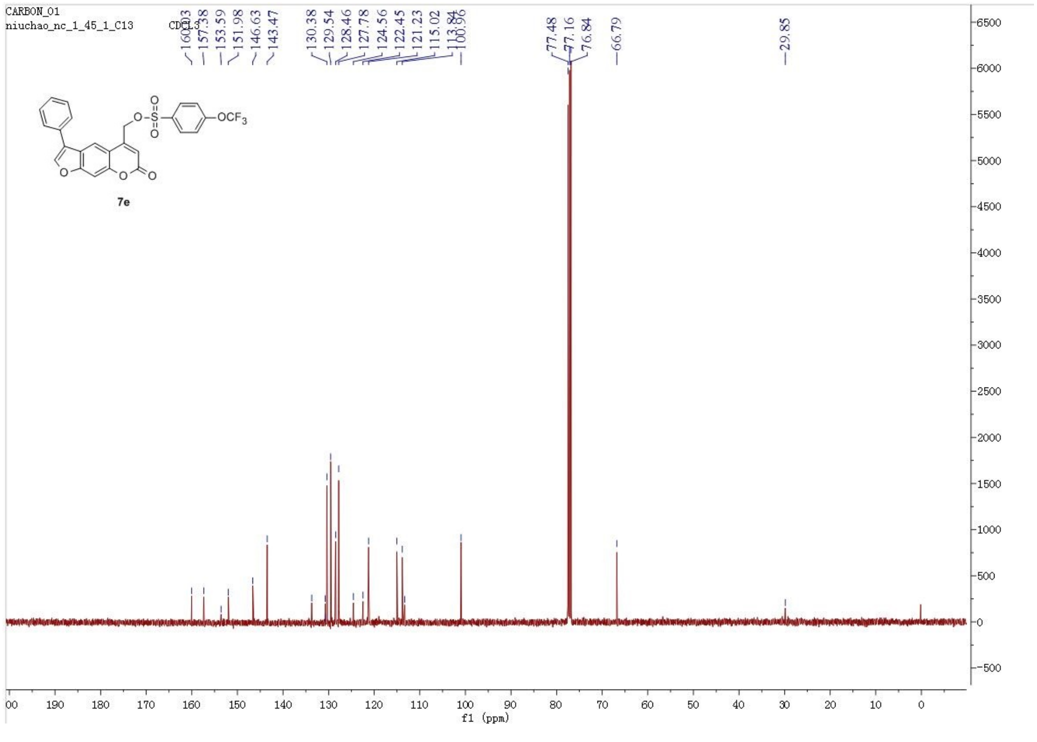

^{1}H NMR and ^{13}C NMR spectra of **7f**

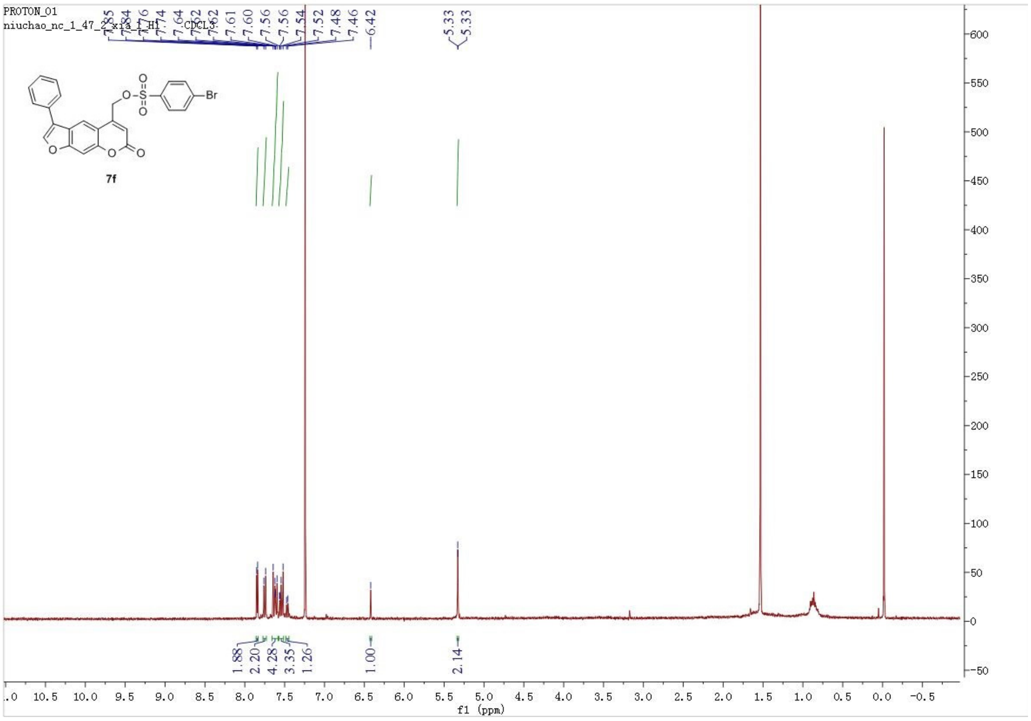

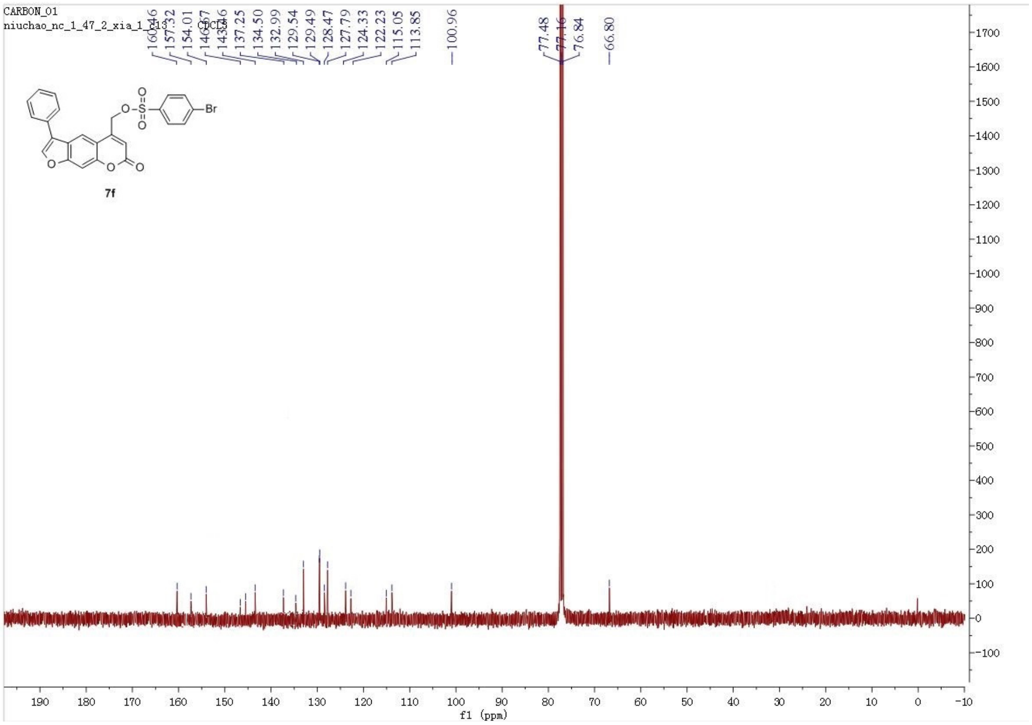

^{1}H NMR and ^{13}C NMR spectra of **7g**

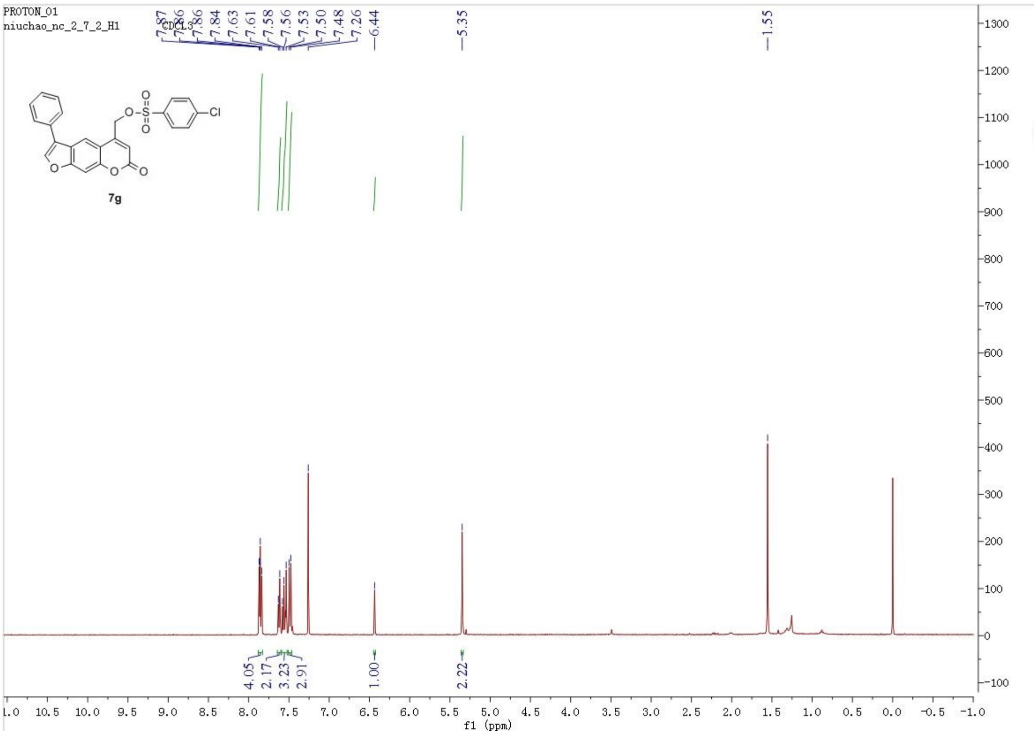

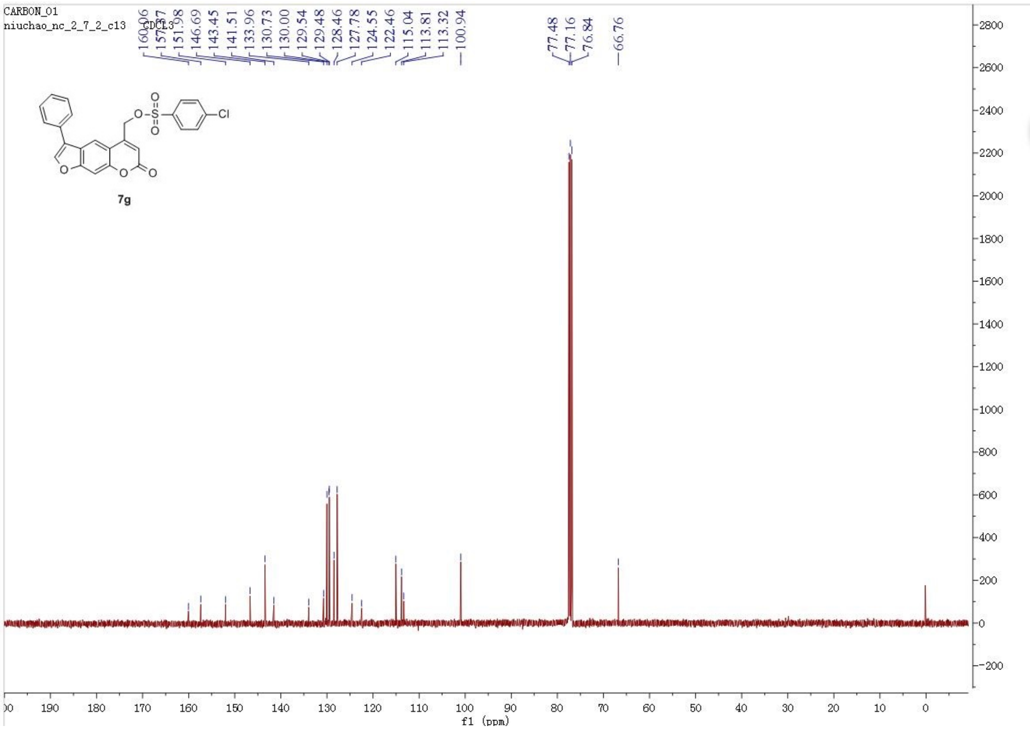

^{1}H NMR and ^{13}C NMR spectra of **7h**

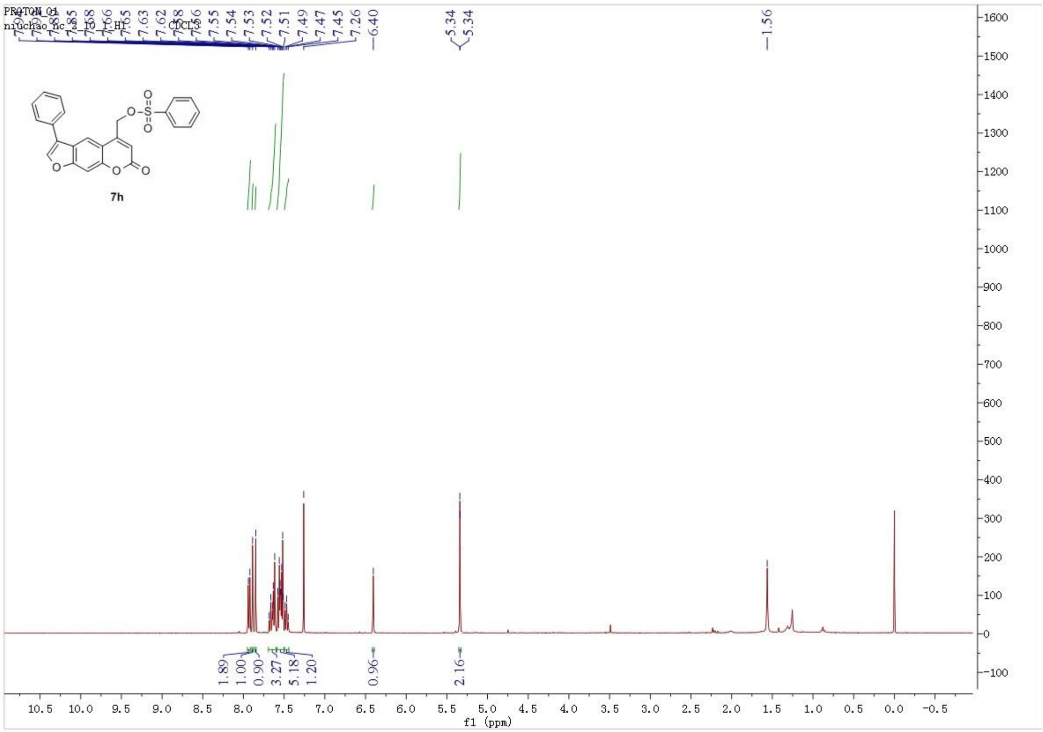

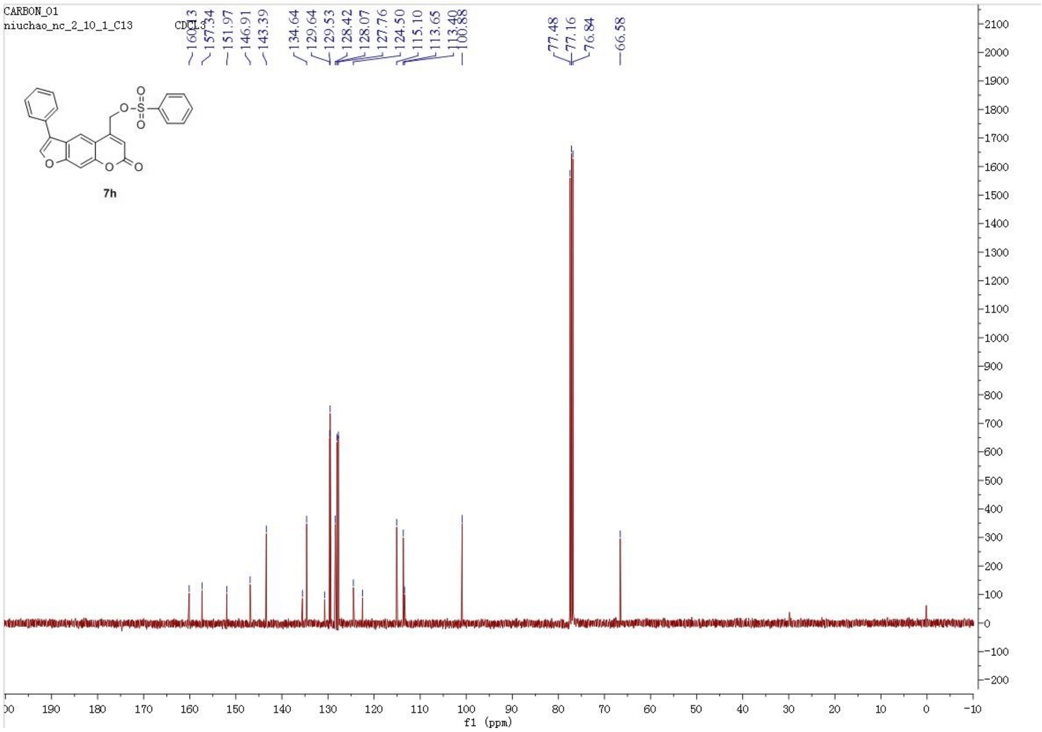

^1H NMR and ^{13}C NMR spectra of **7i**

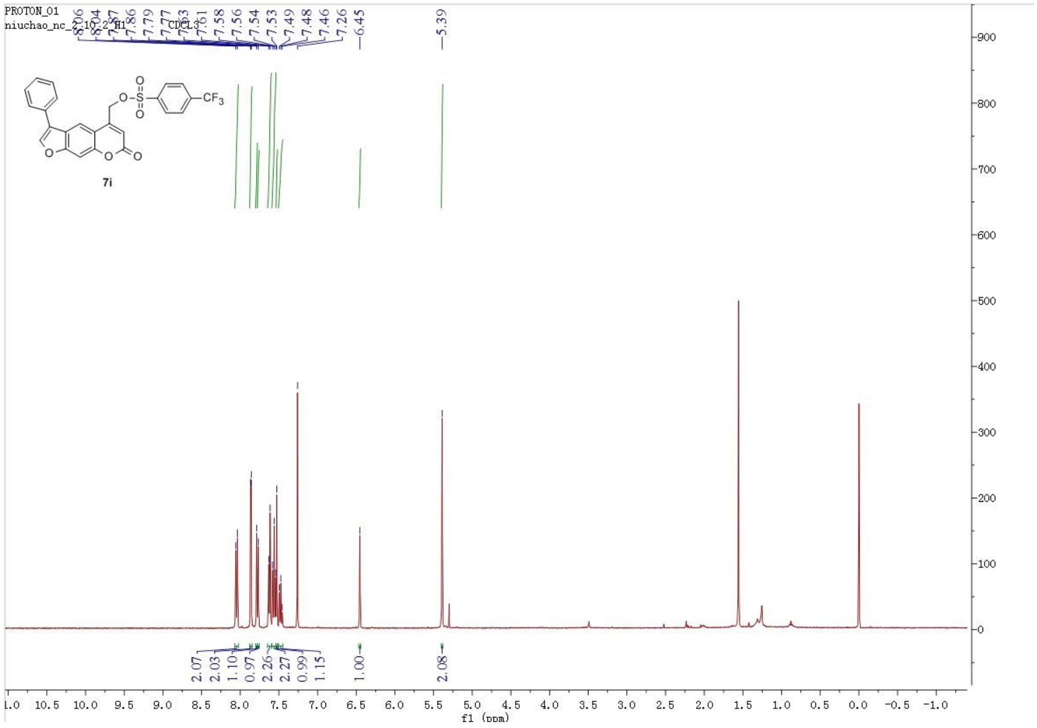

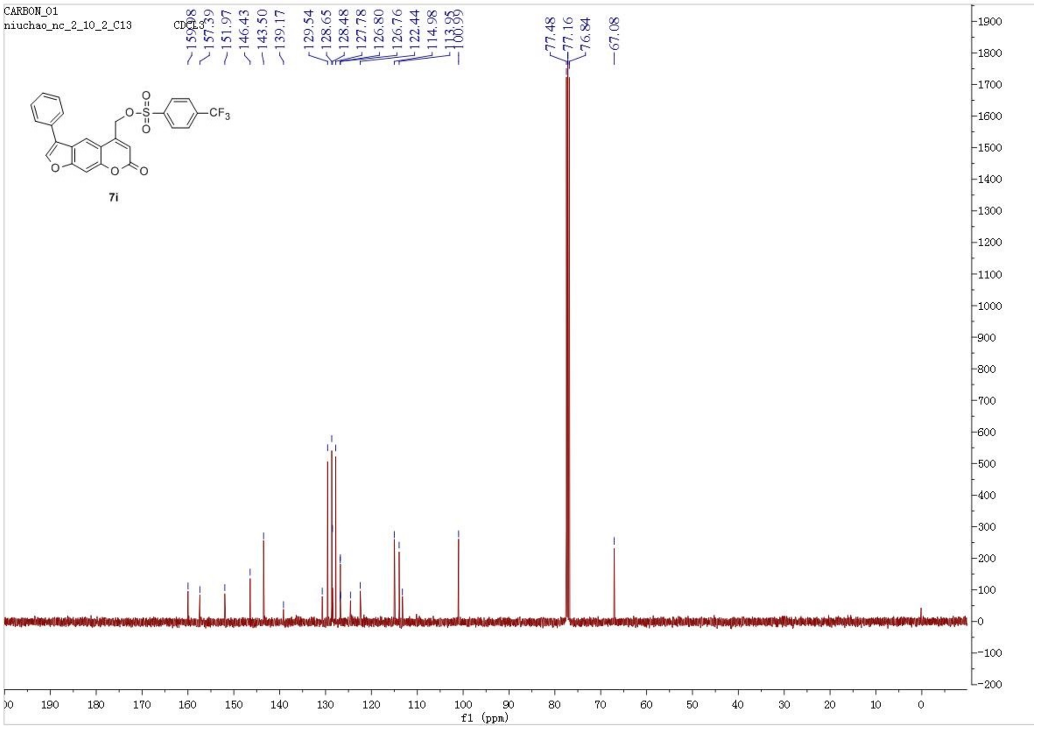

^{1}H NMR and ^{13}C NMR spectra of **7j**

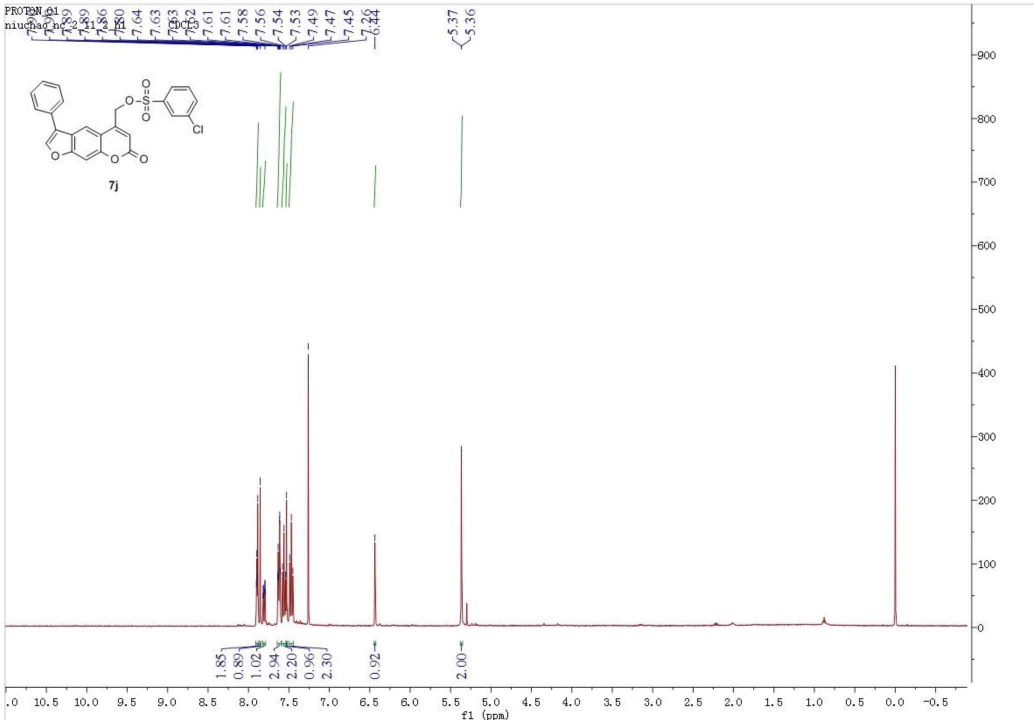

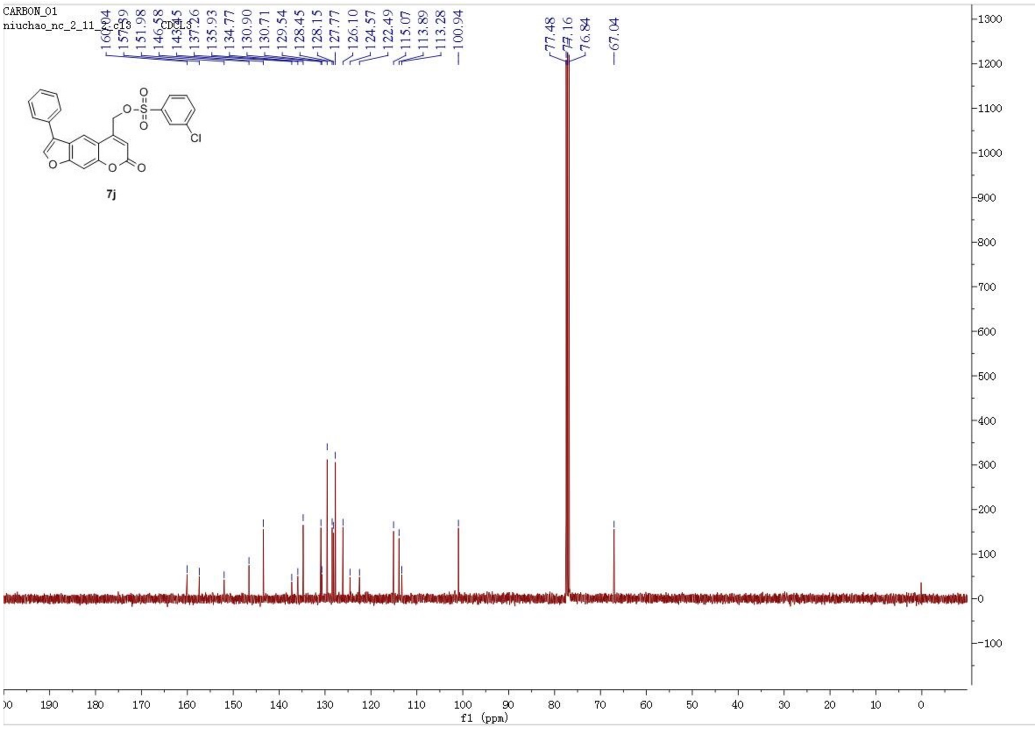

^{1}H NMR and ^{13}C NMR spectra of **7k**

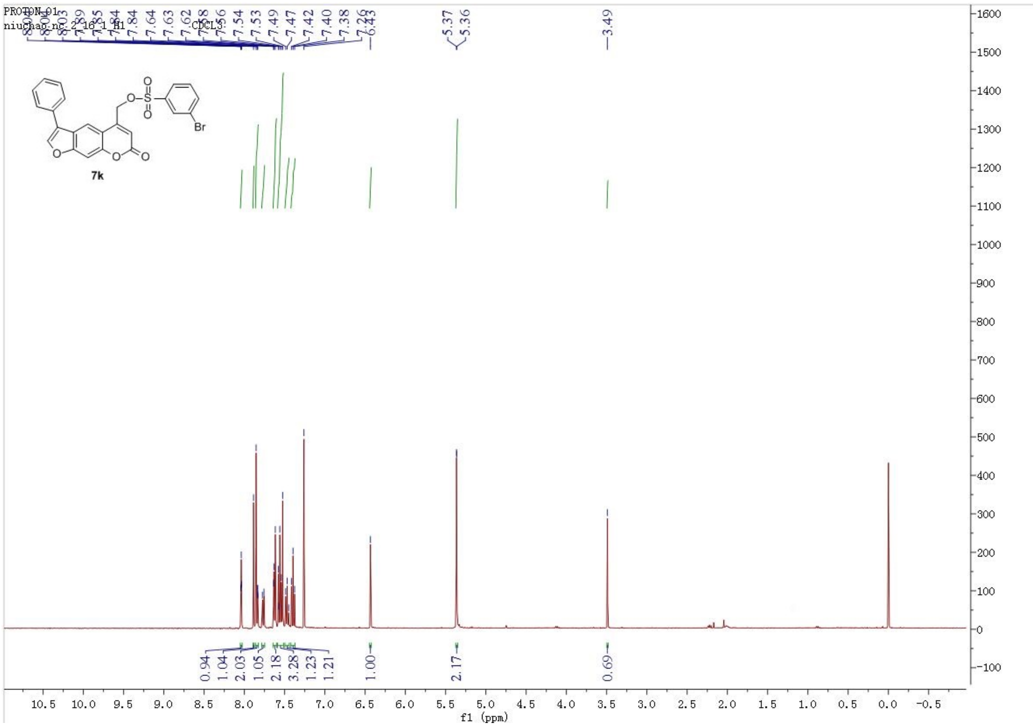

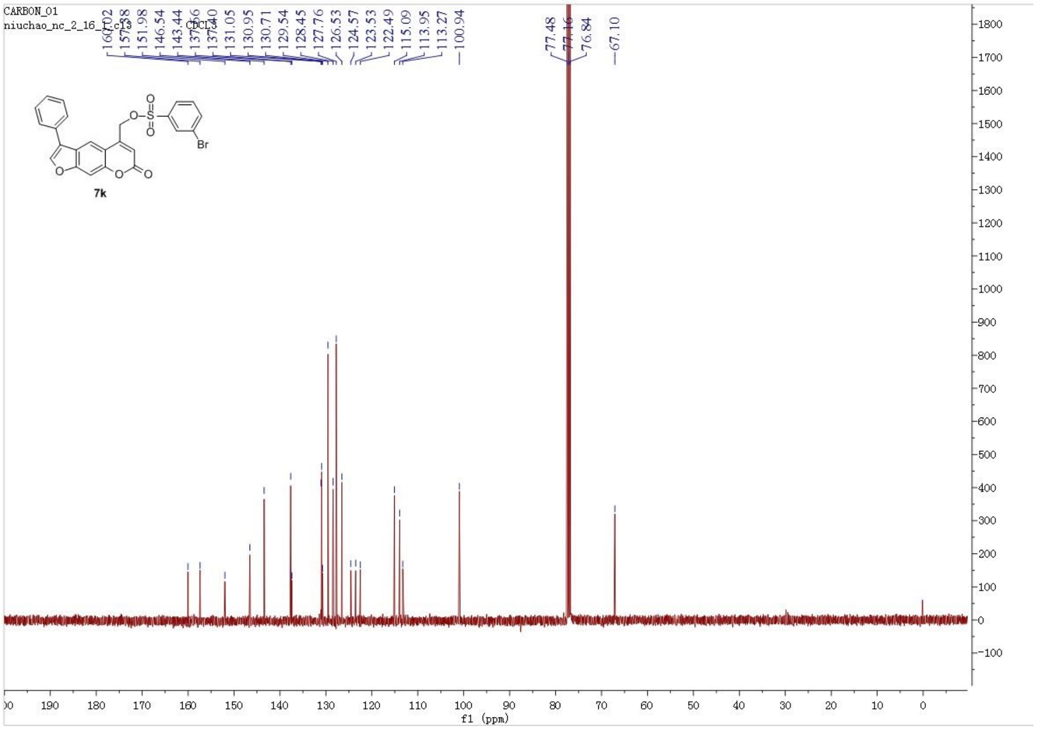

^{1}H NMR and ^{13}C NMR spectra of **7l**

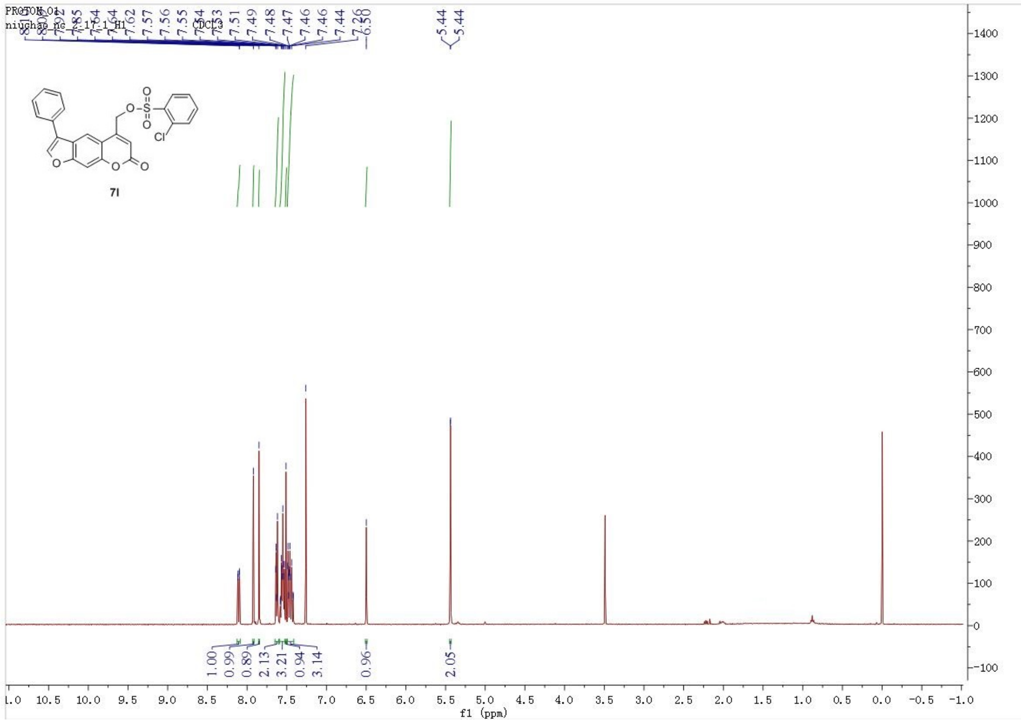

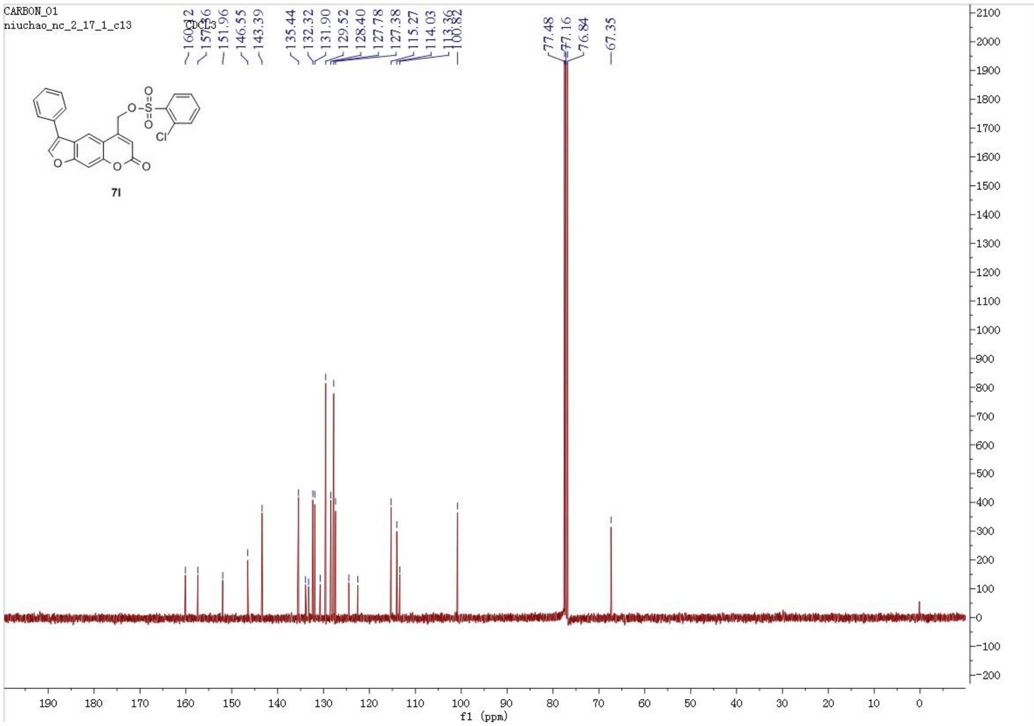

^{1}H NMR and ^{13}C NMR spectra of **7m**

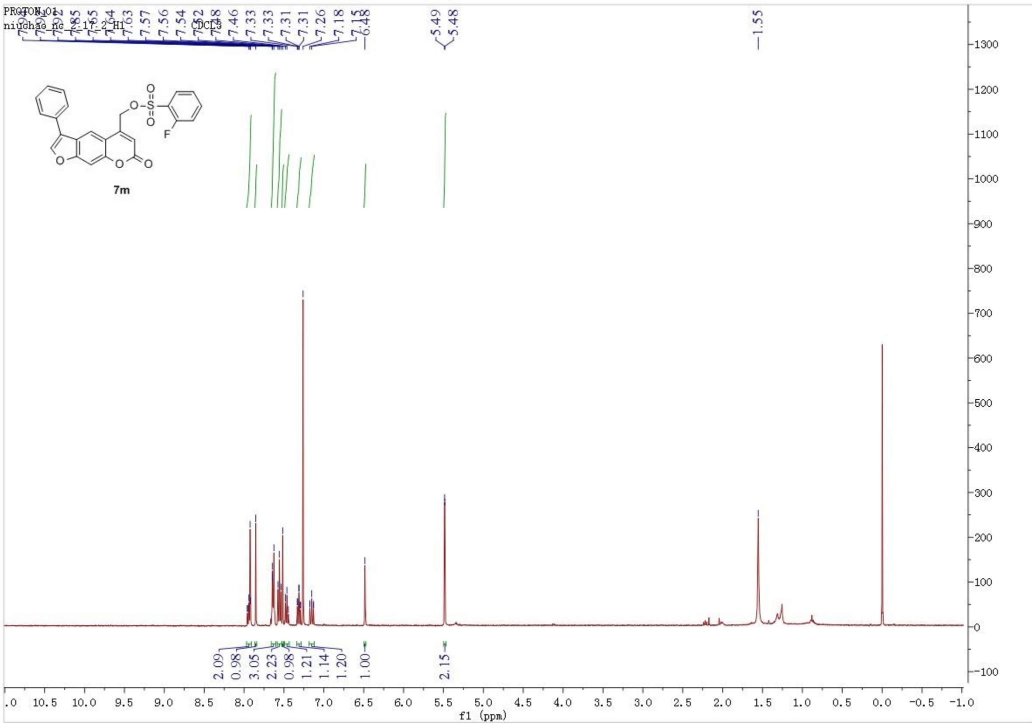

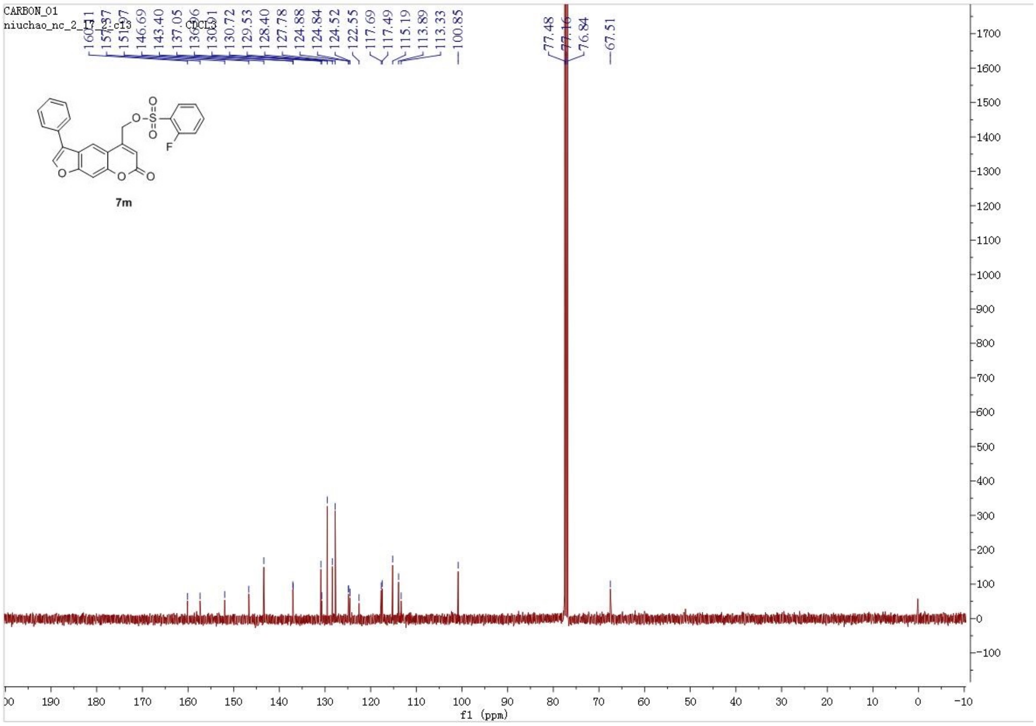

^{1}H NMR and ^{13}C NMR spectra of **7n**

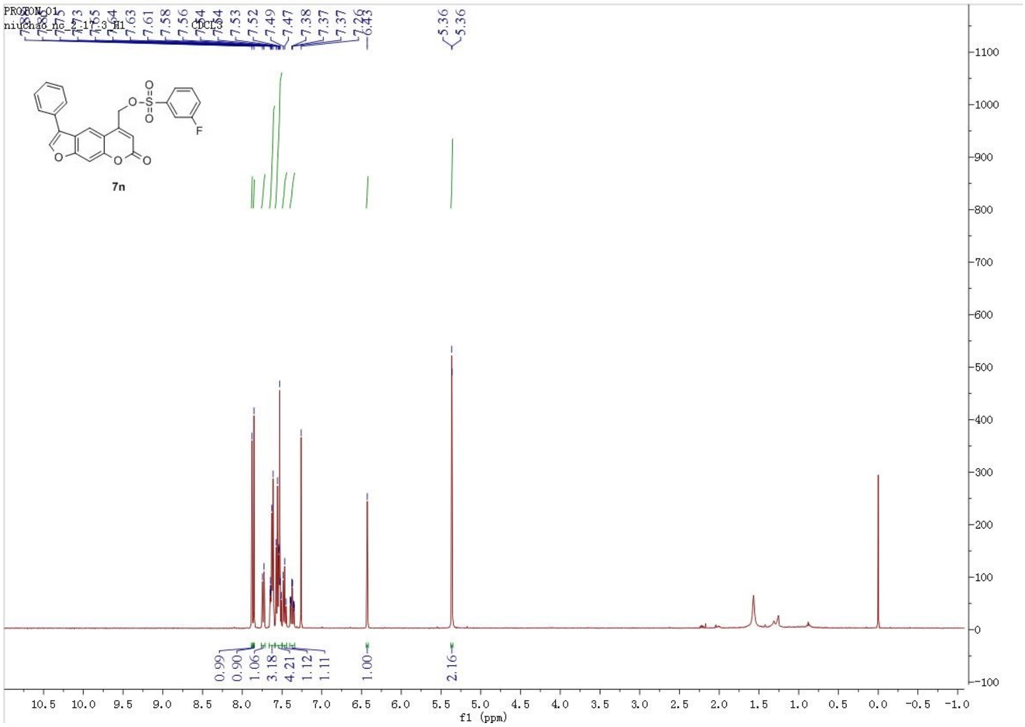

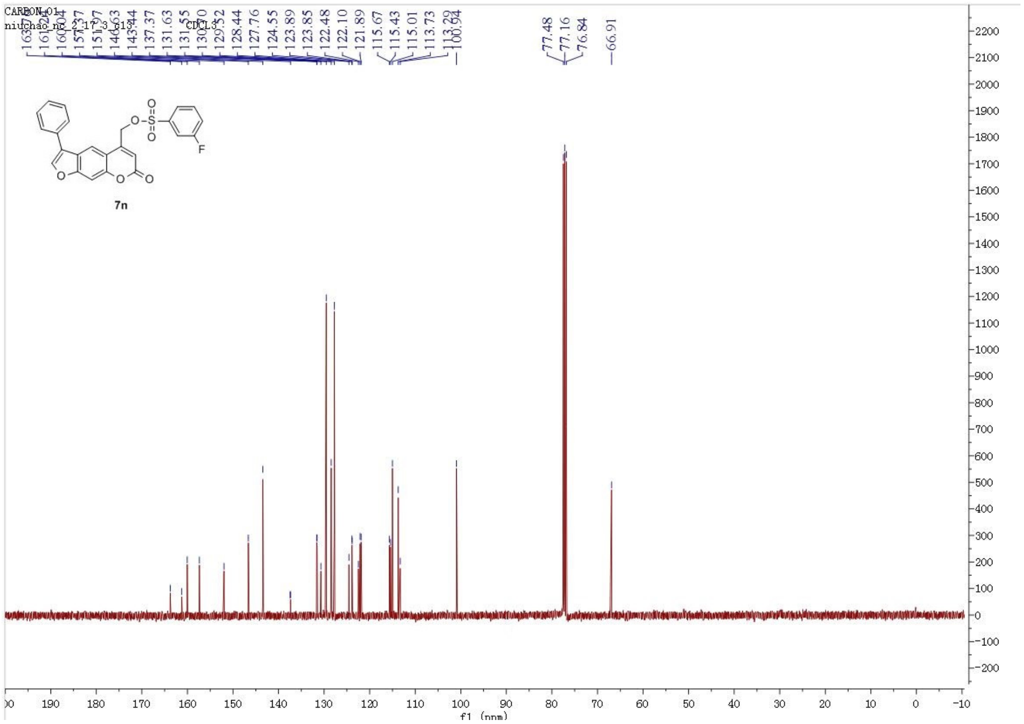

^{1}H NMR and ^{13}C NMR spectra of **7o**

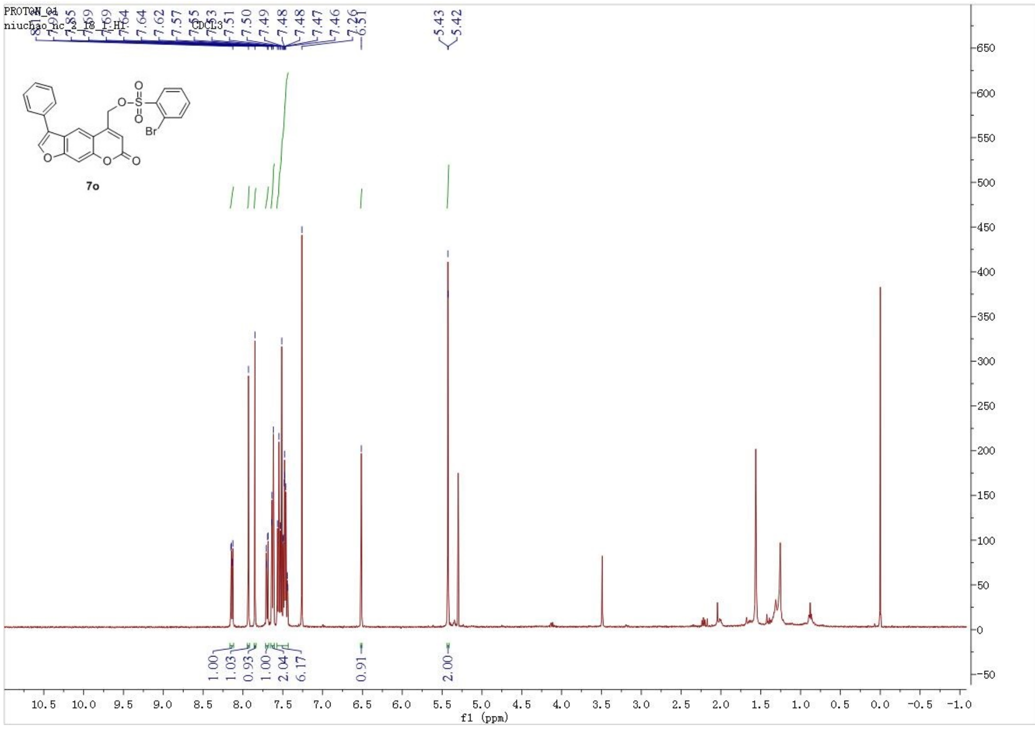

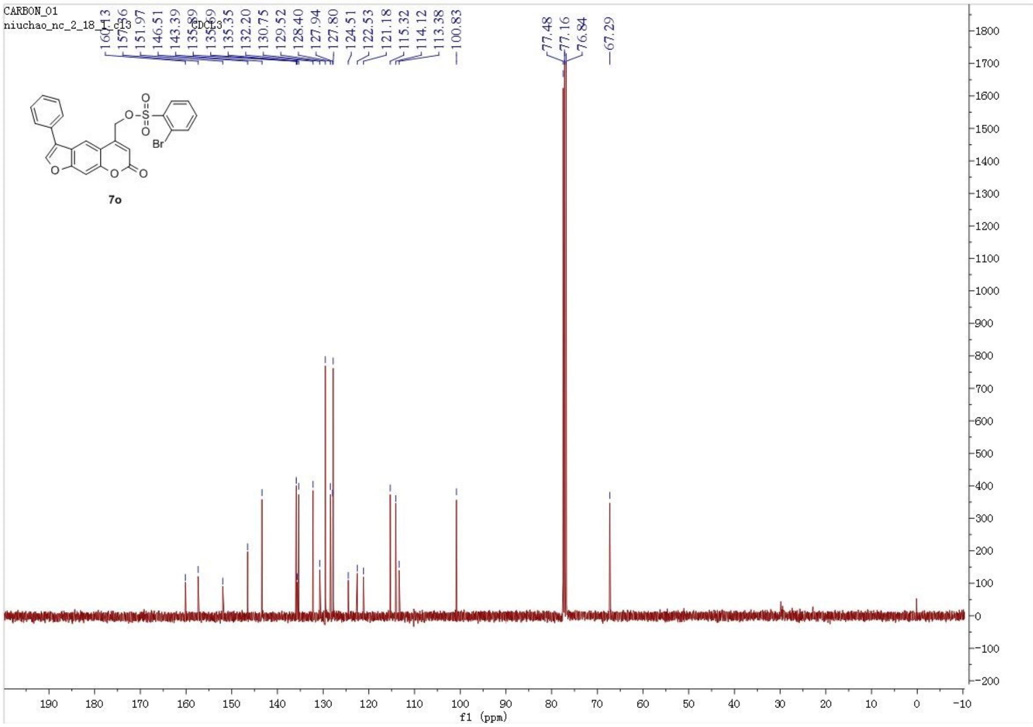

^{1}H NMR and ^{13}C NMR spectra of **7p**

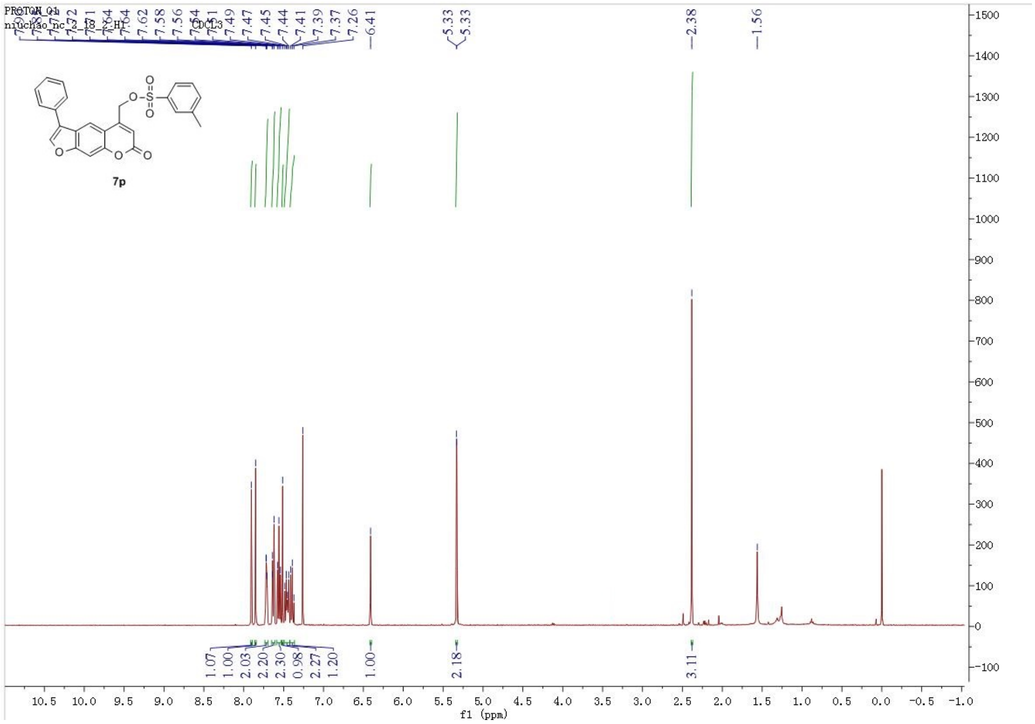

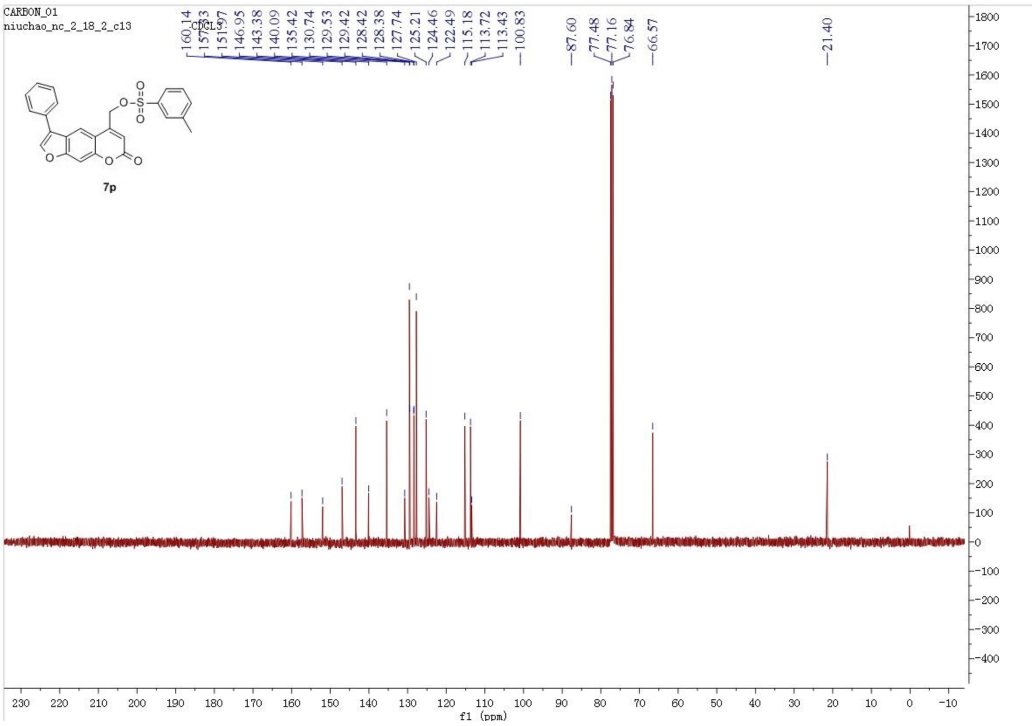

^{1}H NMR and ^{13}C NMR spectra of **7q**

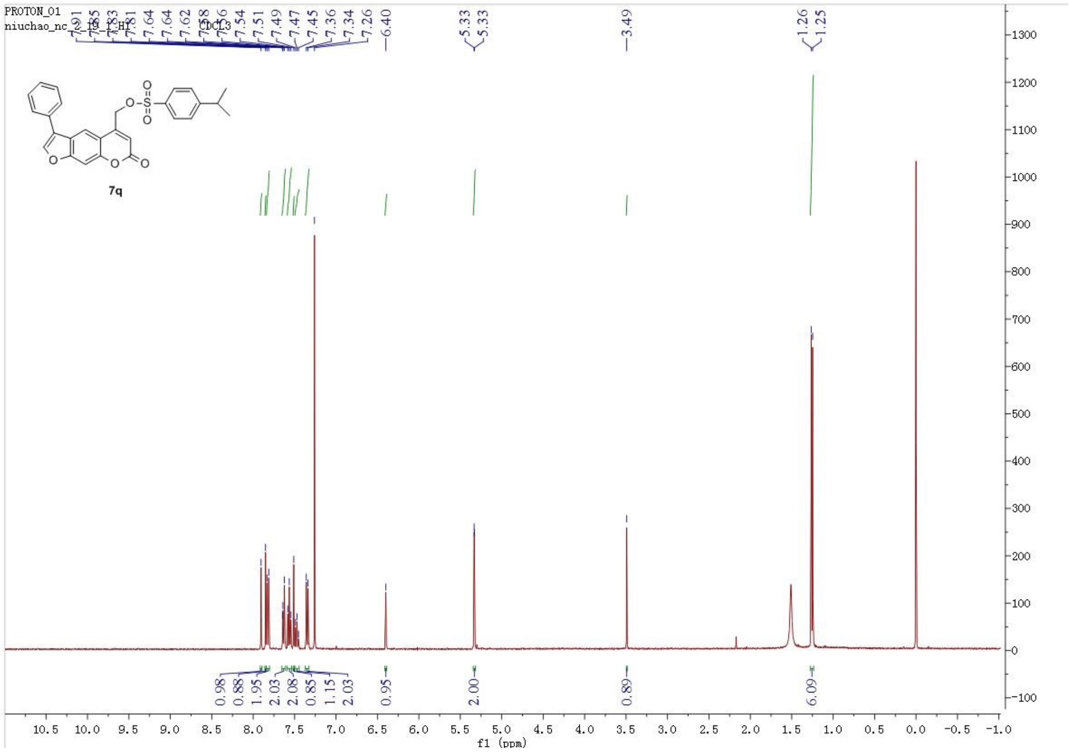

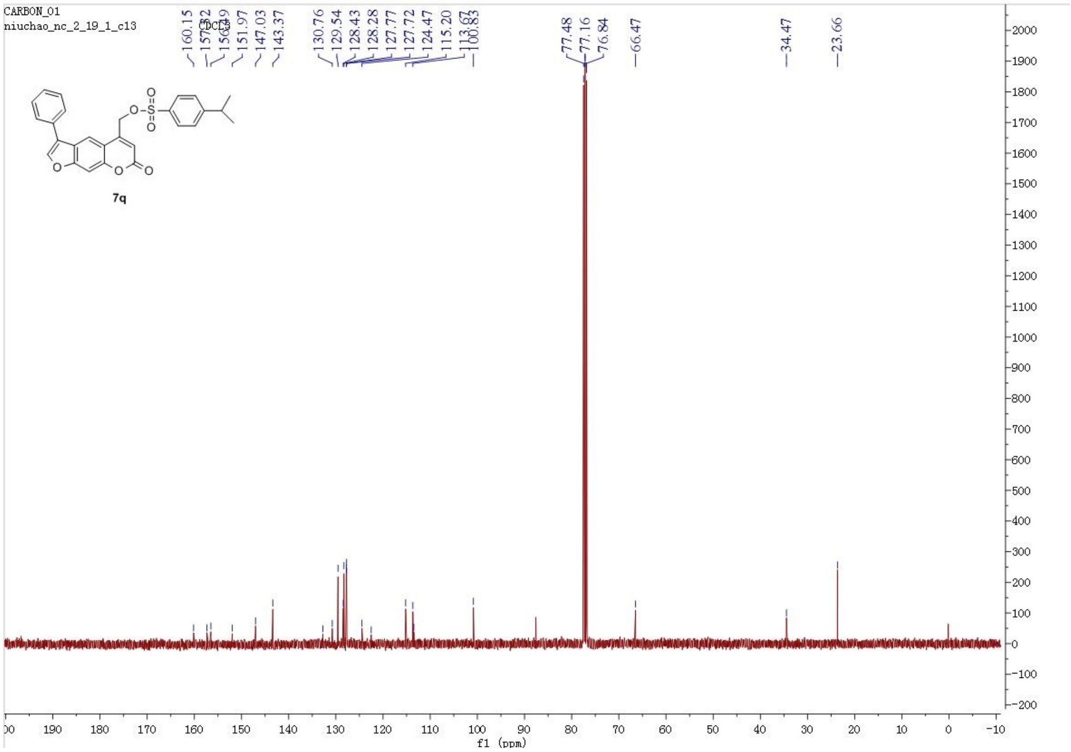

^{1}H NMR and ^{13}C NMR spectra of **7r**

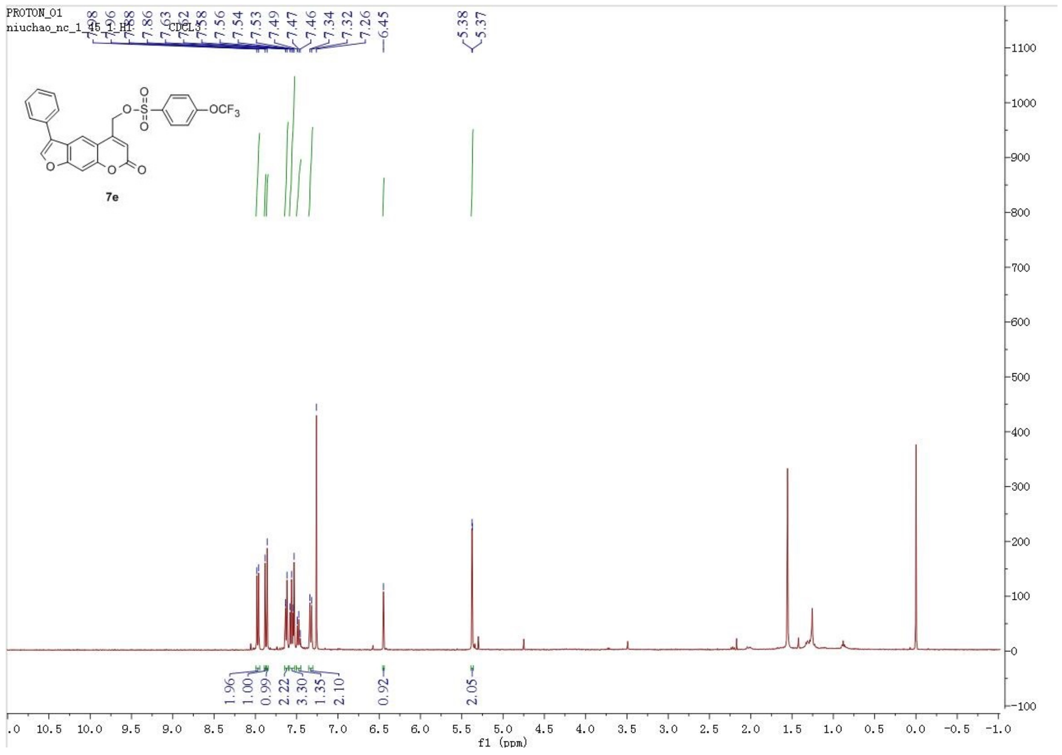

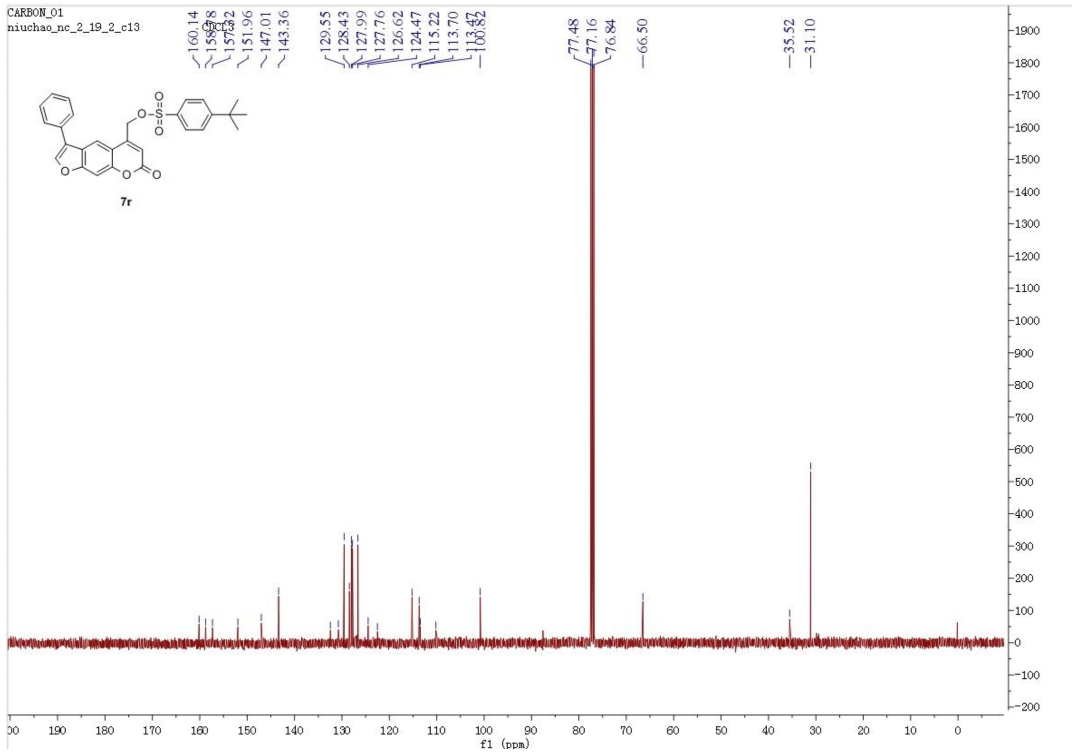

^{1}H NMR and ^{13}C NMR spectra of **7s**

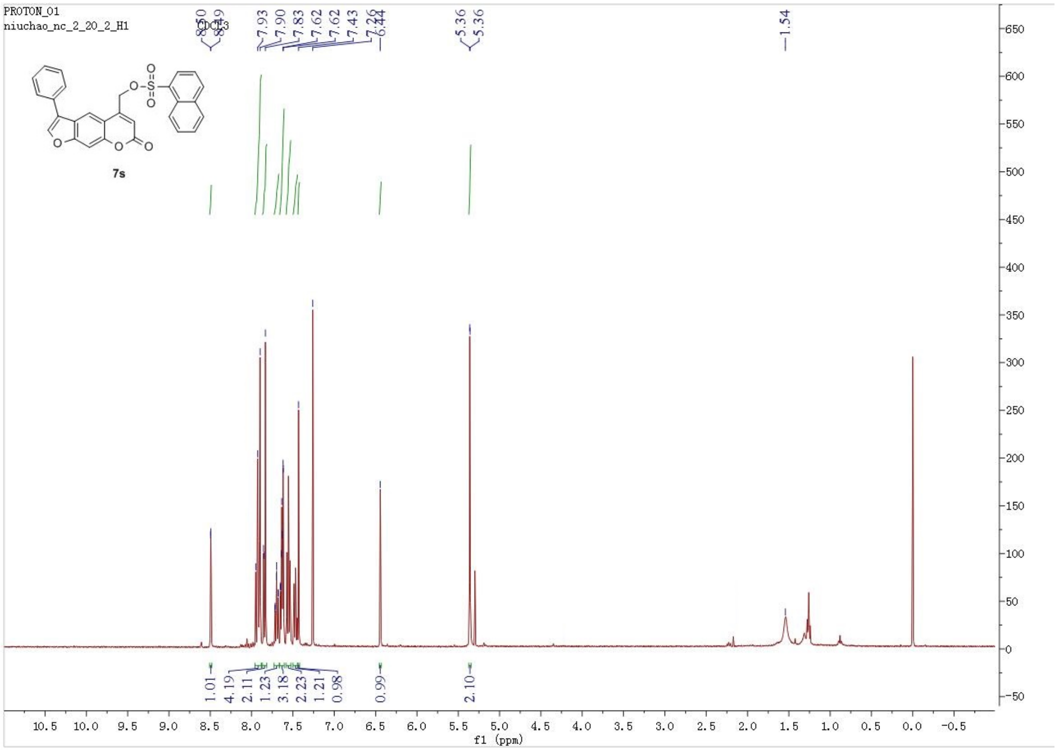

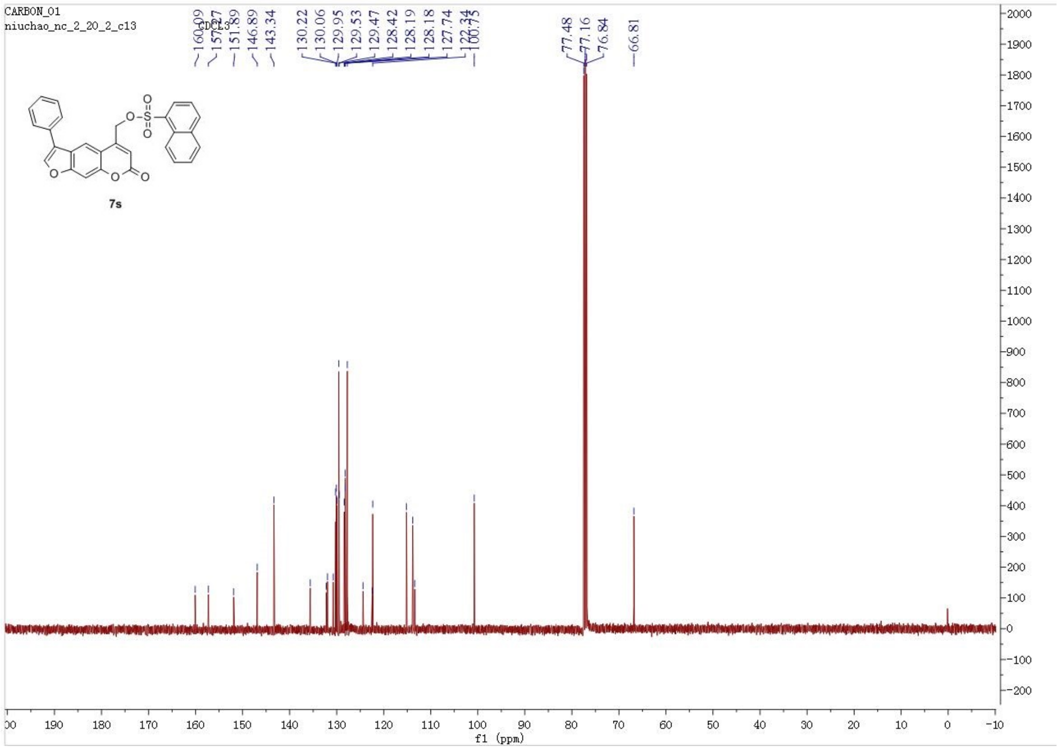

^{1}H NMR and ^{13}C NMR spectra of **7t**

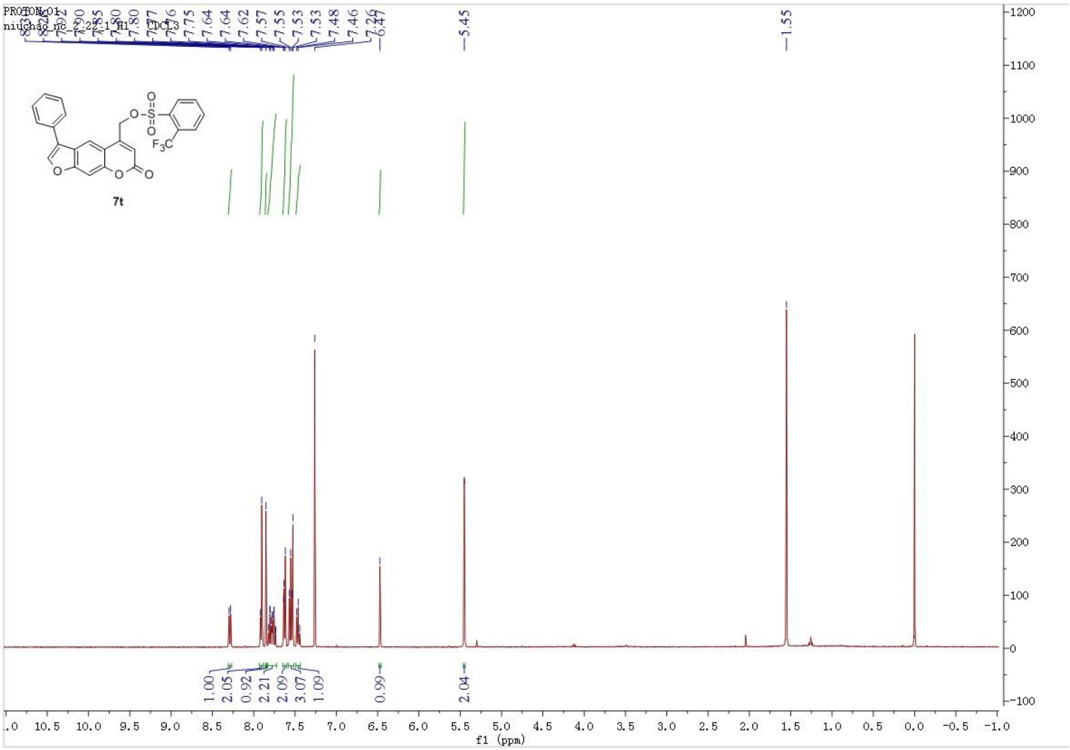

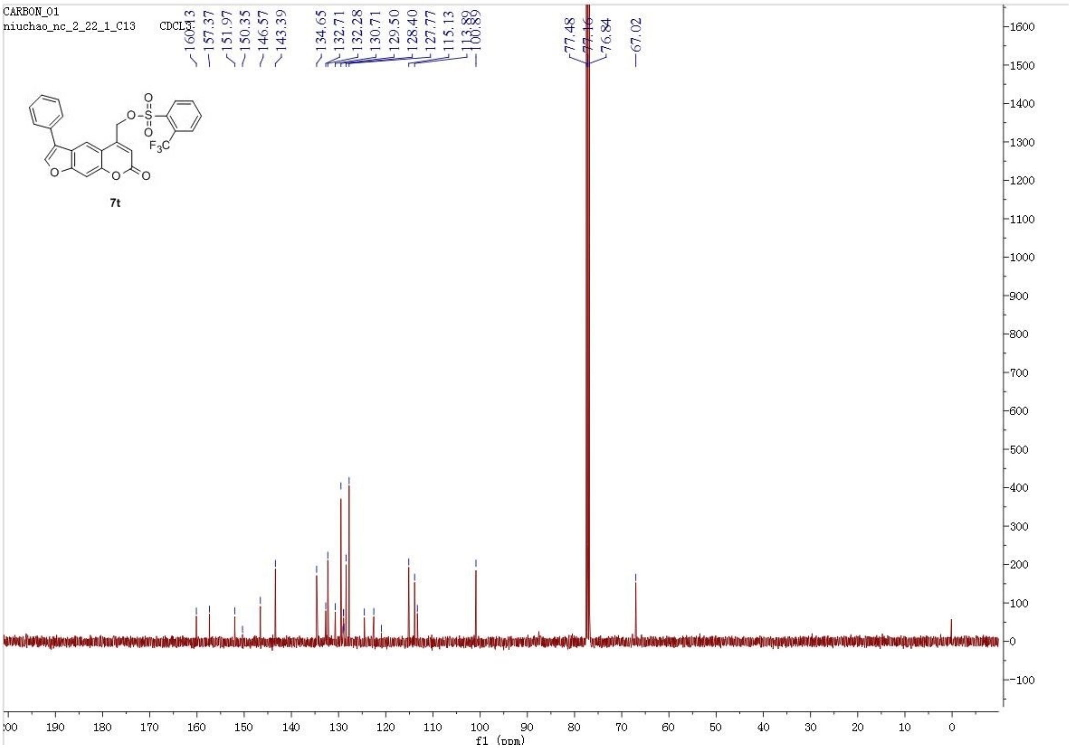

^{1}H NMR and ^{13}C NMR spectra of **7u**

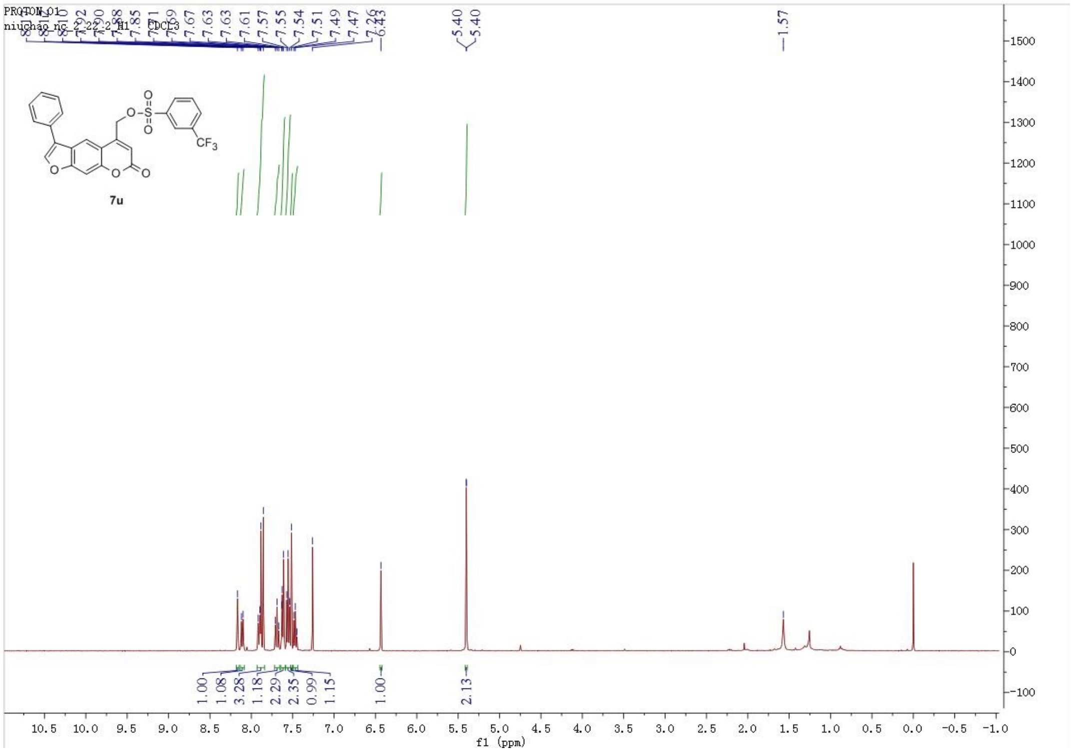

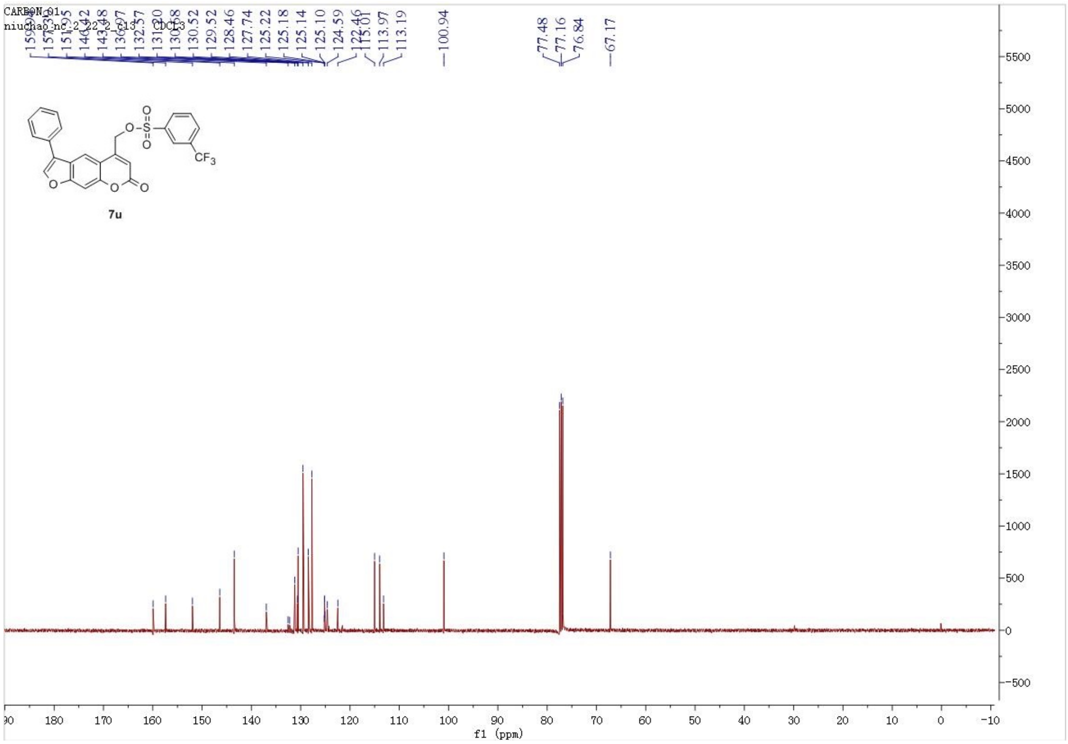

^{1}H NMR and ^{13}C NMR spectra of **7v**

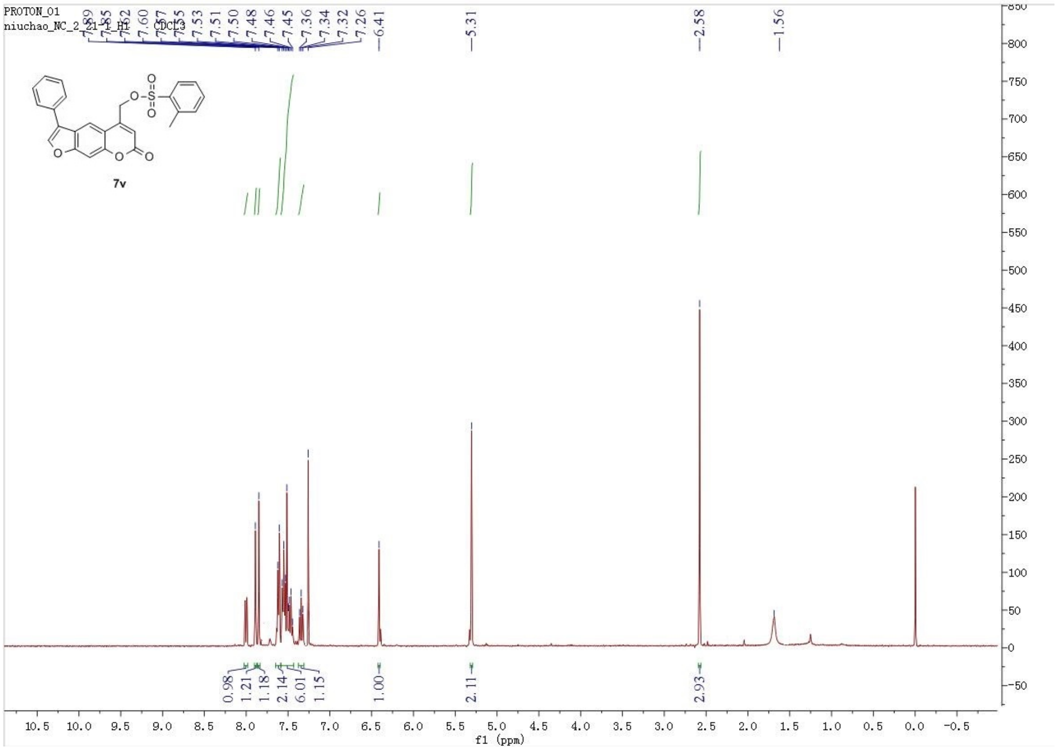

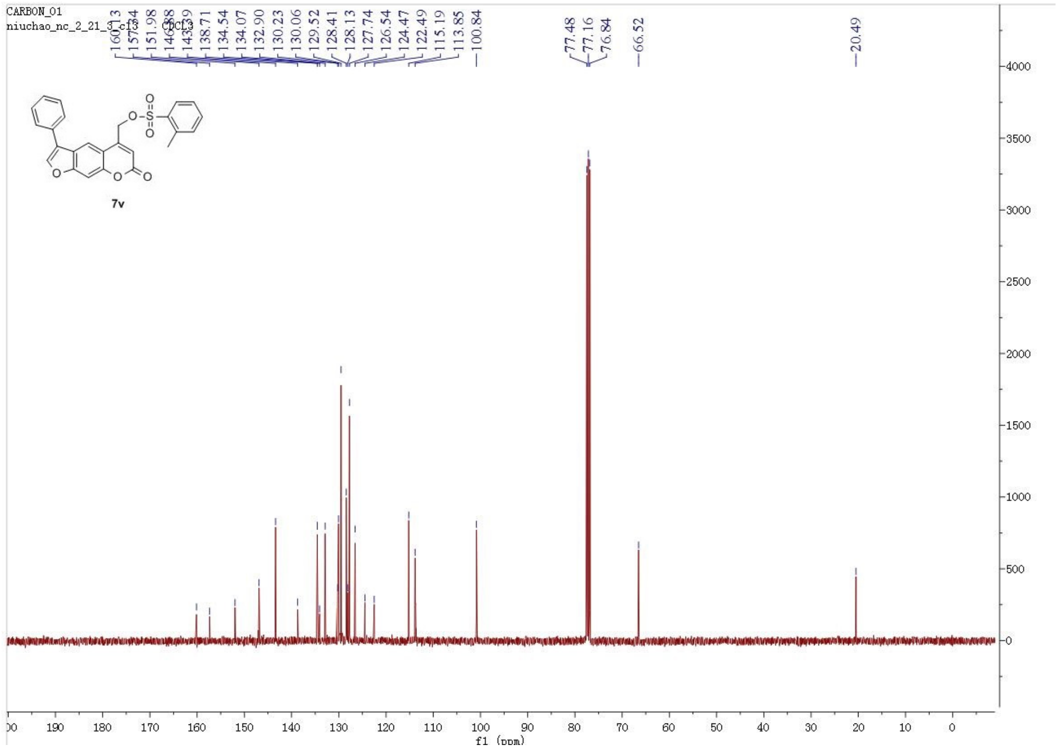

^{1}H NMR and ^{13}C NMR spectra of **7w**

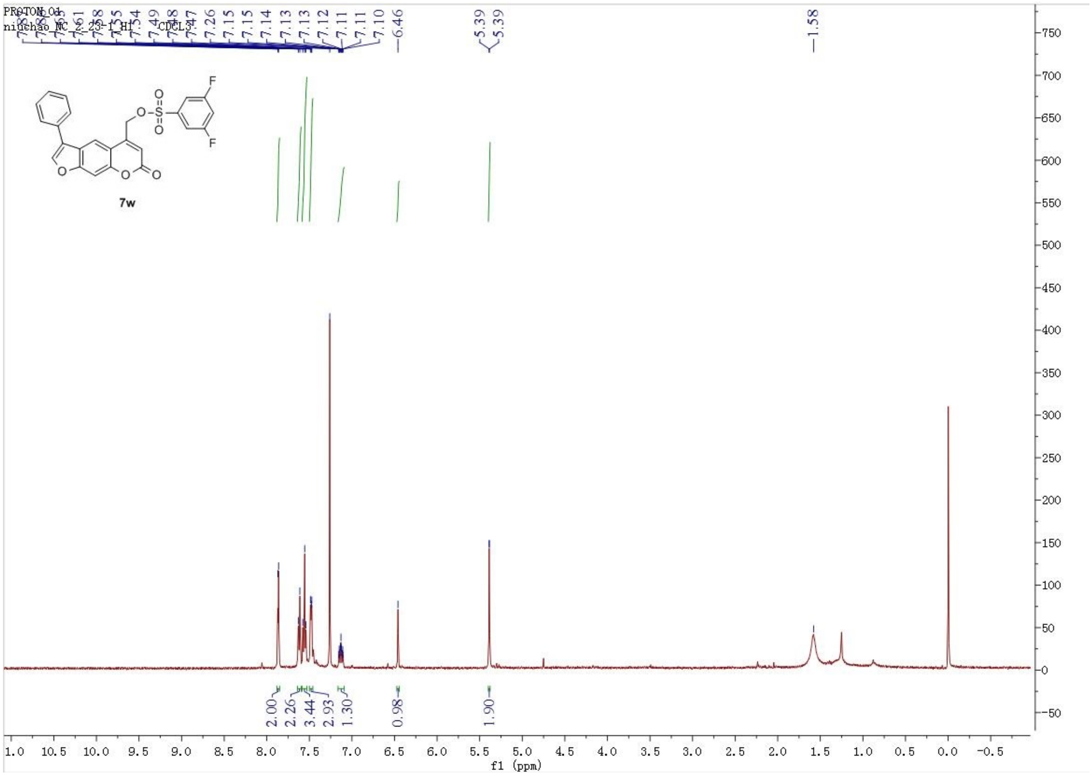

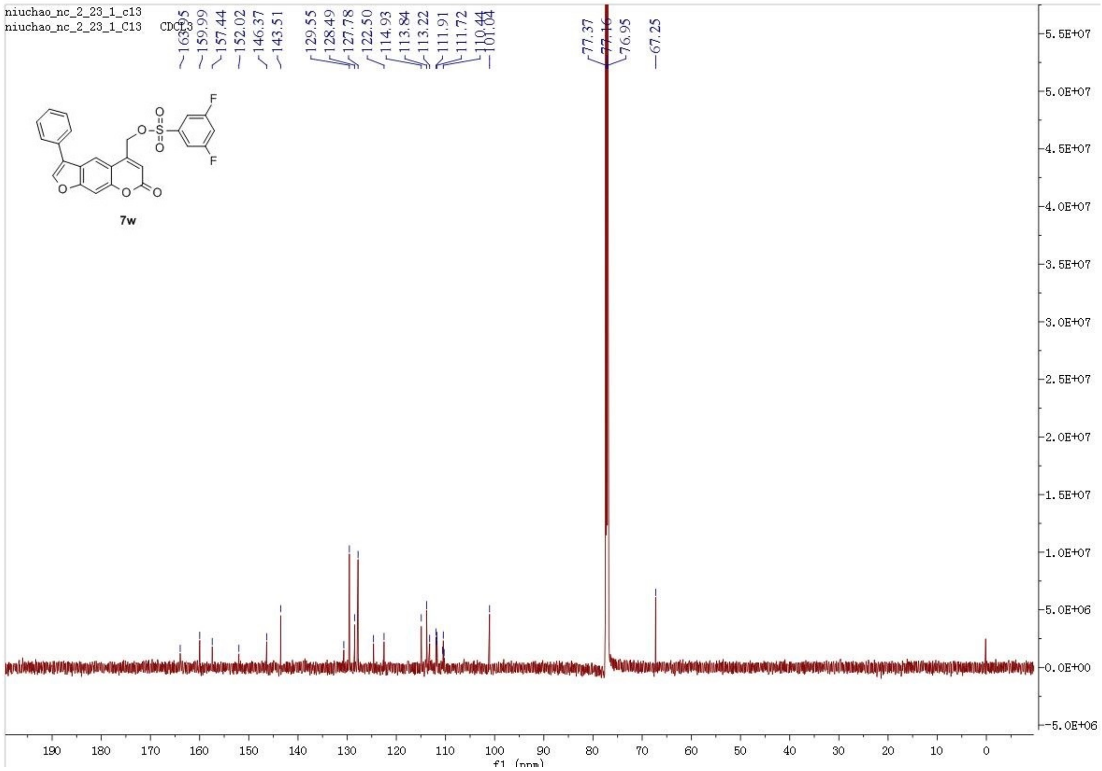

¹H NMR and ¹³C NMR spectra of **7x**

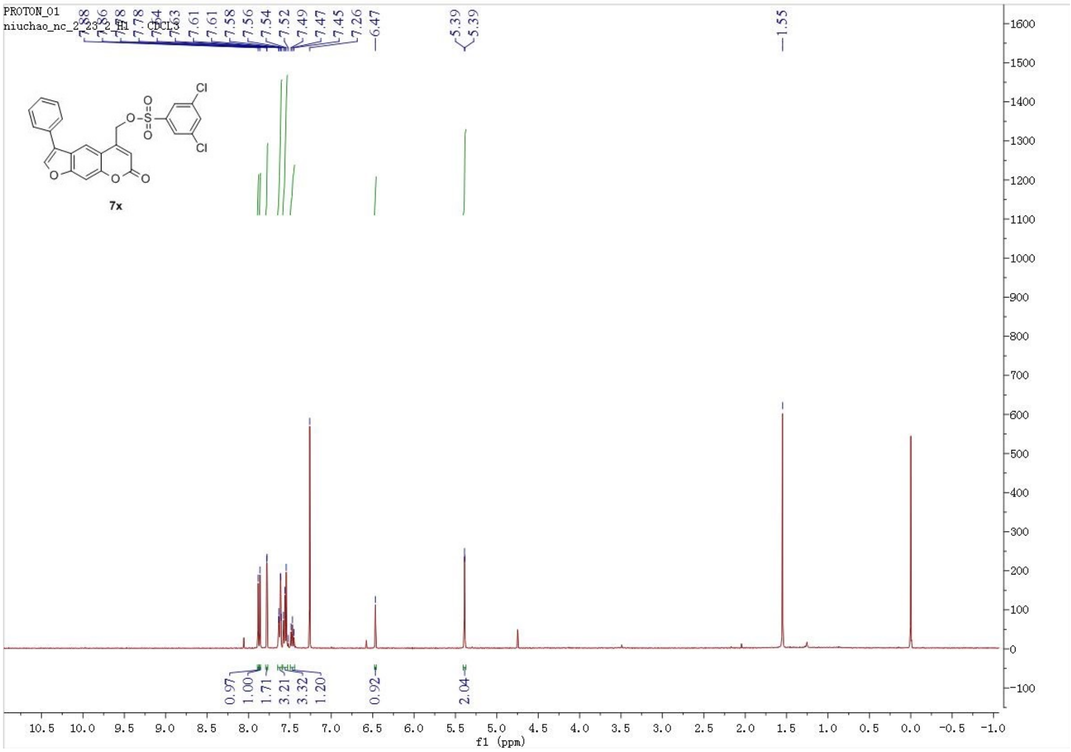

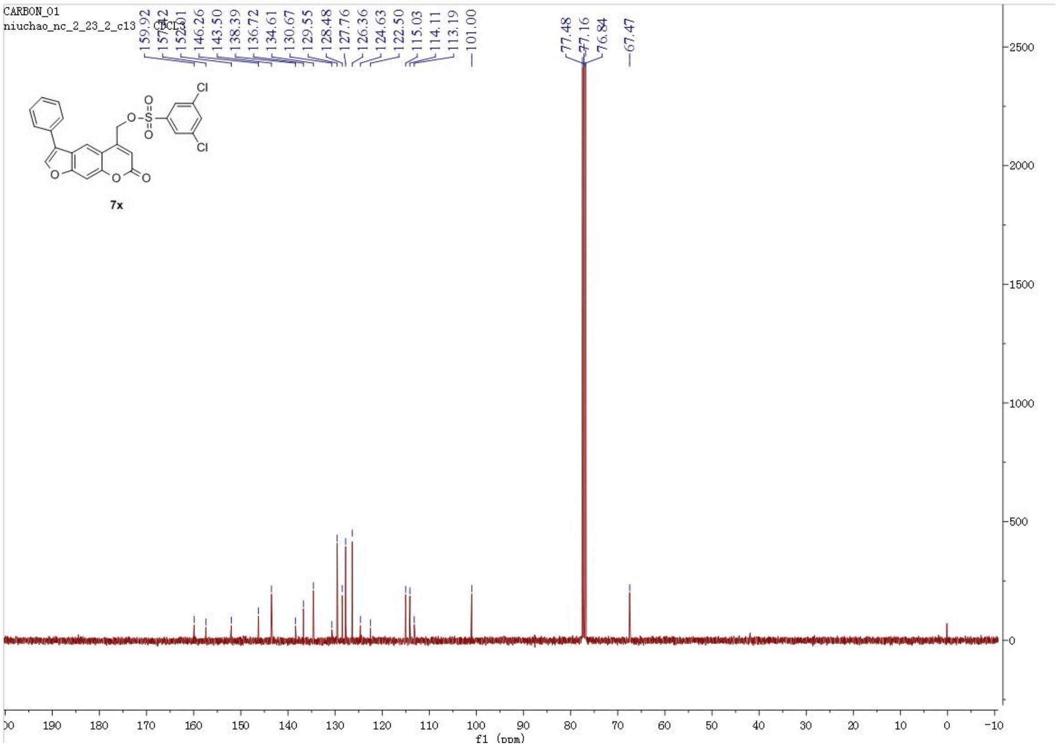

^{1}H NMR and ^{13}C NMR spectra of **7y**

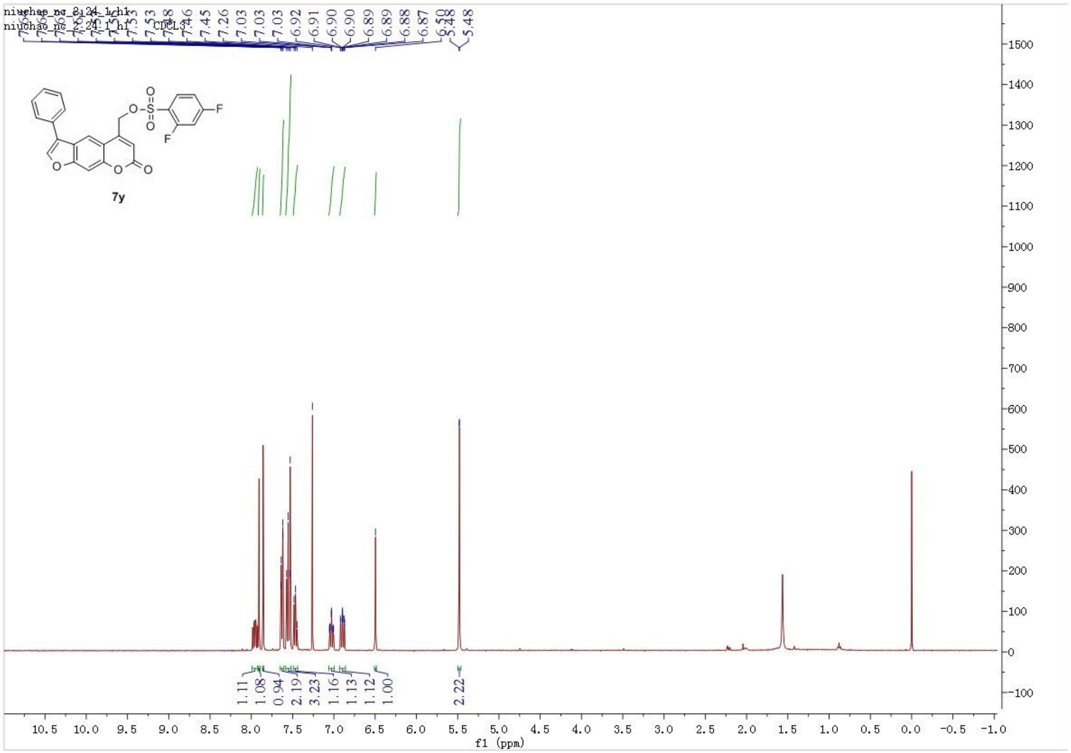

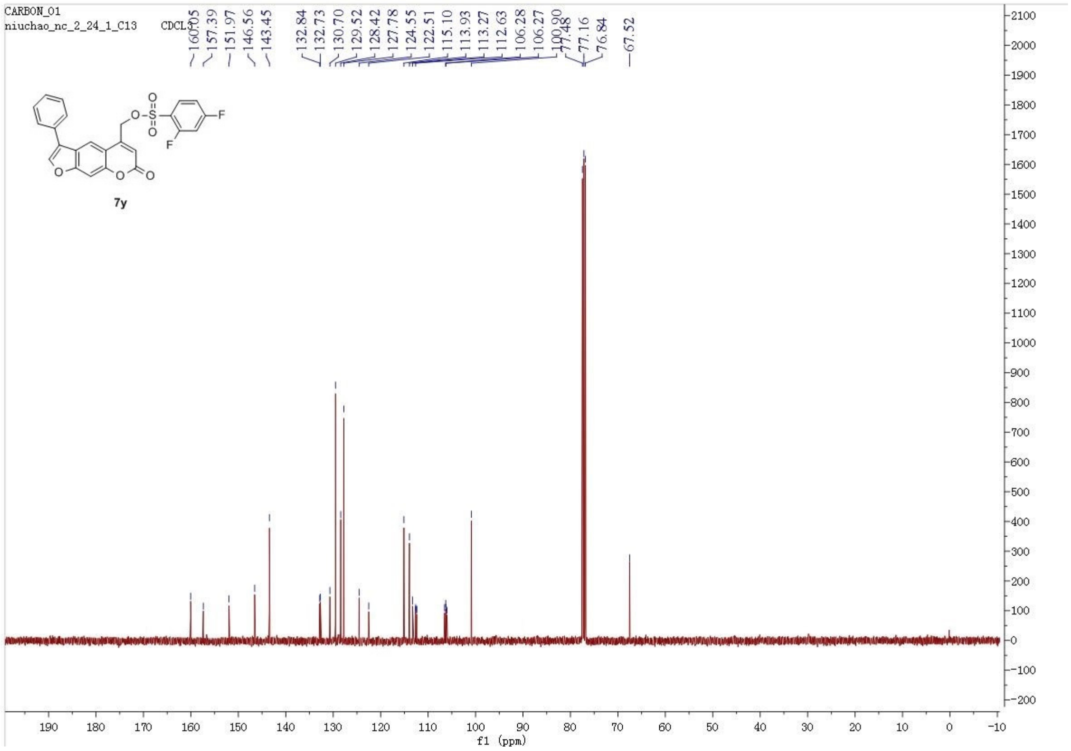

^{1}H NMR and ^{13}C NMR spectra of **7z**

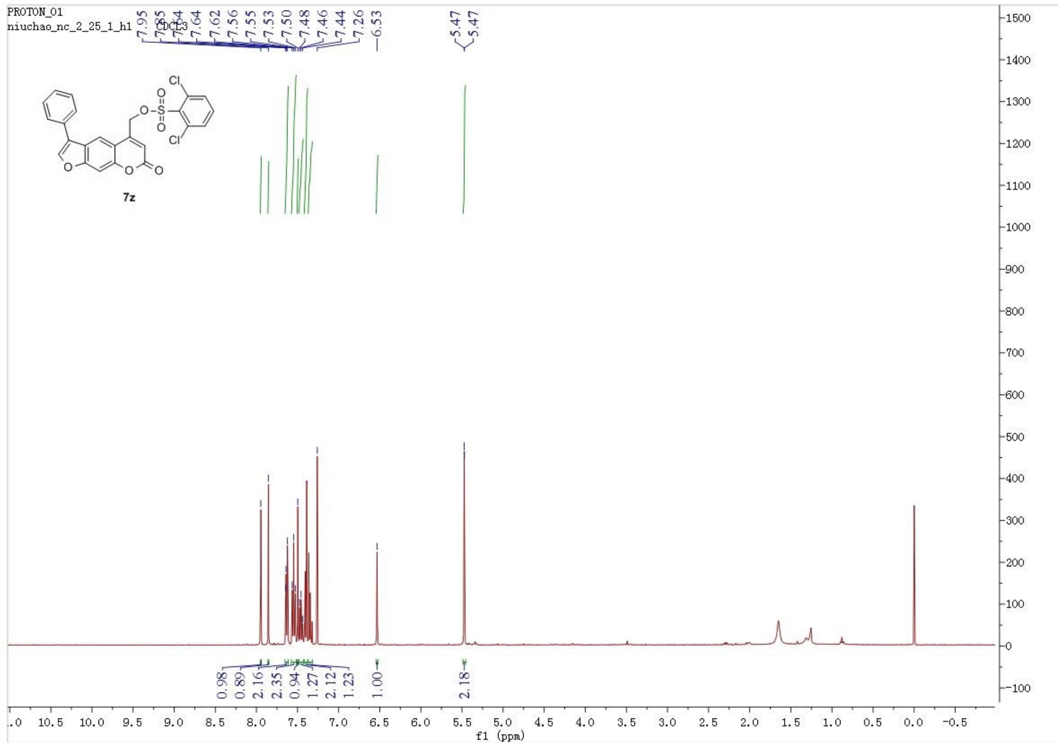

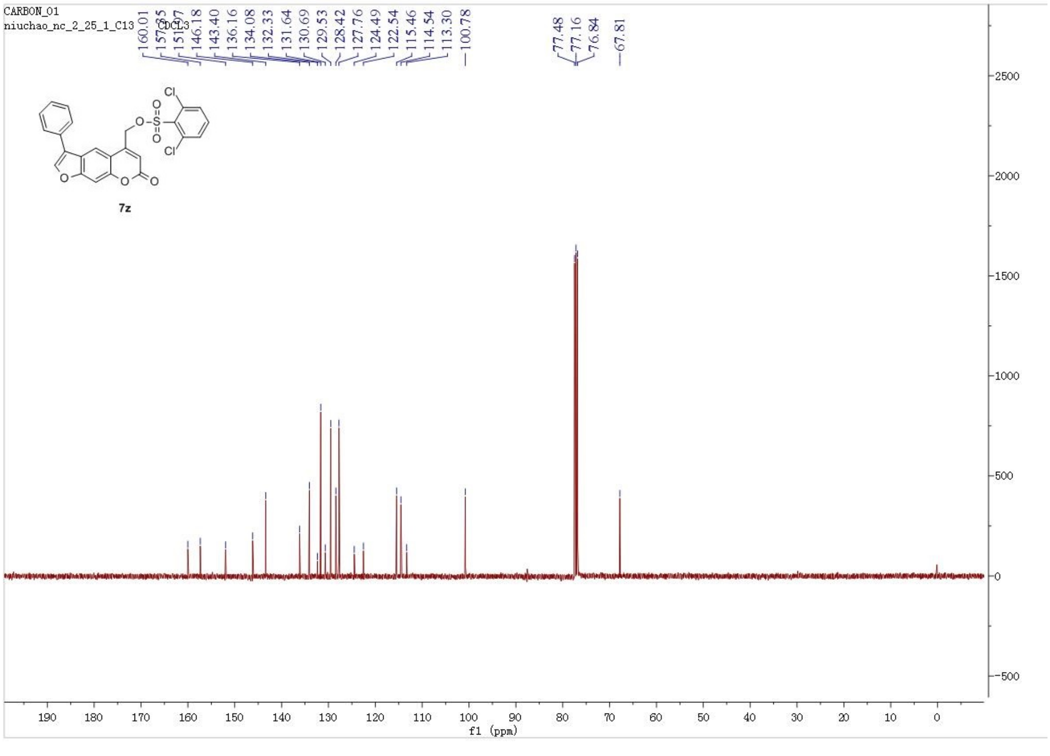

^{1}H NMR and ^{13}C NMR spectra of **7aa**

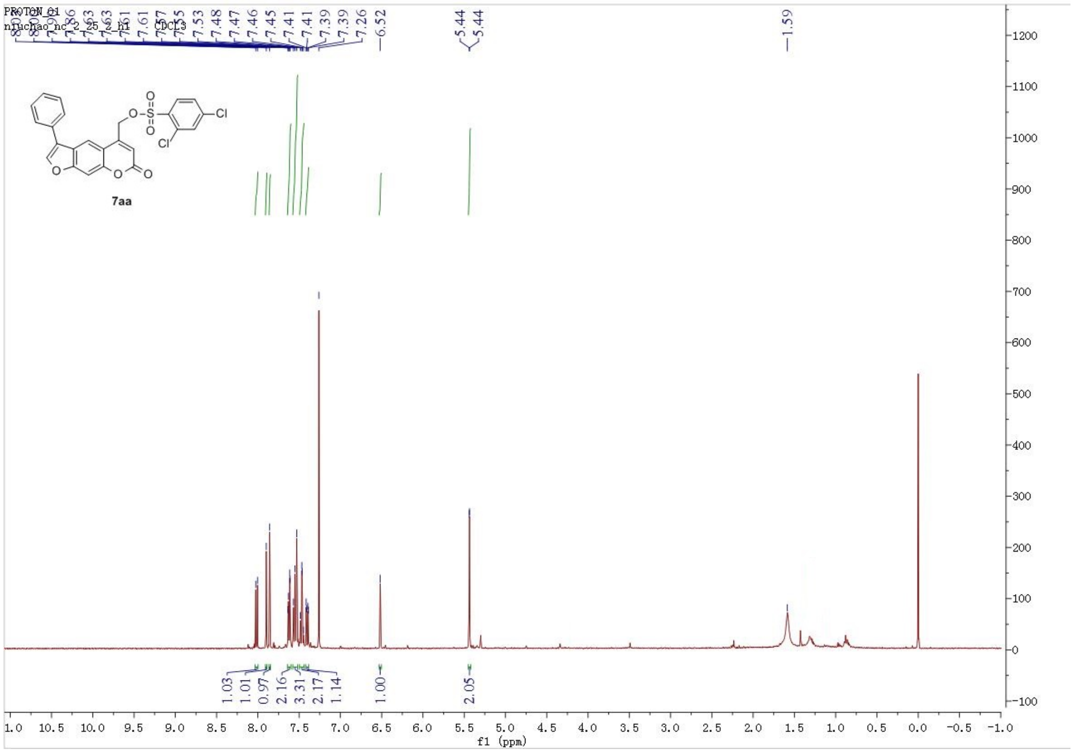

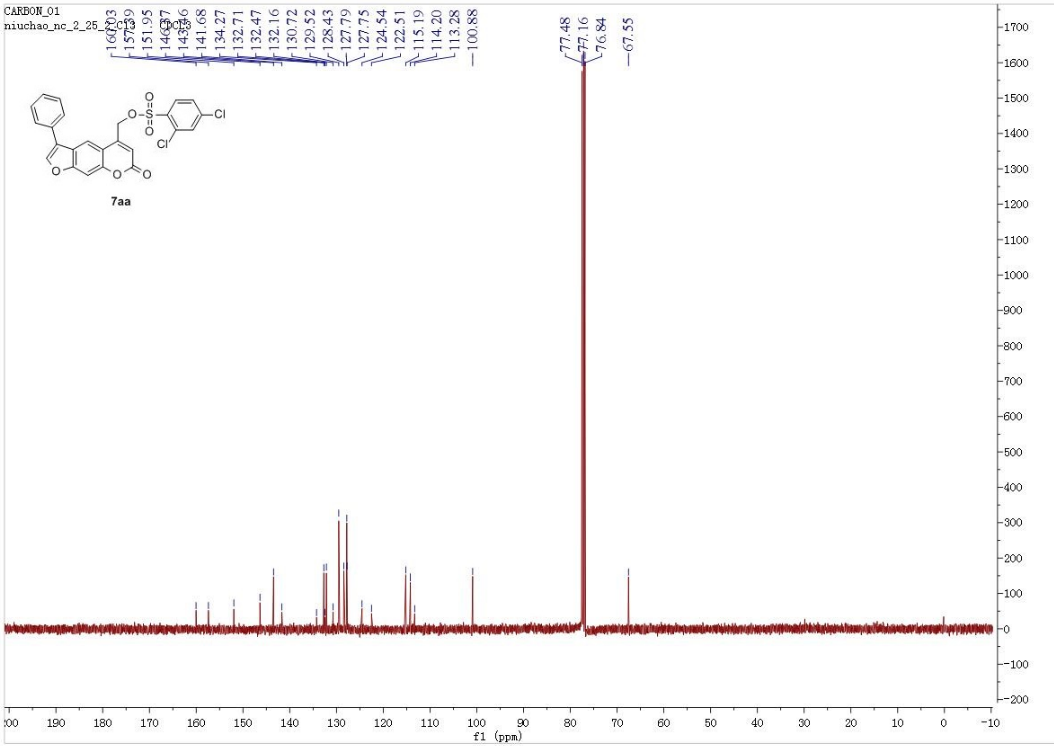

^{1}H NMR and ^{13}C NMR spectra of **7ab**

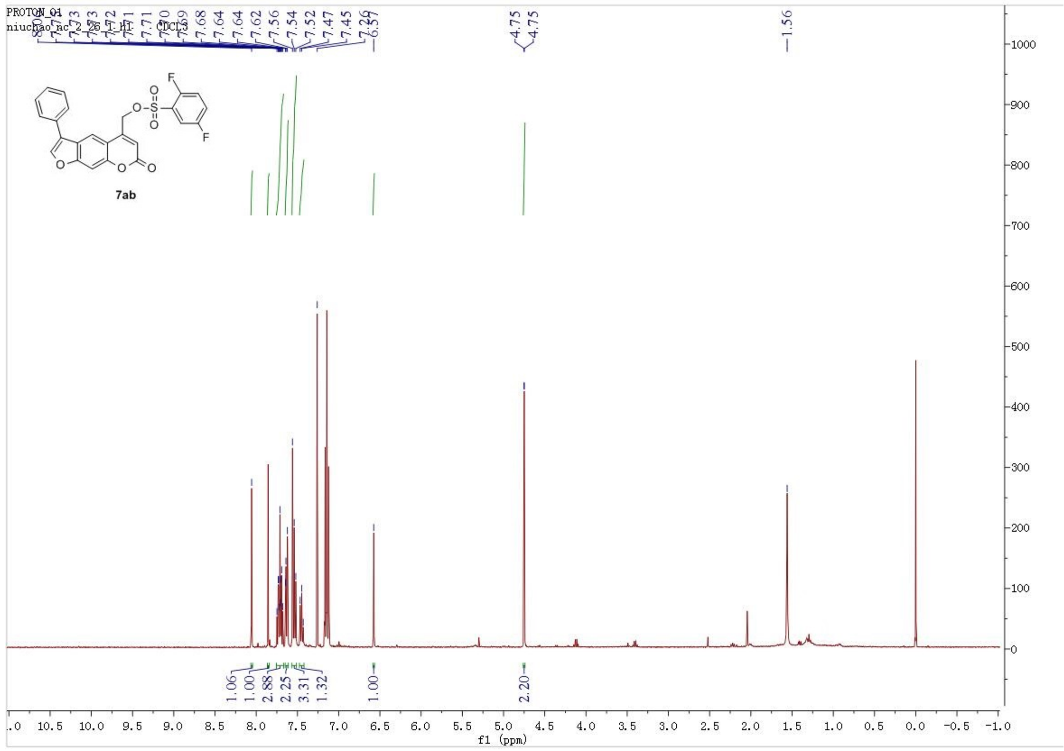

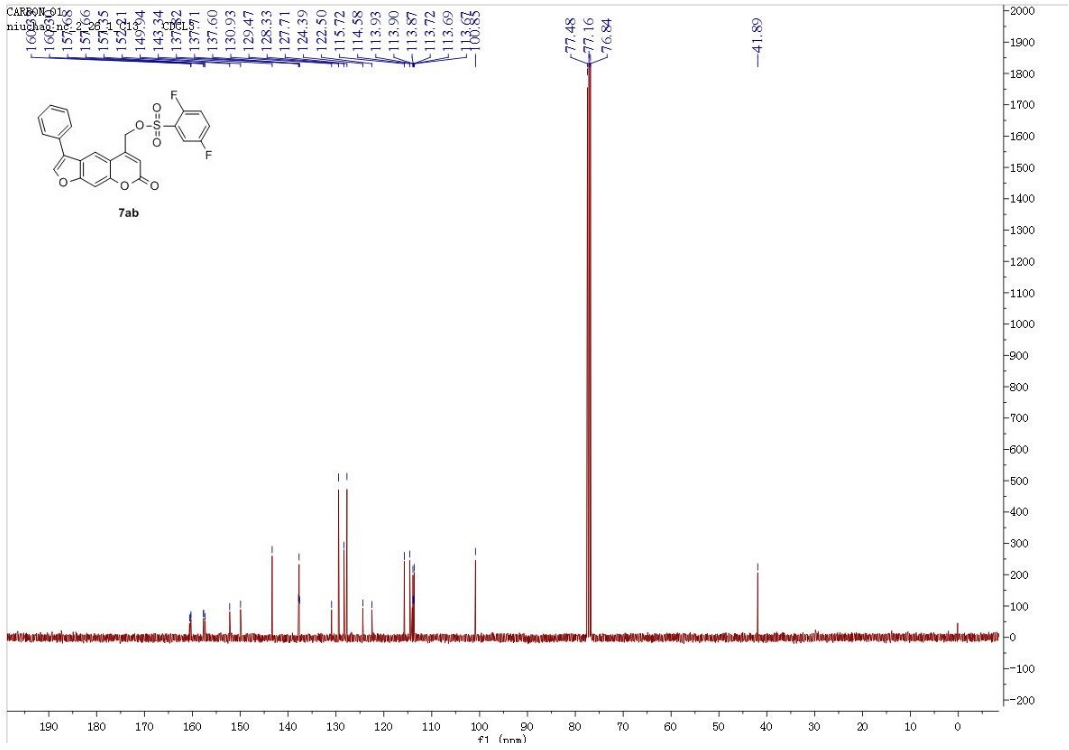

^{1}H NMR and ^{13}C NMR spectra of **7ac**

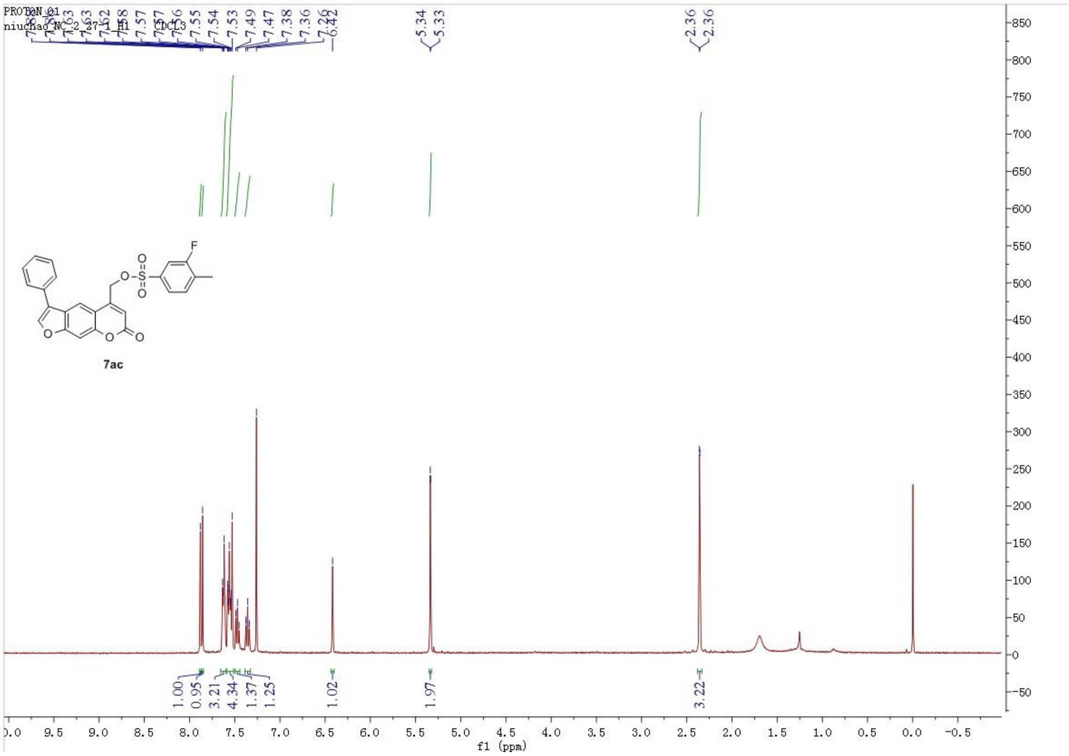

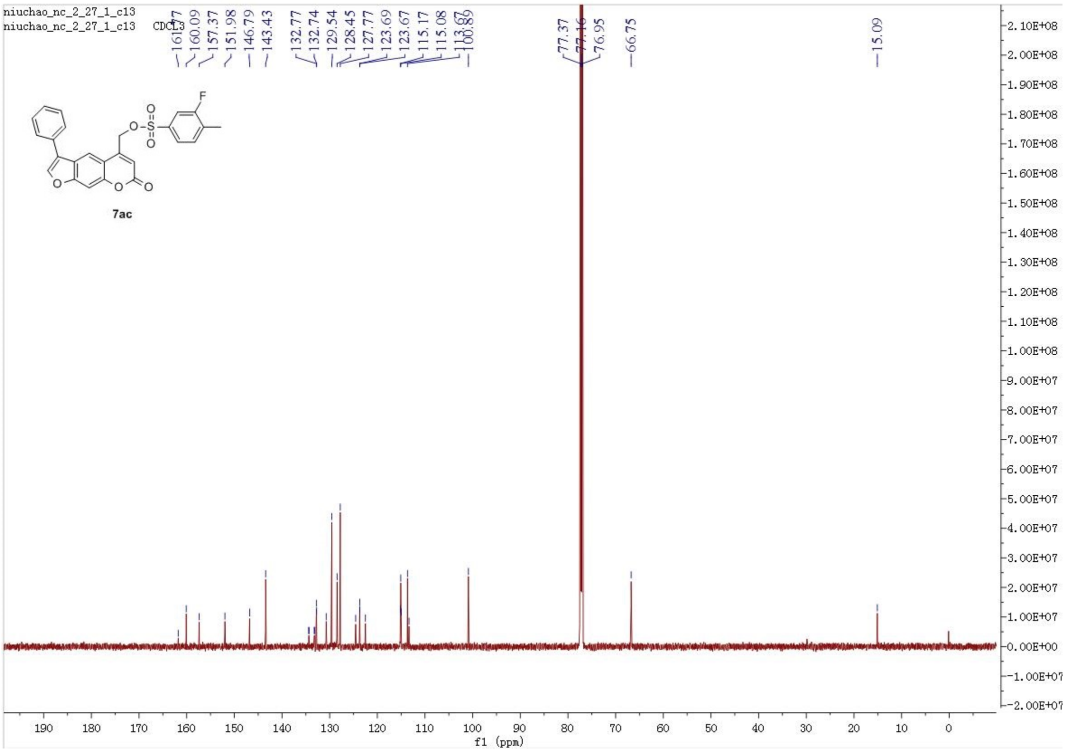

^{1}H NMR and ^{13}C NMR spectra of **7ad**

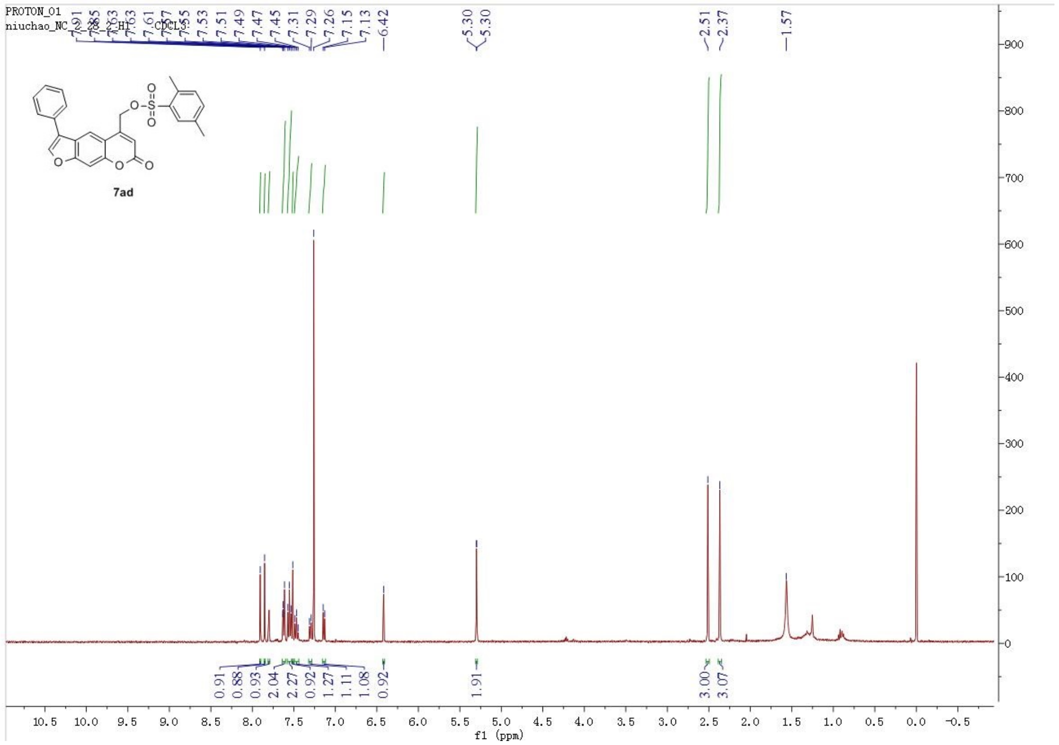

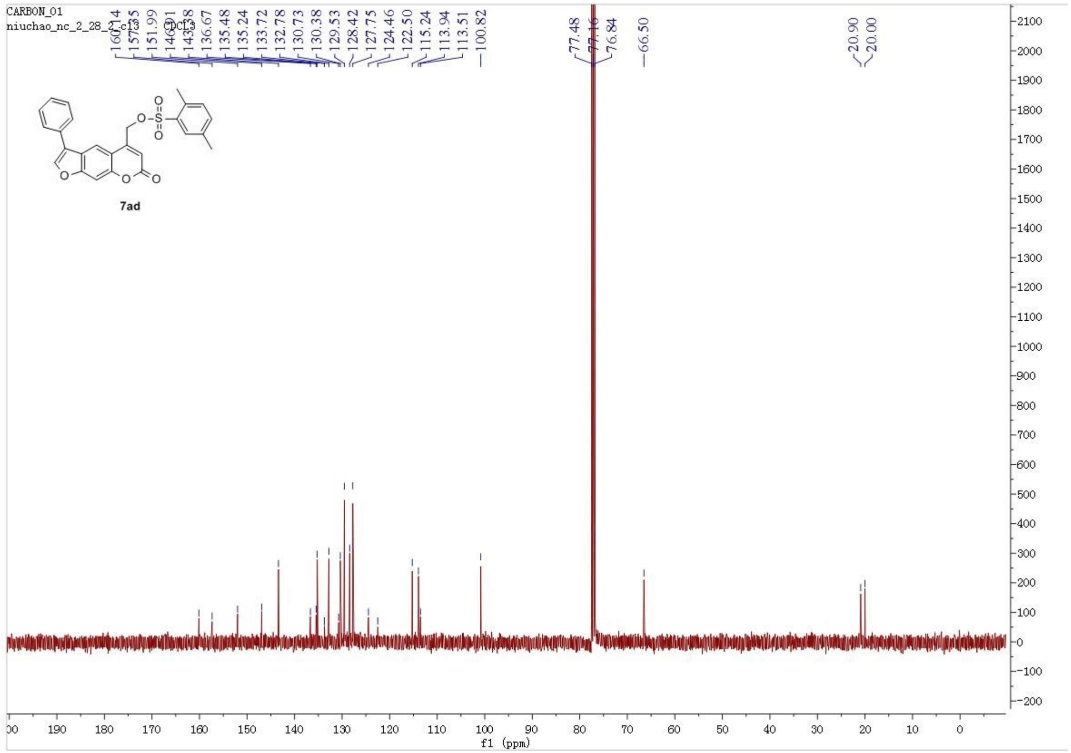

^{1}H NMR and ^{13}C NMR spectra of **8**

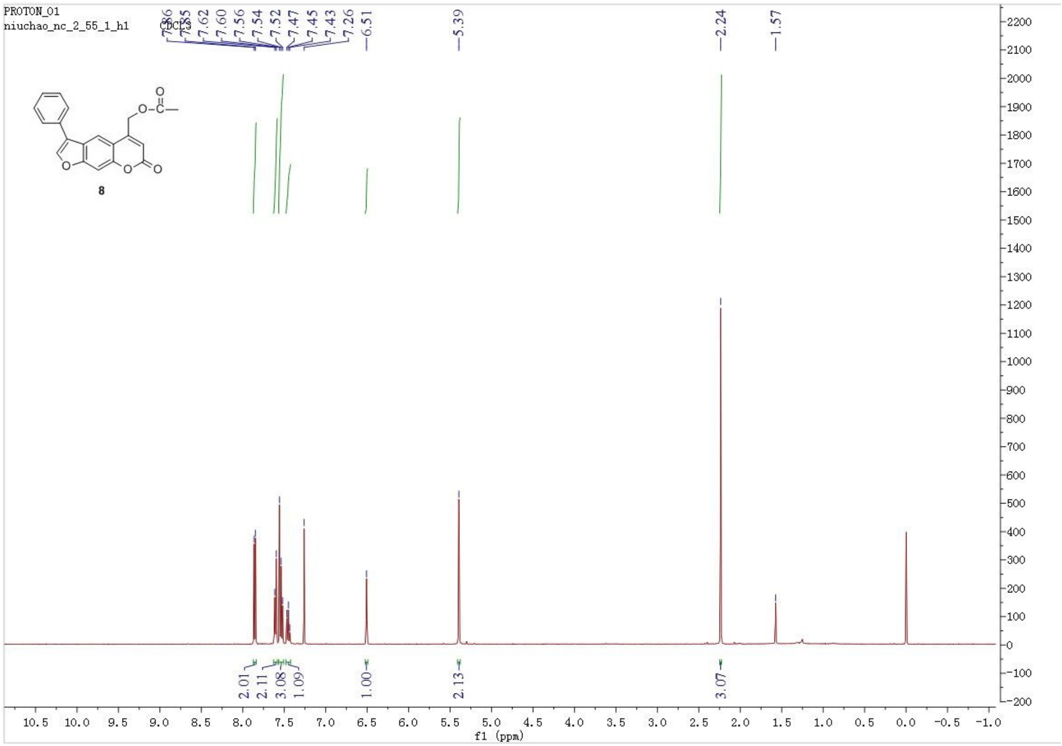

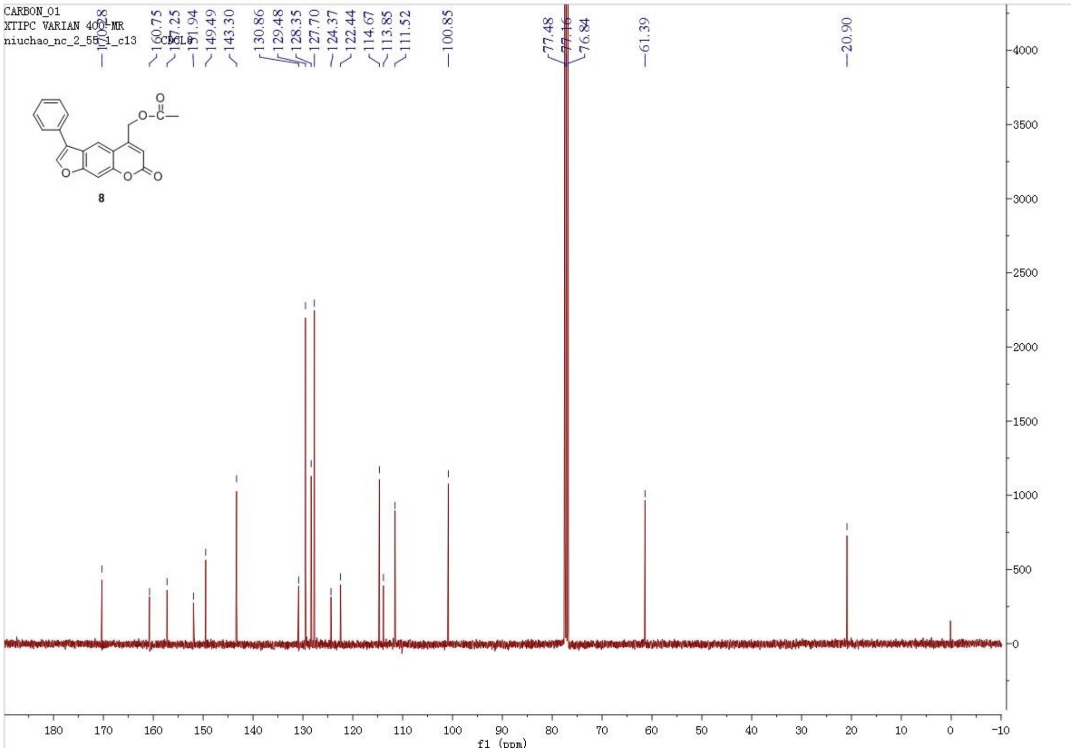

^{1}H NMR and ^{13}C NMR spectra of **8a**

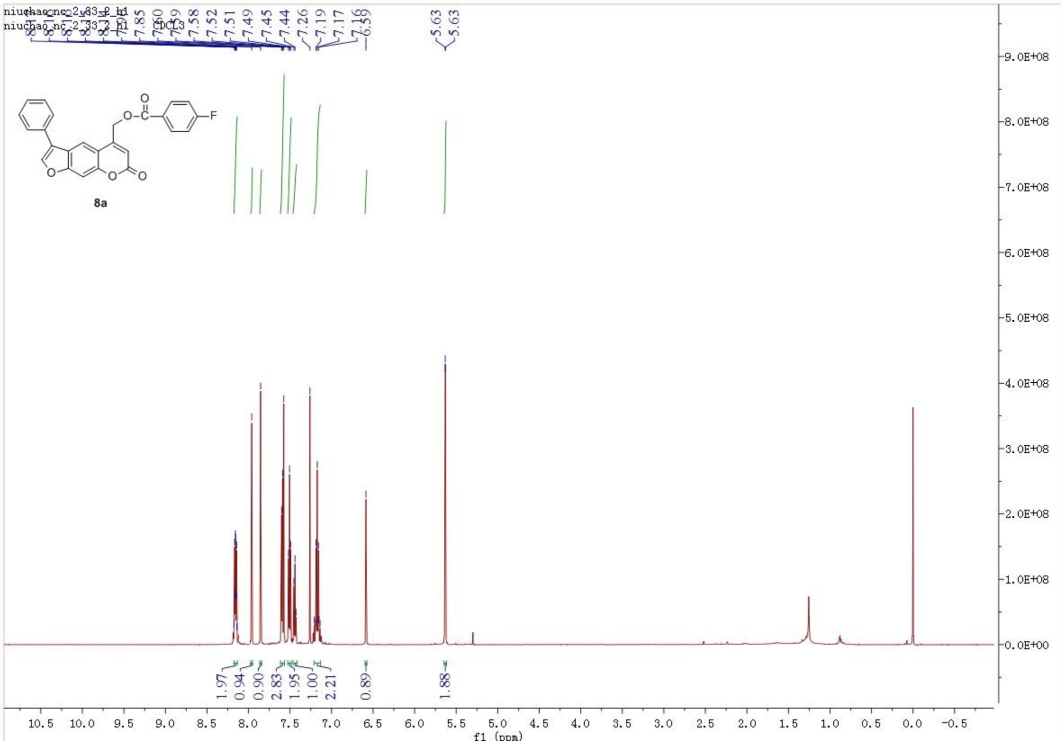

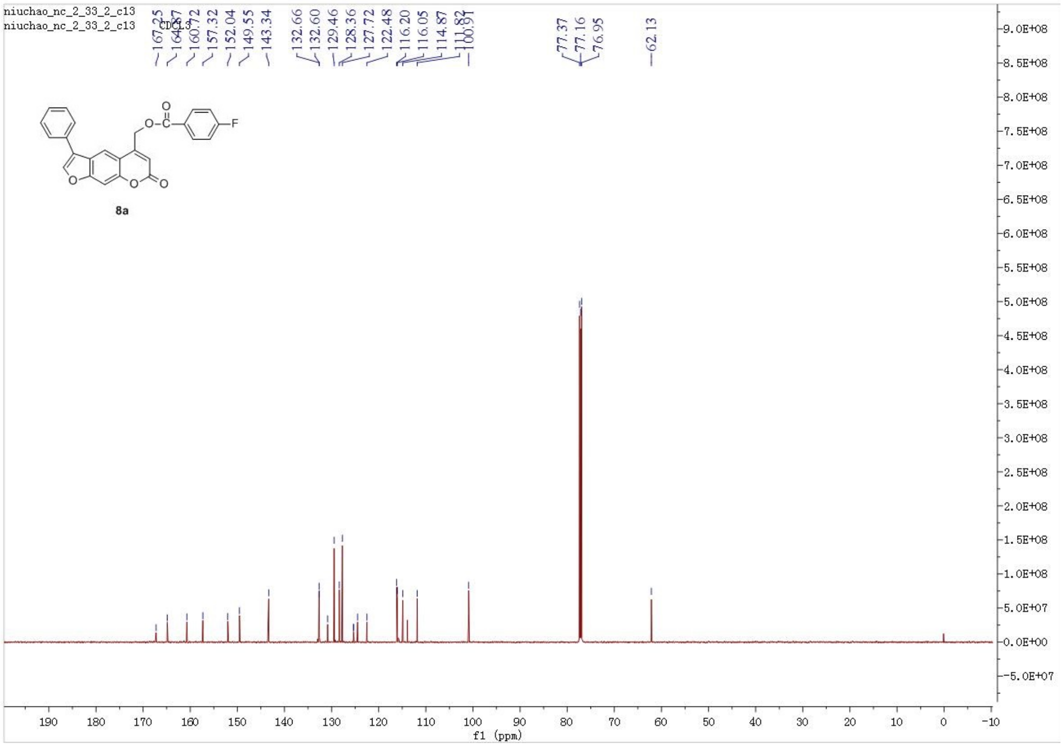

^{1}H NMR and ^{13}C NMR spectra of **8b**

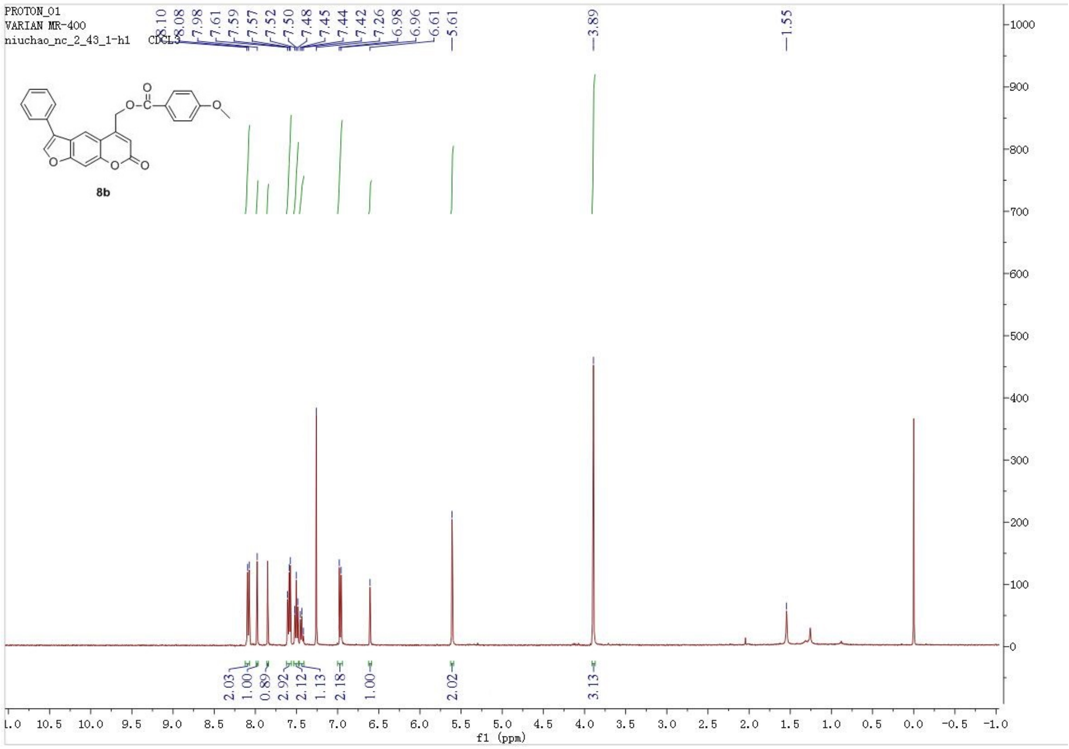

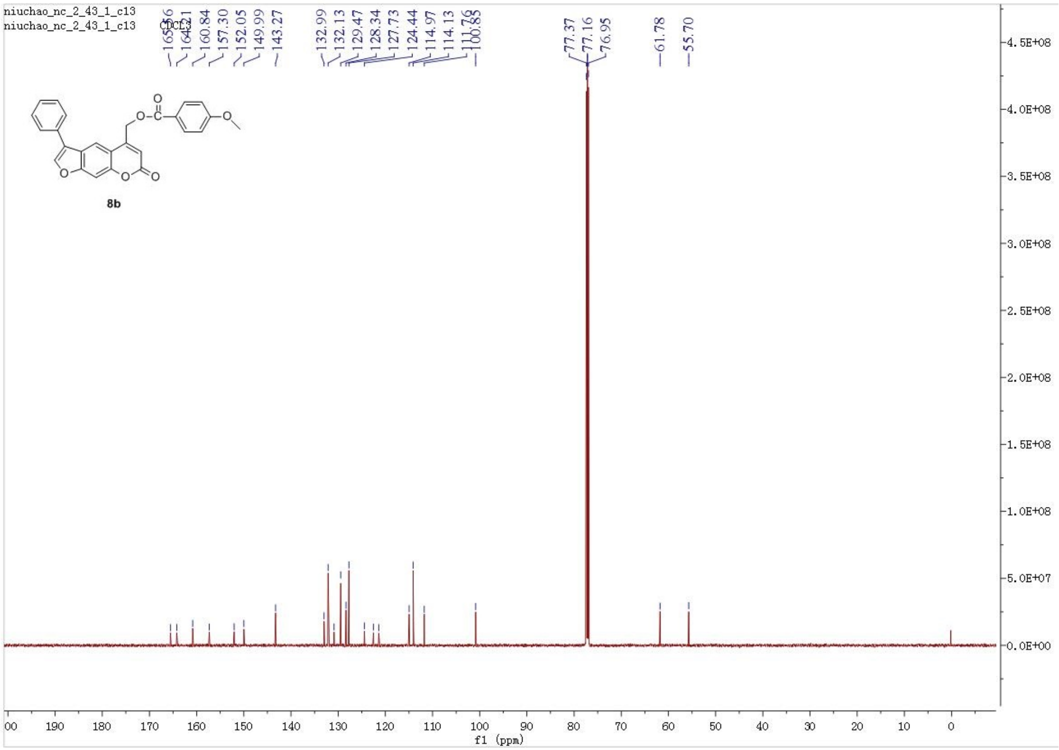

¹H NMR and ¹³C NMR spectra of **8c**

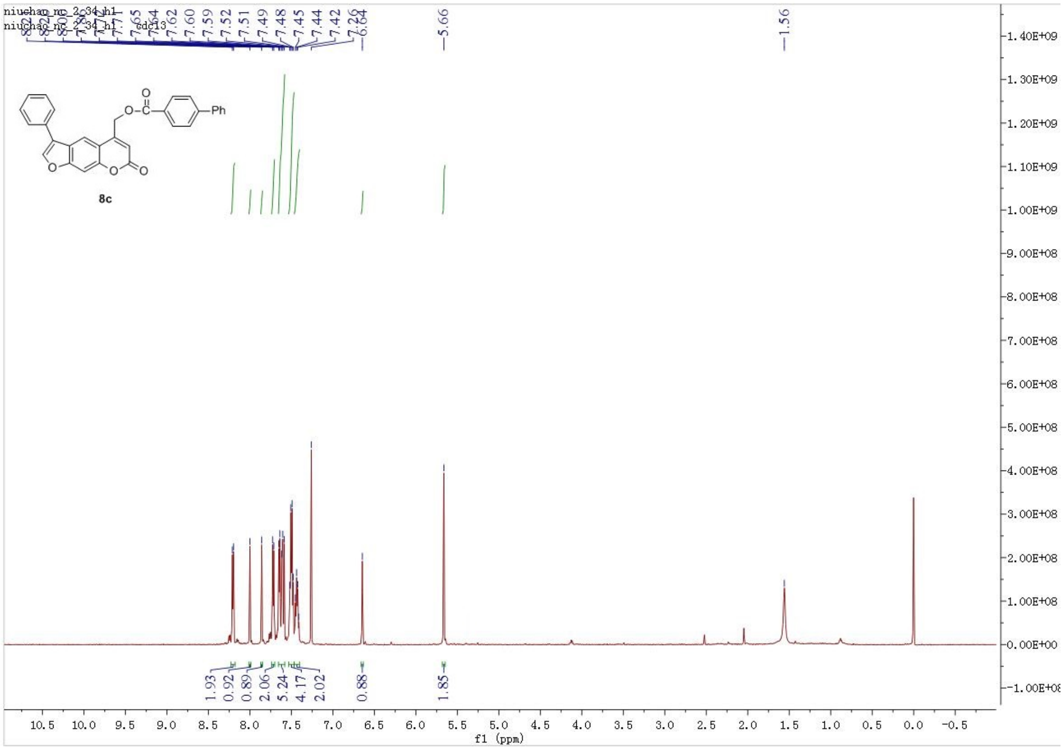

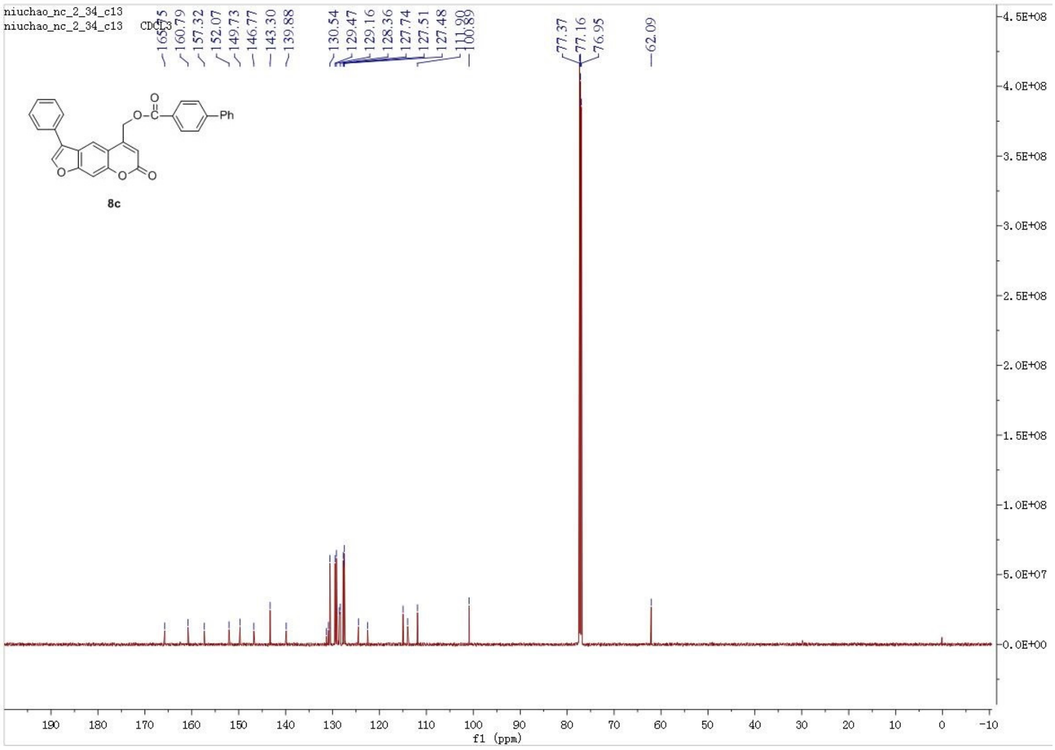

^{1}H NMR and ^{13}C NMR spectra of **8d**

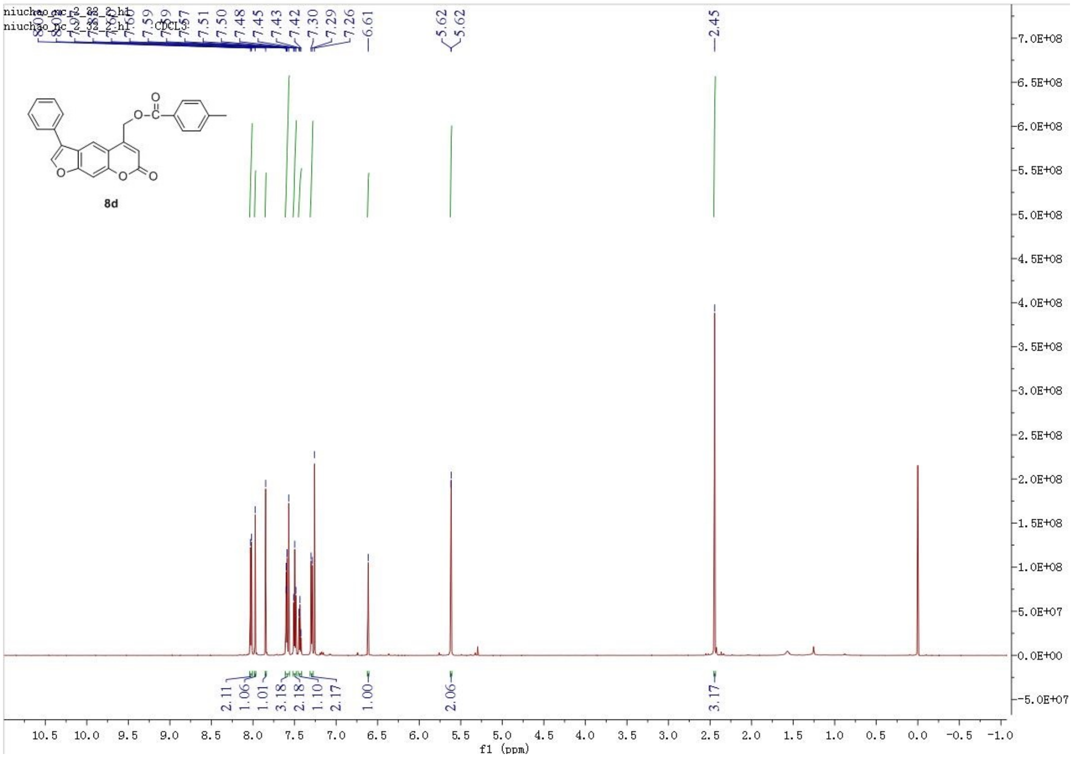

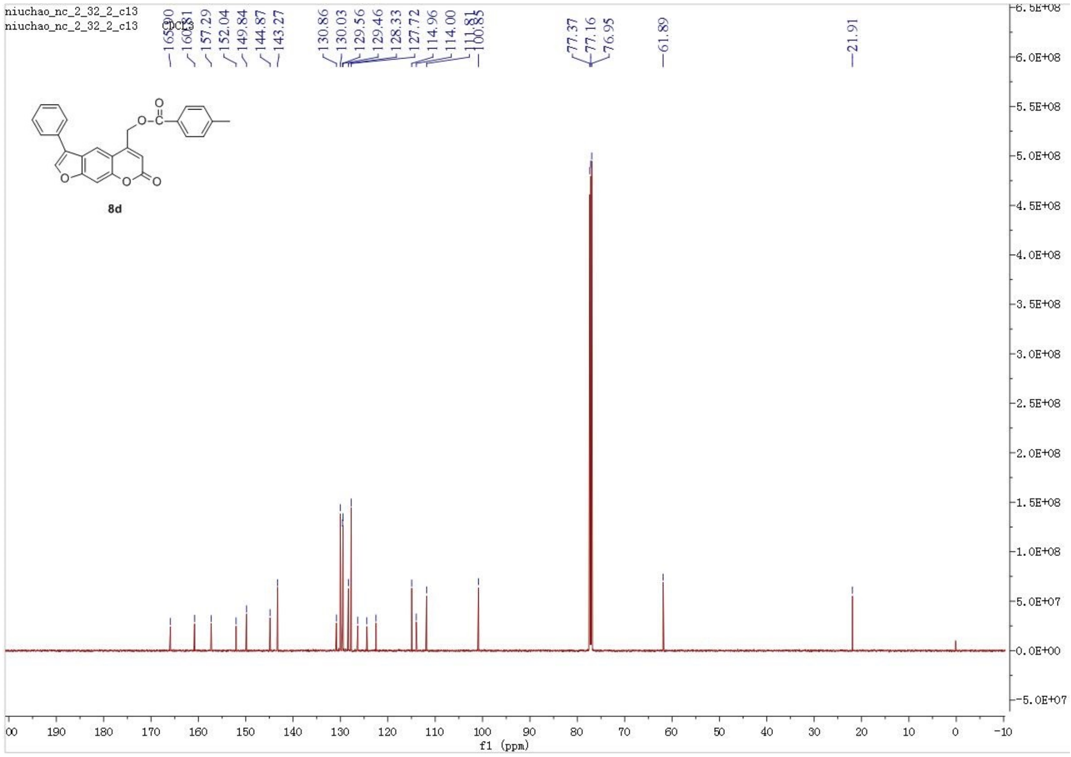

^{1}H NMR and ^{13}C NMR spectra of **8e**

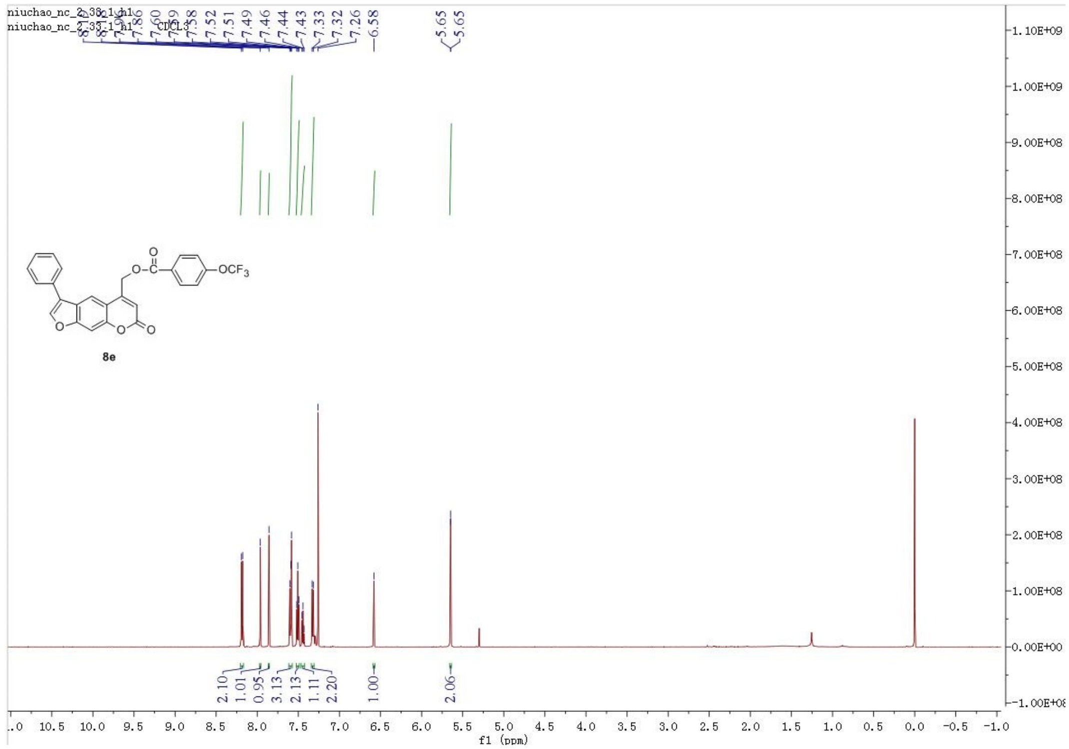

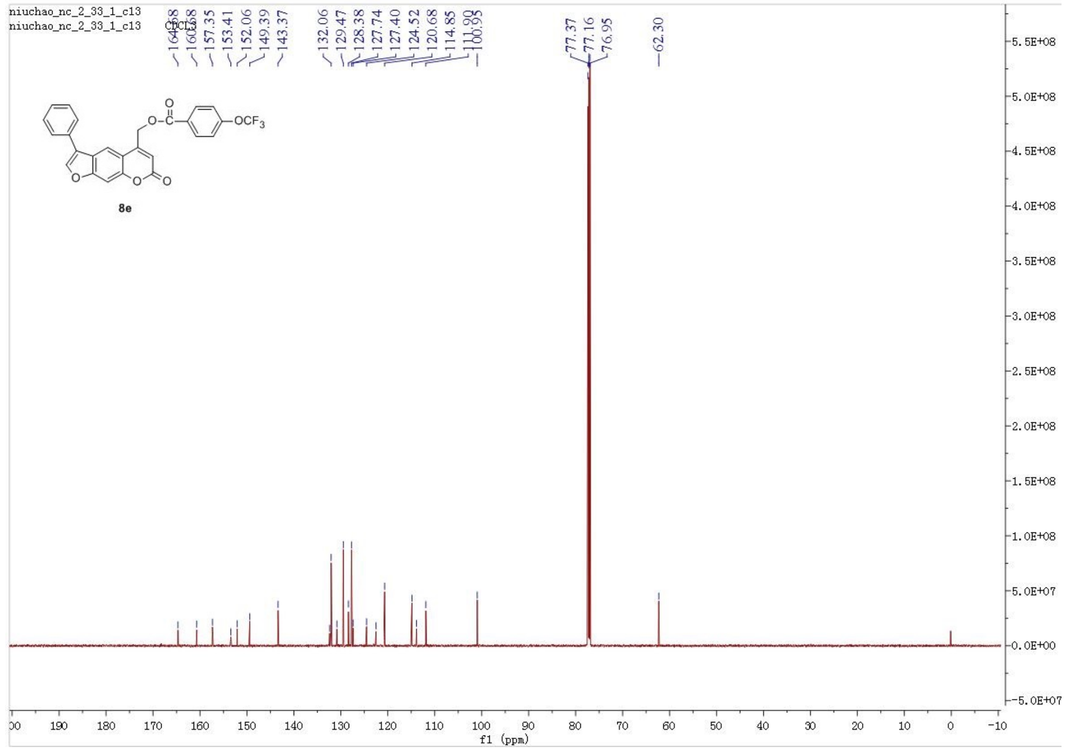

^{1}H NMR and ^{13}C NMR spectra of **8f**

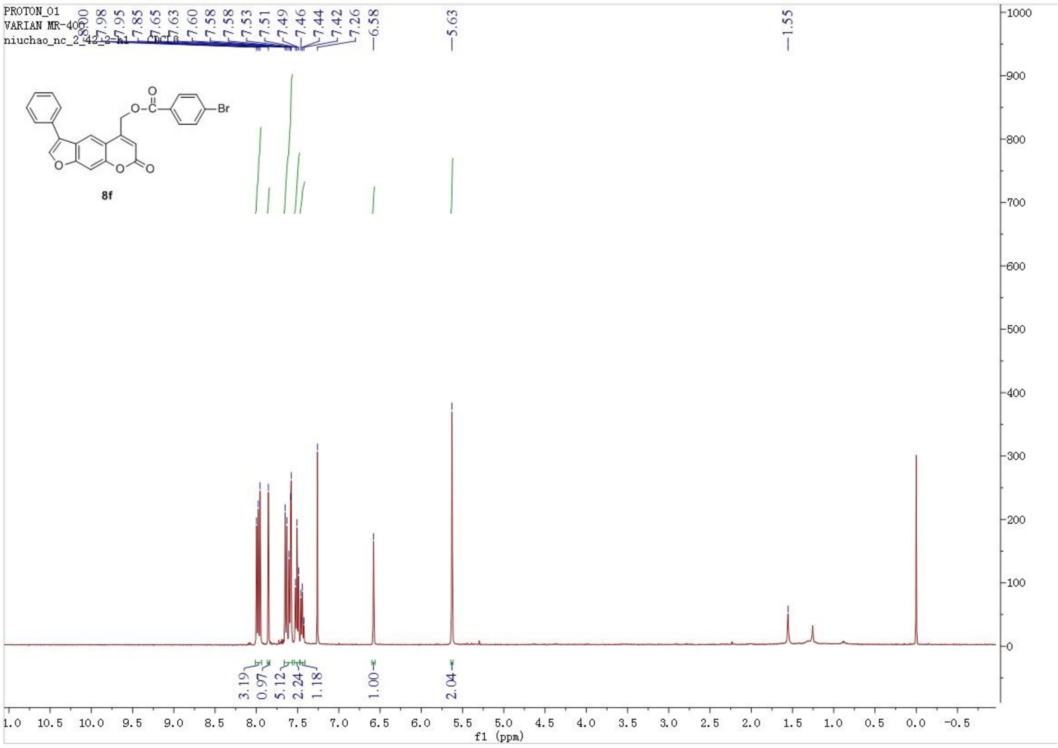

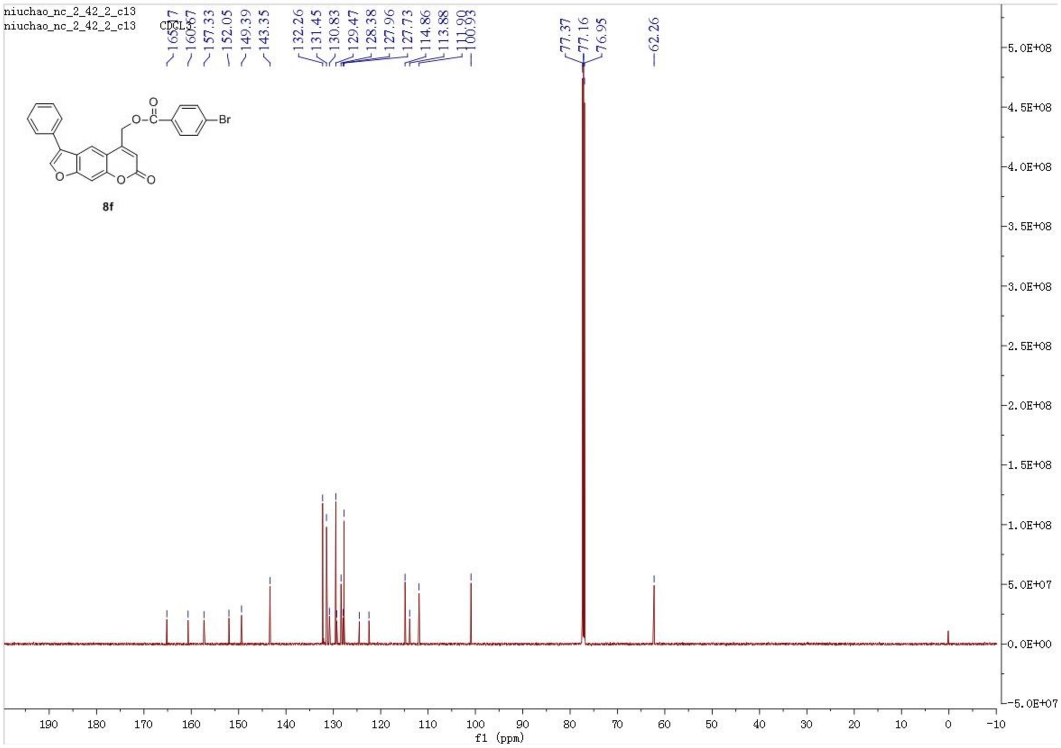

^{1}H NMR and ^{13}C NMR spectra of **8g**

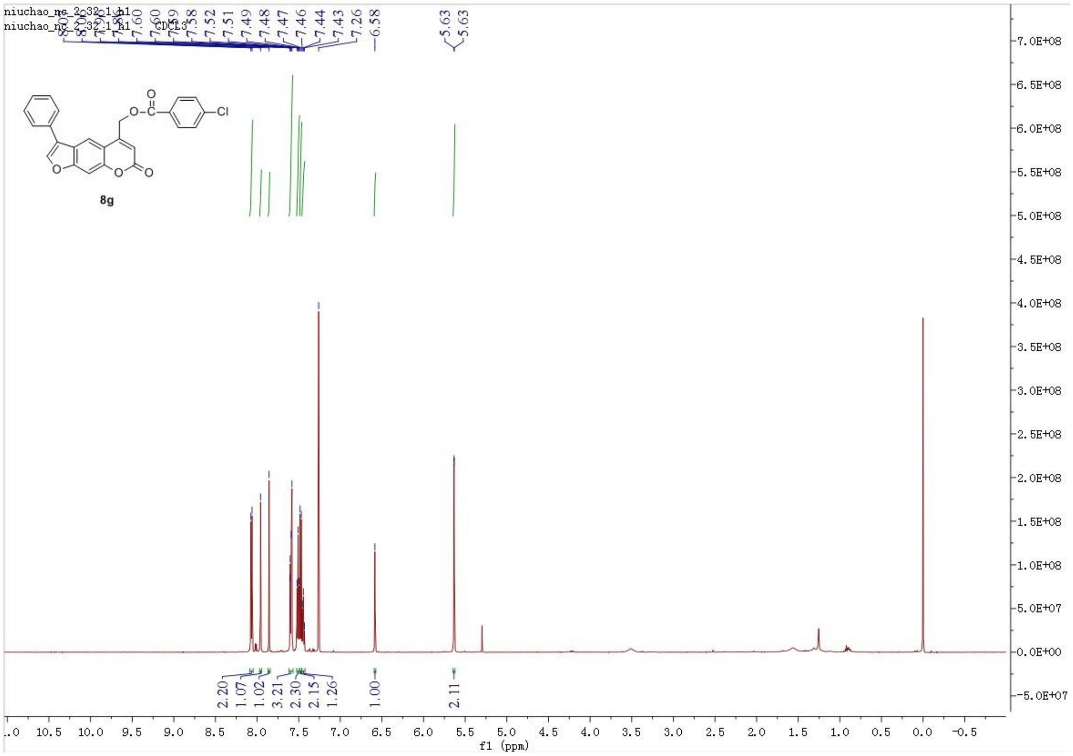

B2.16 衍生物 **8g** 的 ^{13}C NMR(100 MHz, $CDCl_3$)

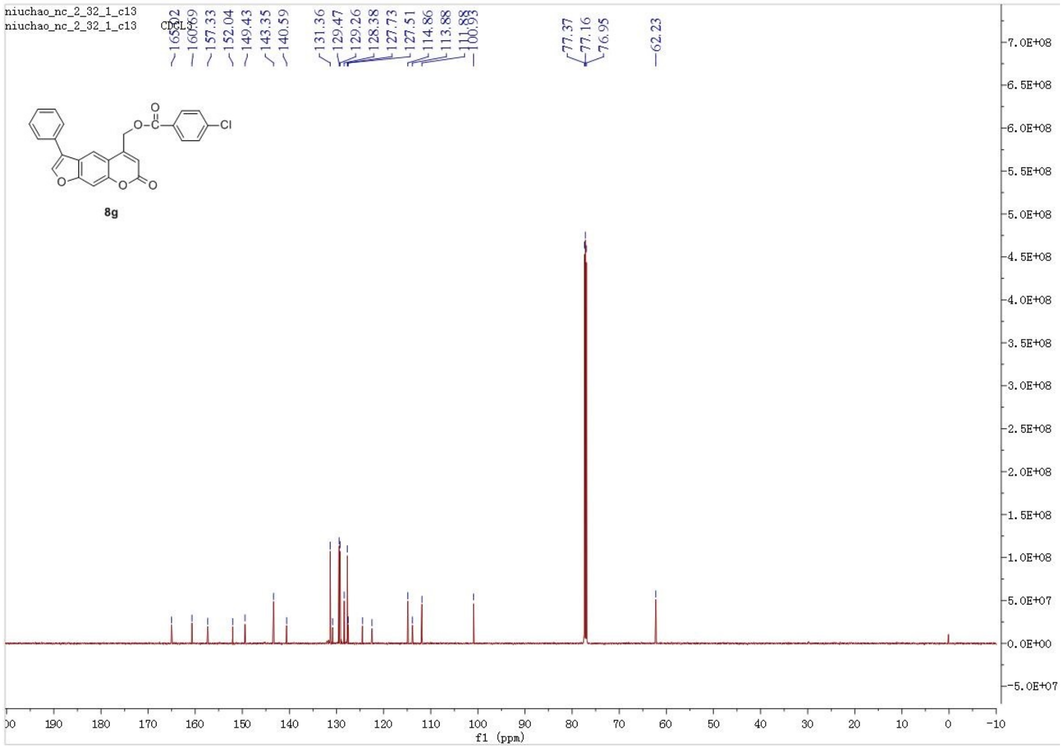

^{1}H NMR and ^{13}C NMR spectra of **8h**

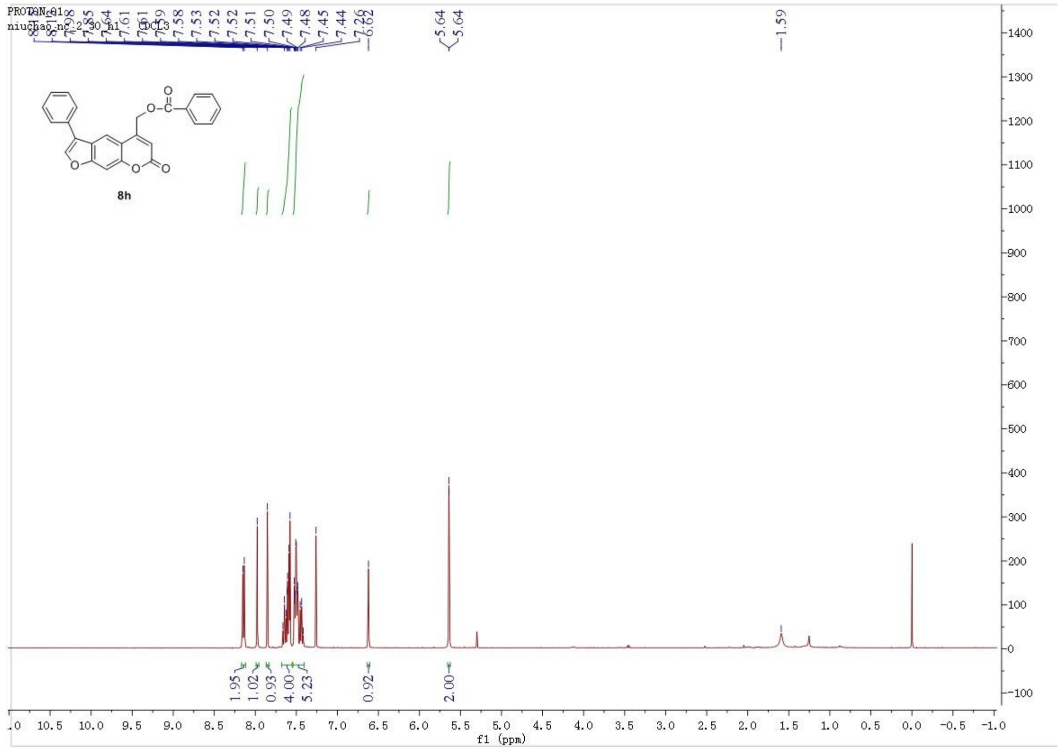

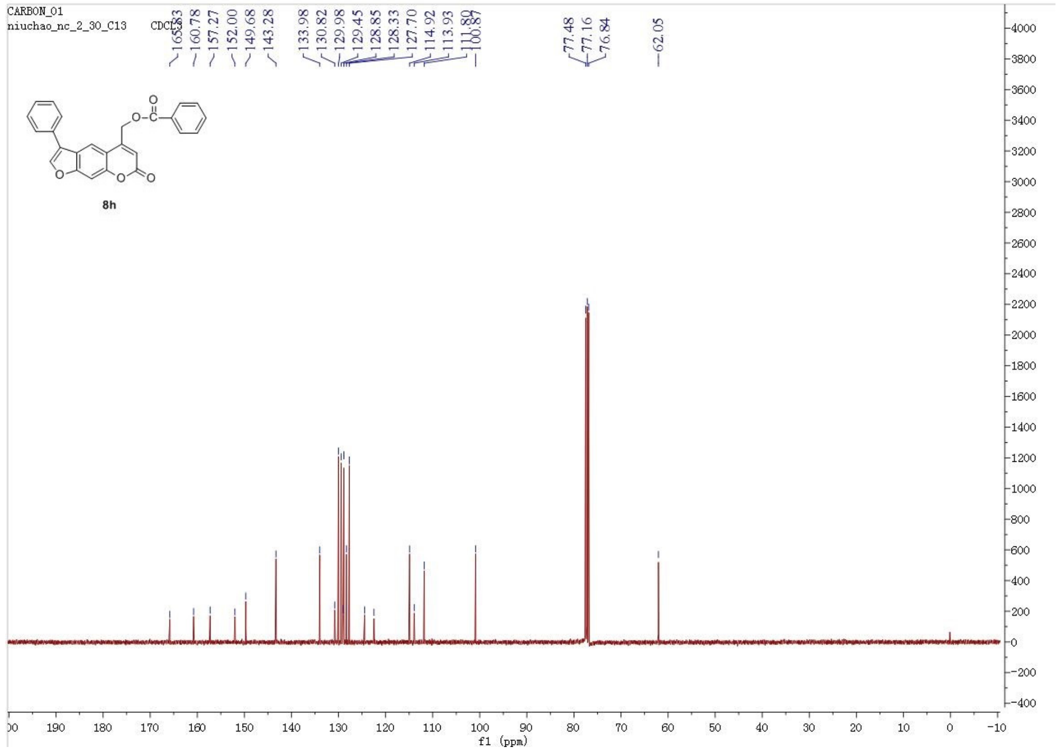

^{1}H NMR and ^{13}C NMR spectra of **8i**

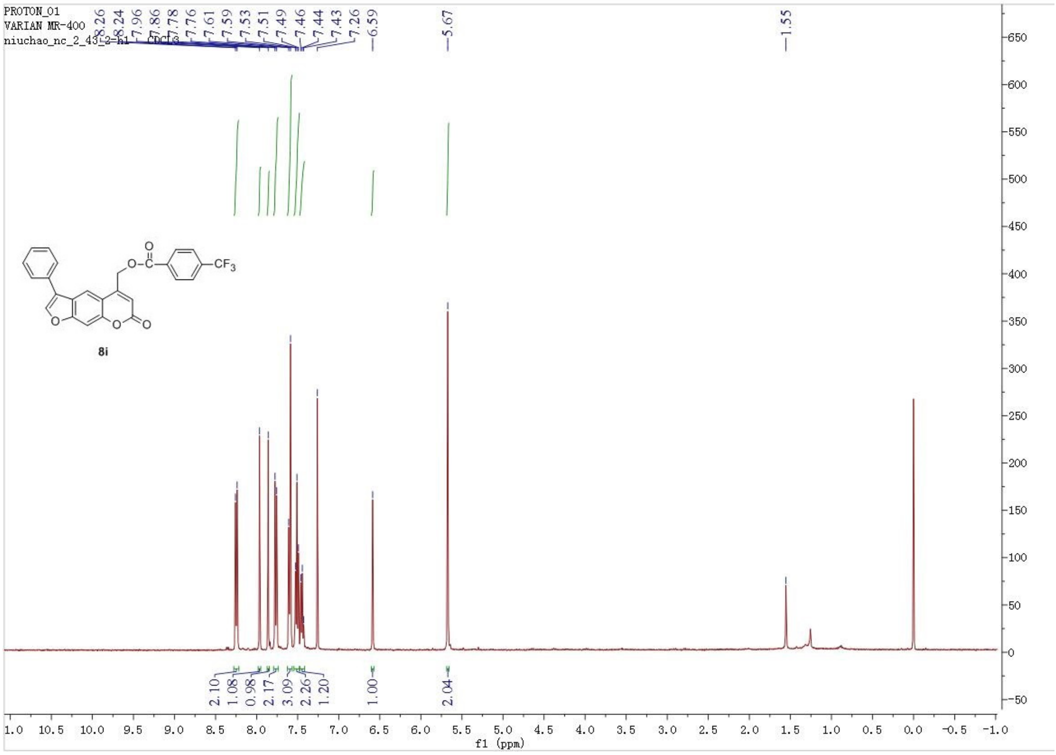

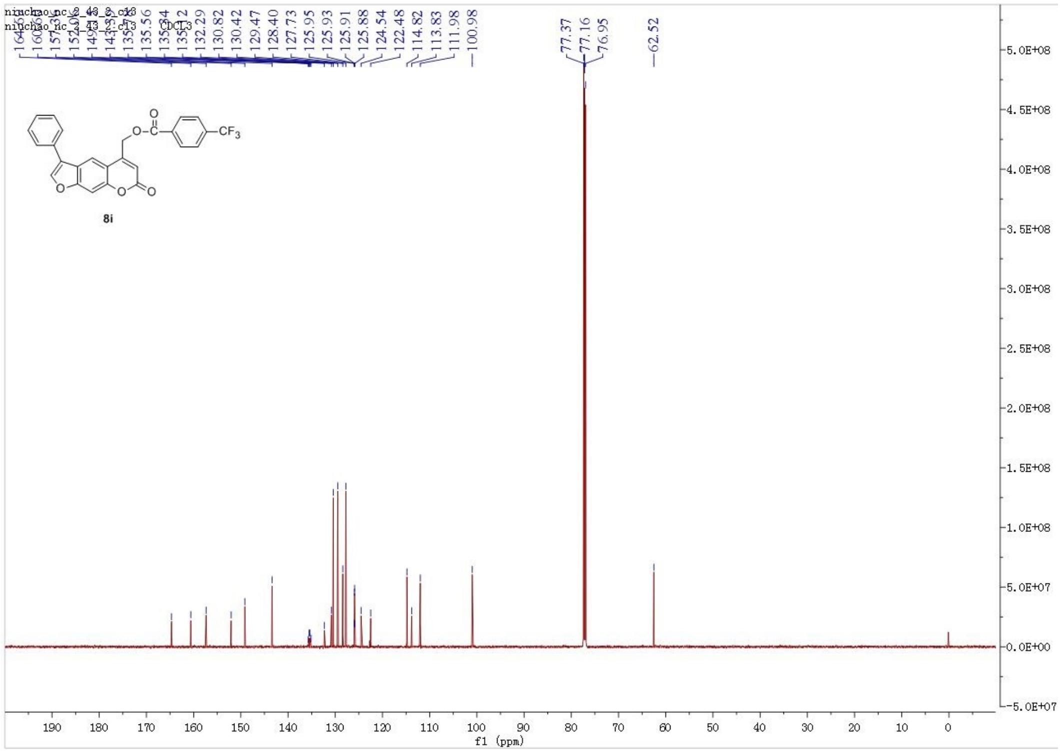

^{1}H NMR and ^{13}C NMR spectra of **8j**

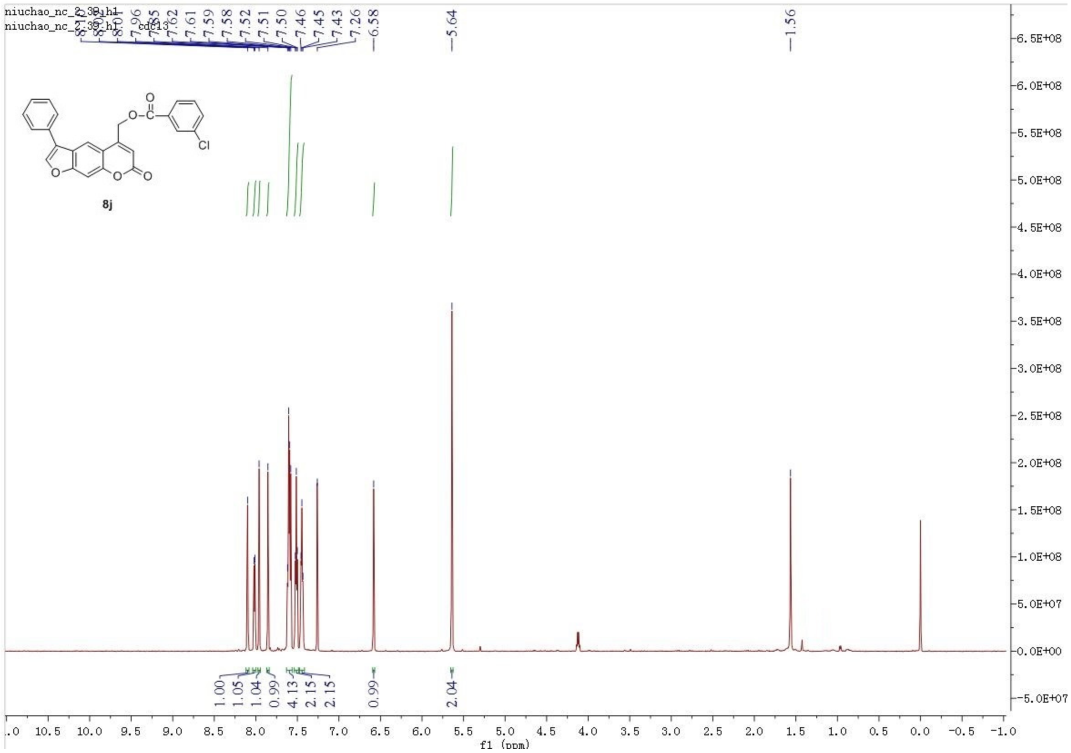

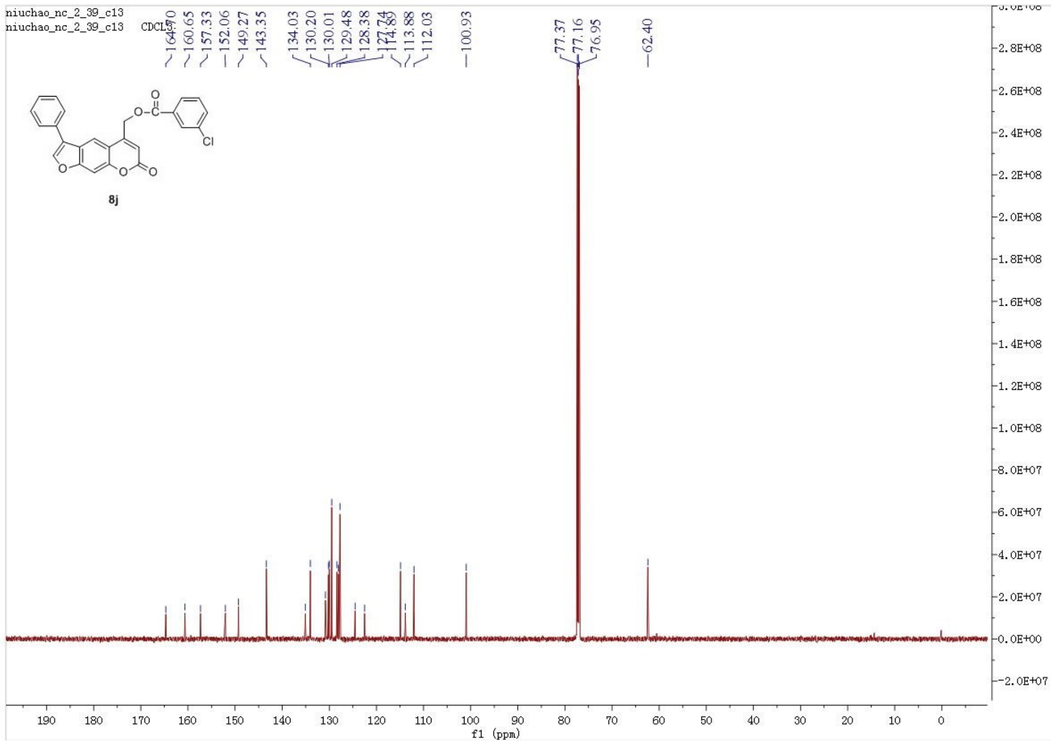

^{1}H NMR and ^{13}C NMR spectra of **8k**

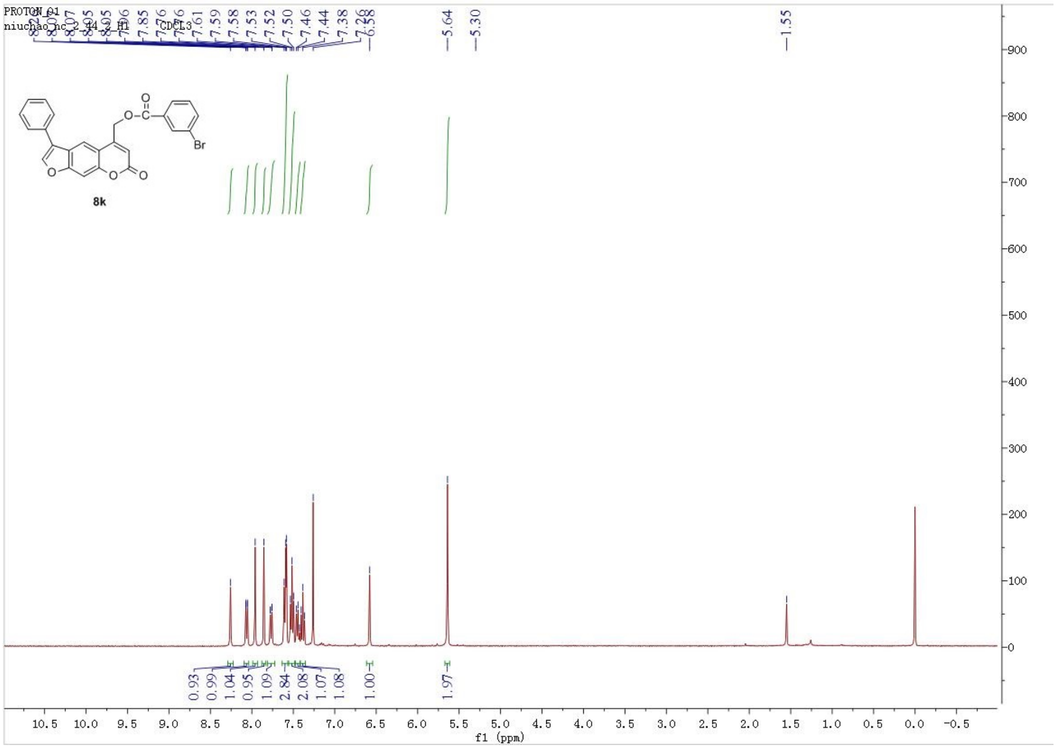

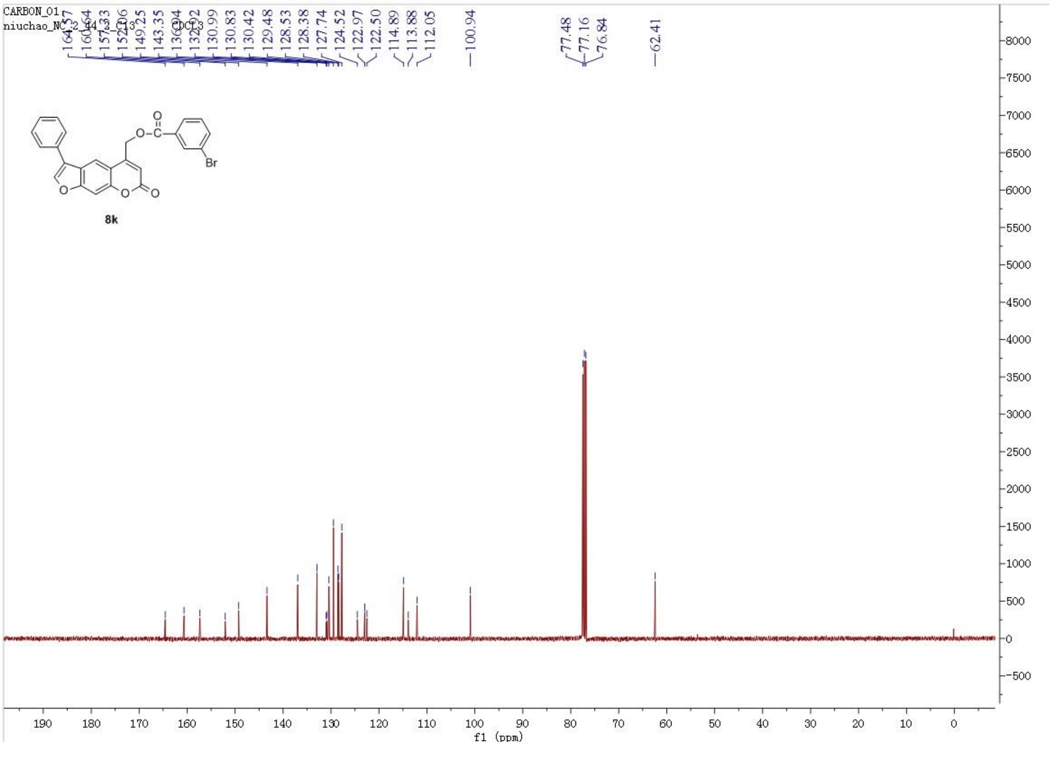

^{1}H NMR and ^{13}C NMR spectra of **8l**

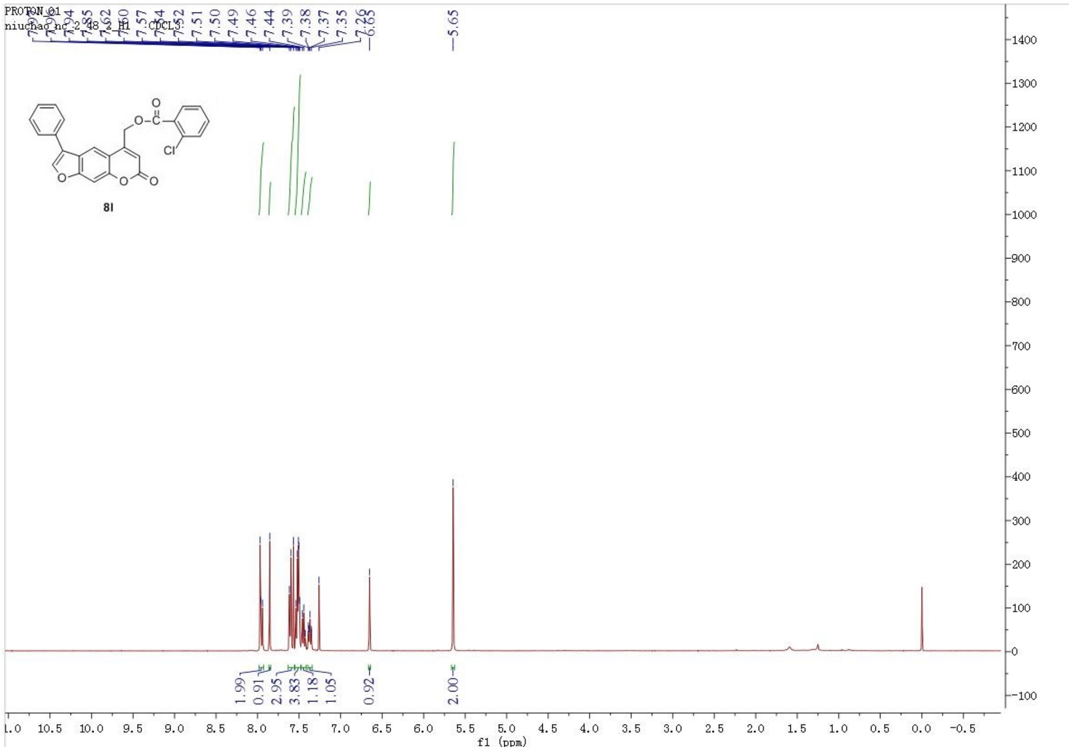

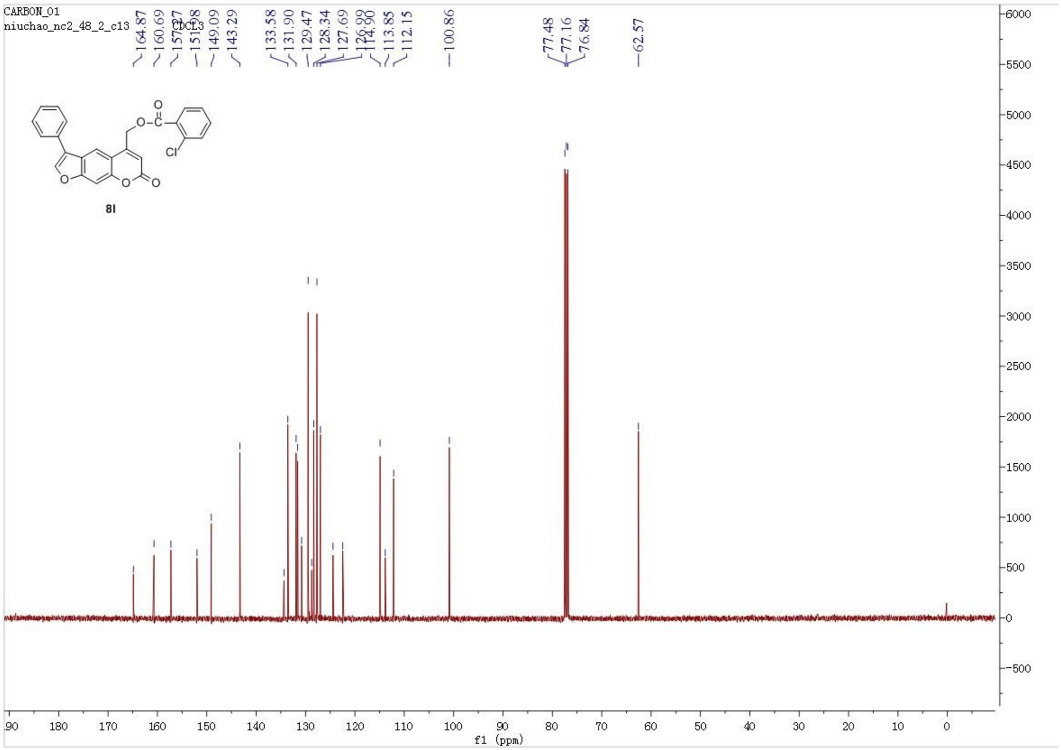

^{1}H NMR and ^{13}C NMR spectra of **8m**

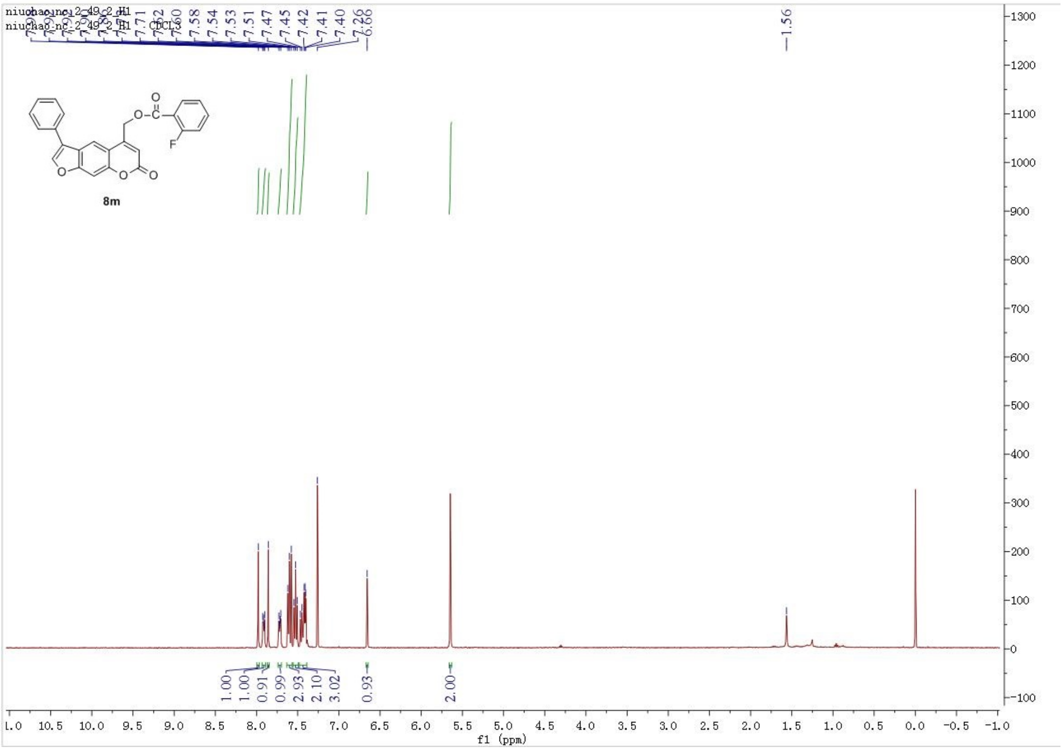

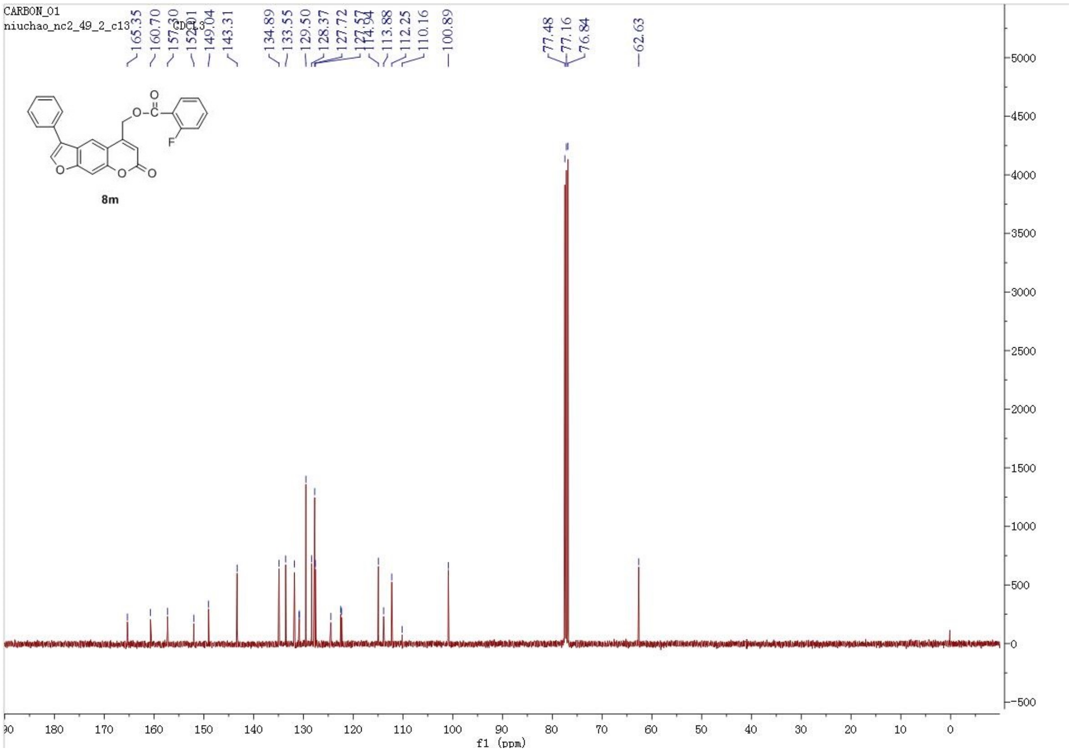

^{1}H NMR and ^{13}C NMR spectra of **8n**

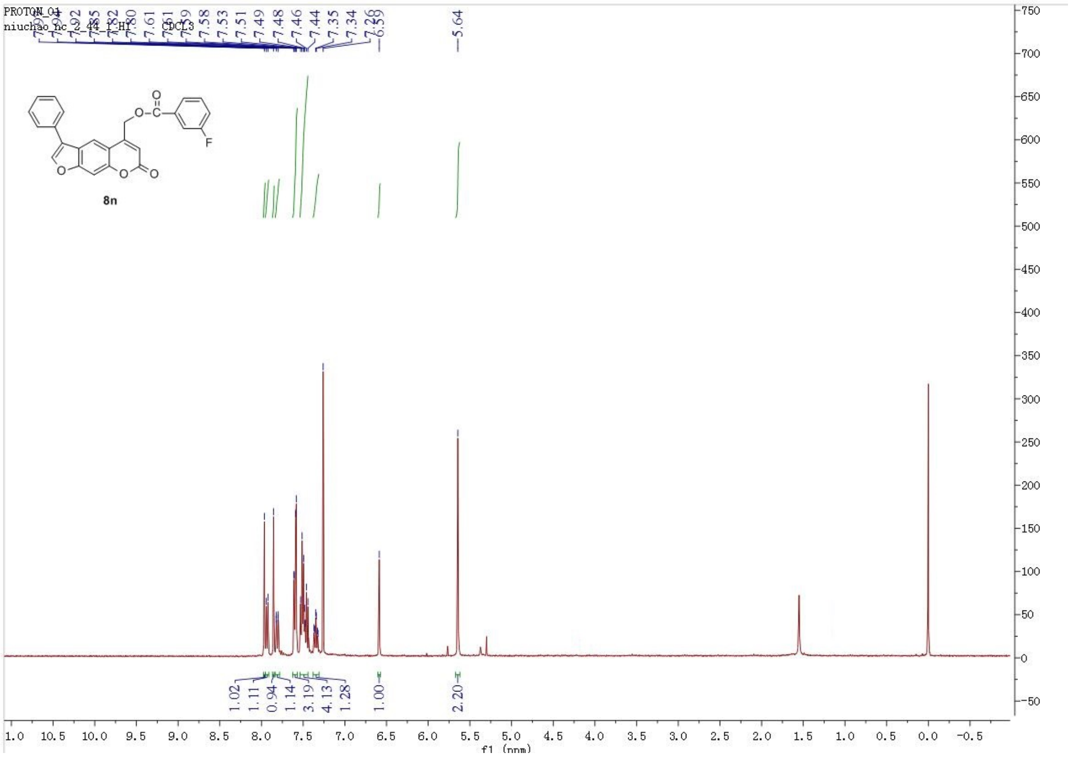

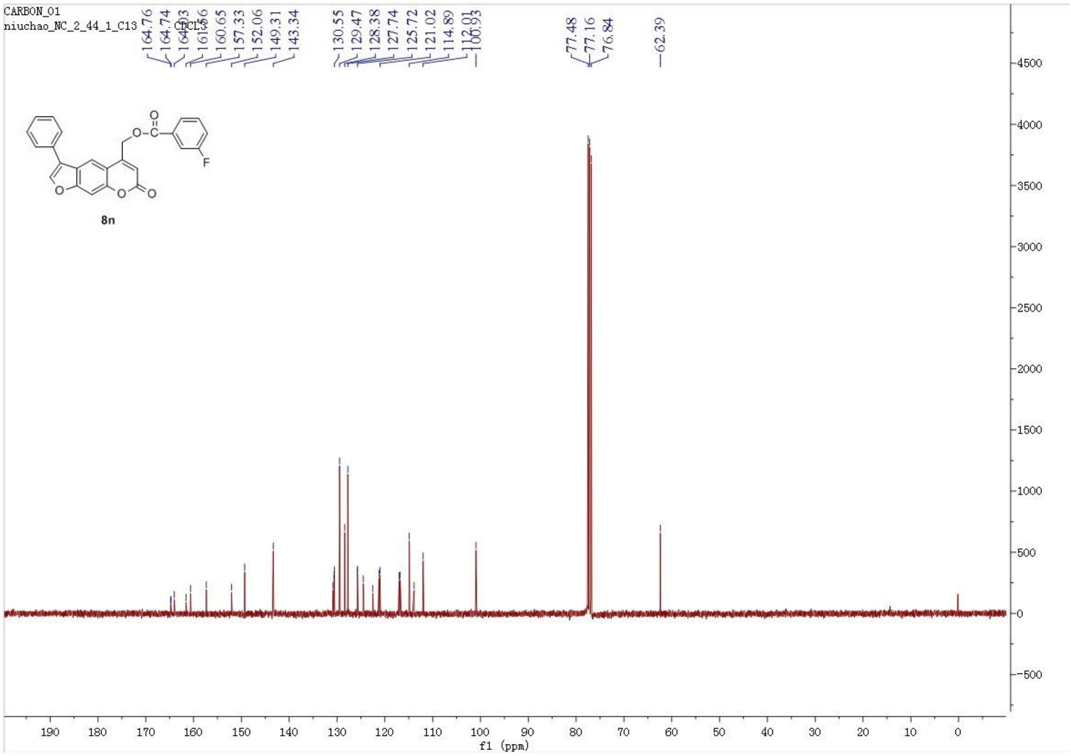

^{1}H NMR and ^{13}C NMR spectra of **8o**

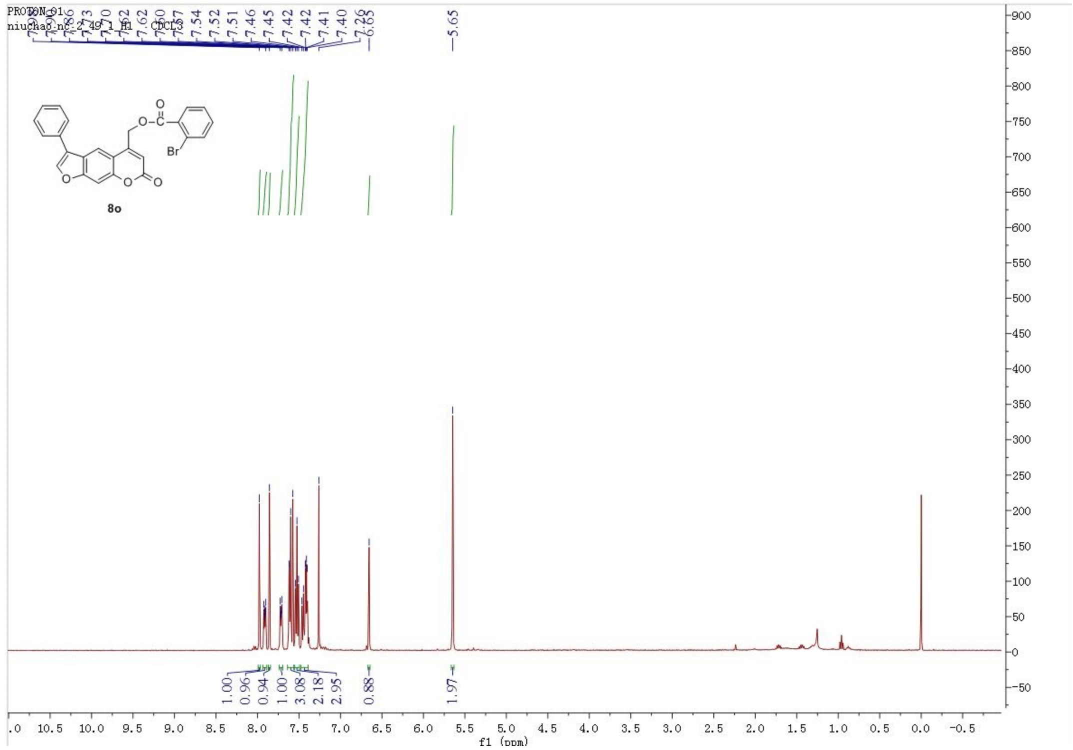

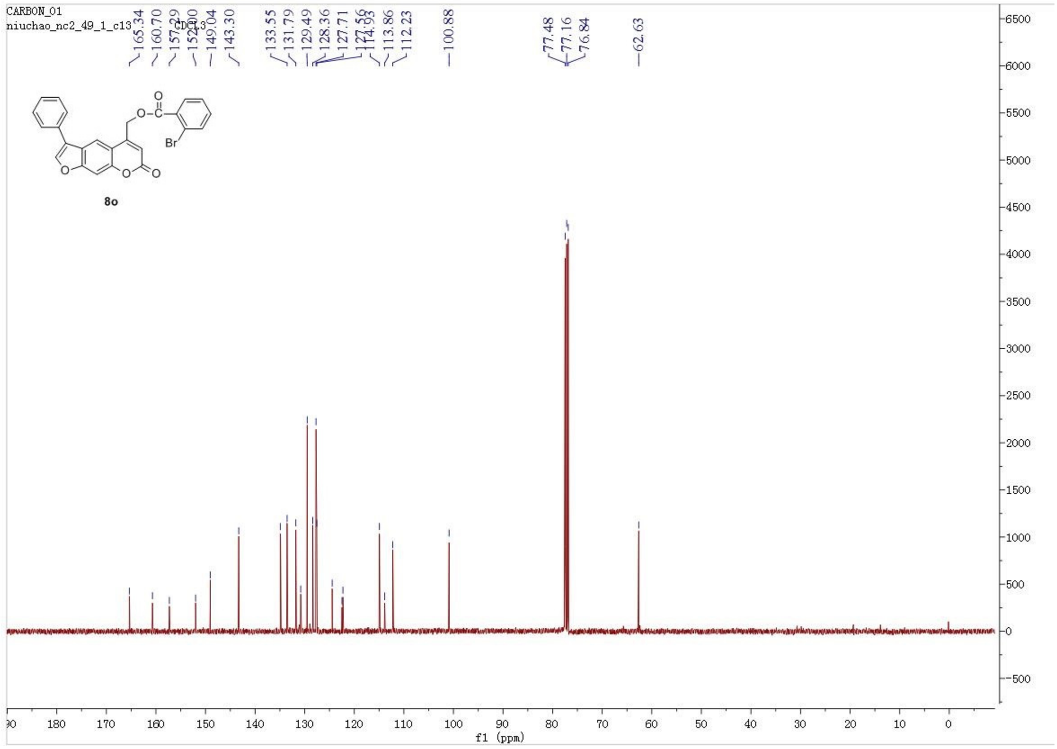

^{1}H NMR and ^{13}C NMR spectra of **8p**

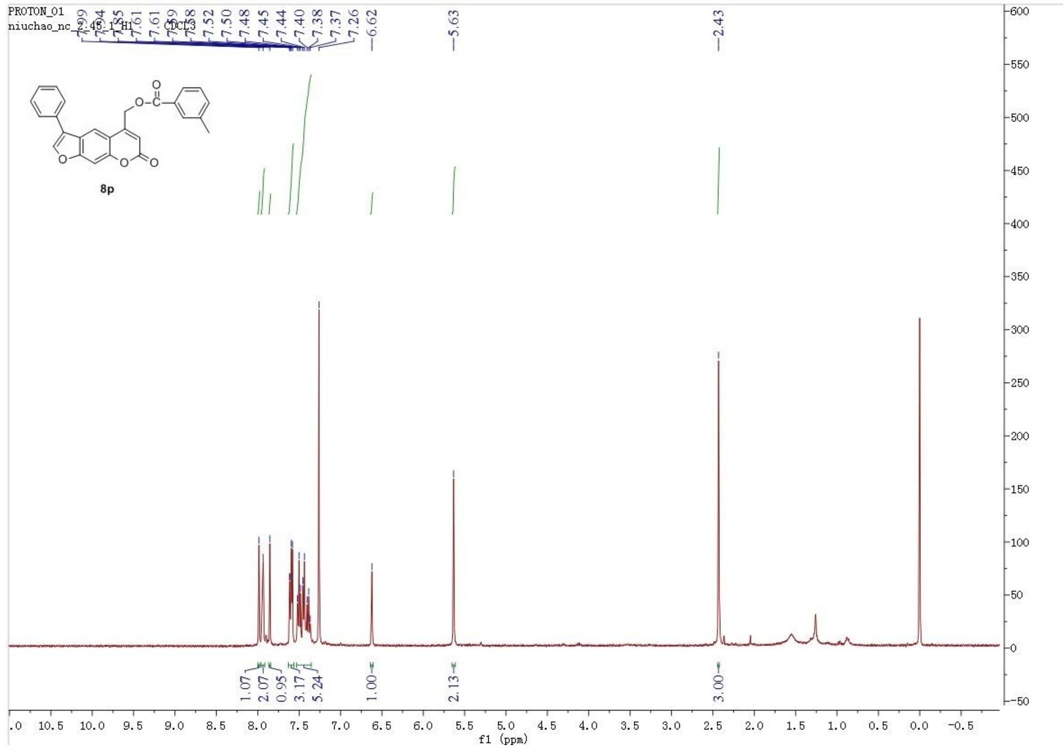

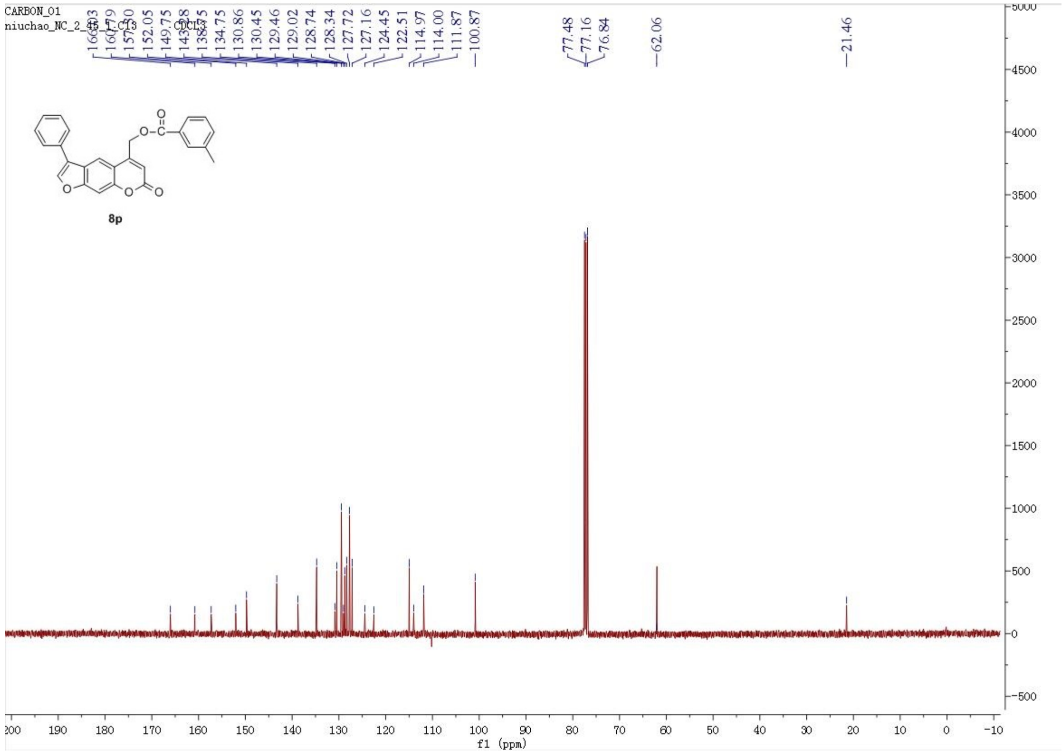

^{1}H NMR and ^{13}C NMR spectra of **8q**

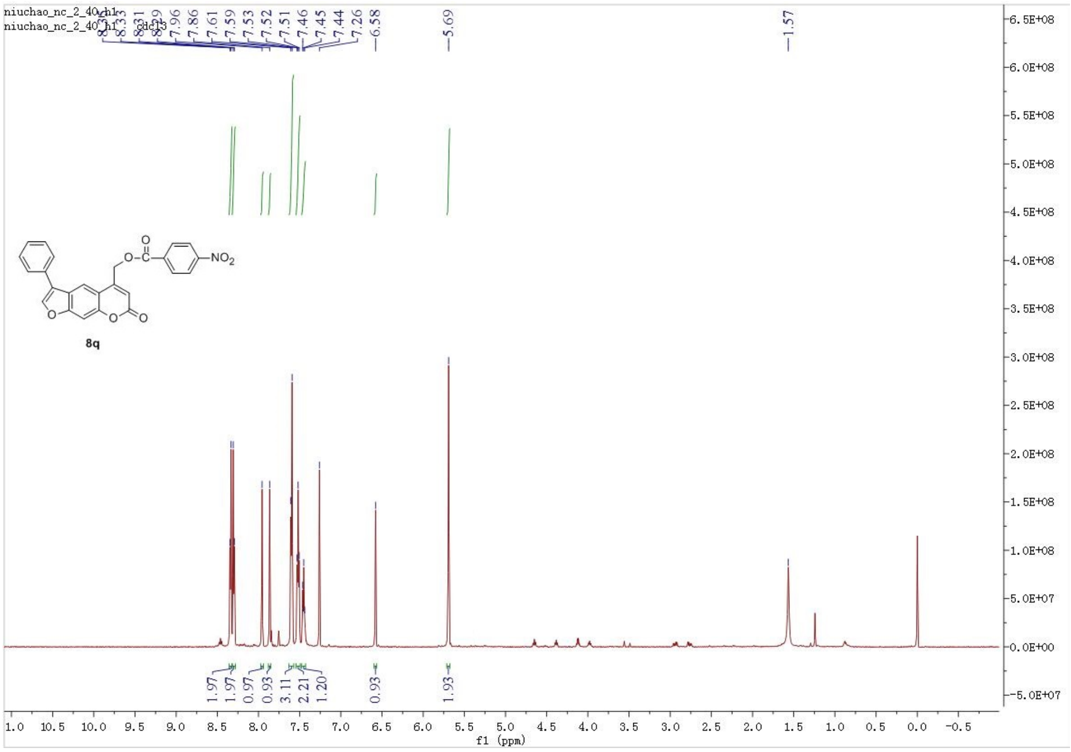

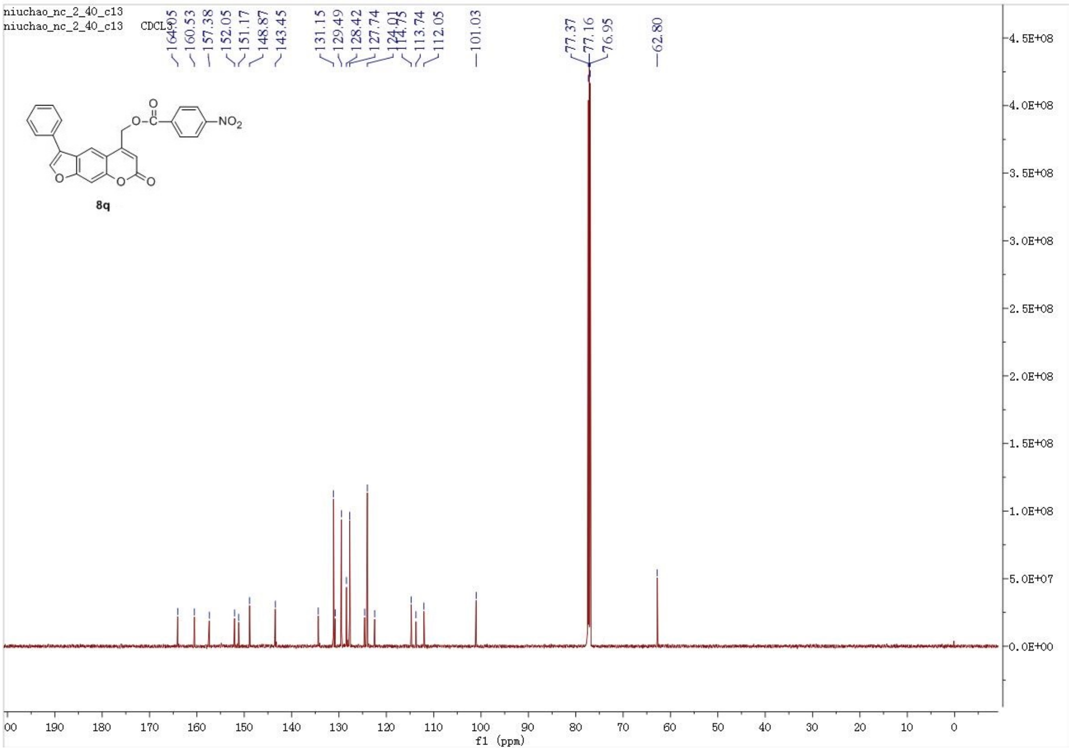

^{1}H NMR and ^{13}C NMR spectra of **8r**

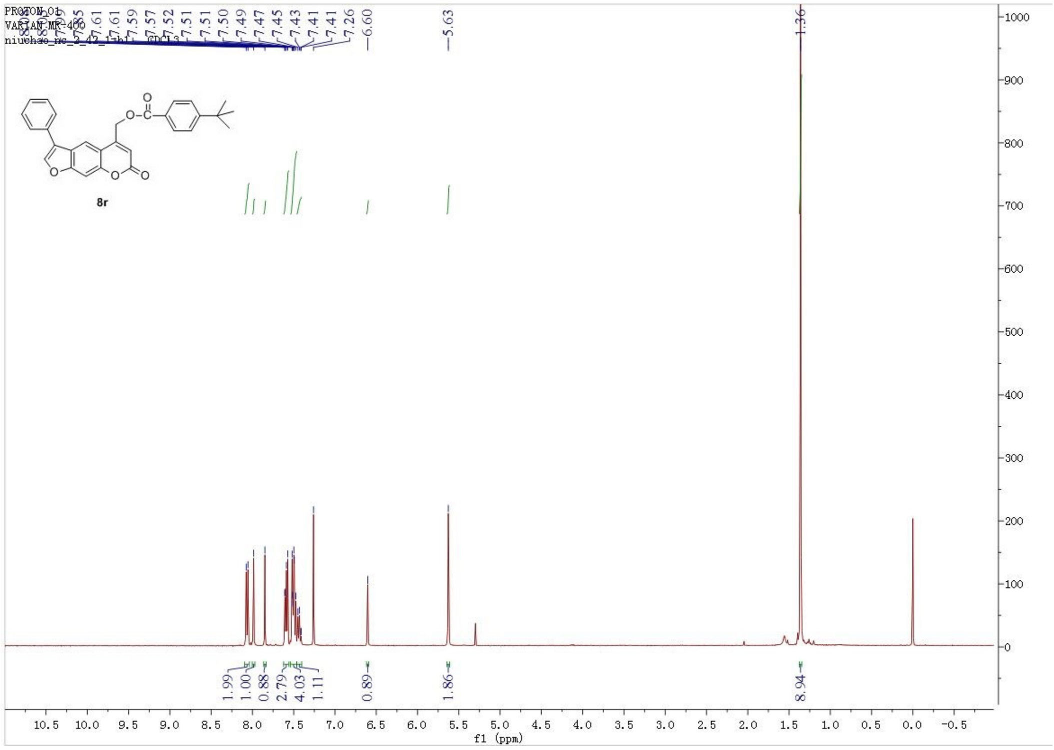

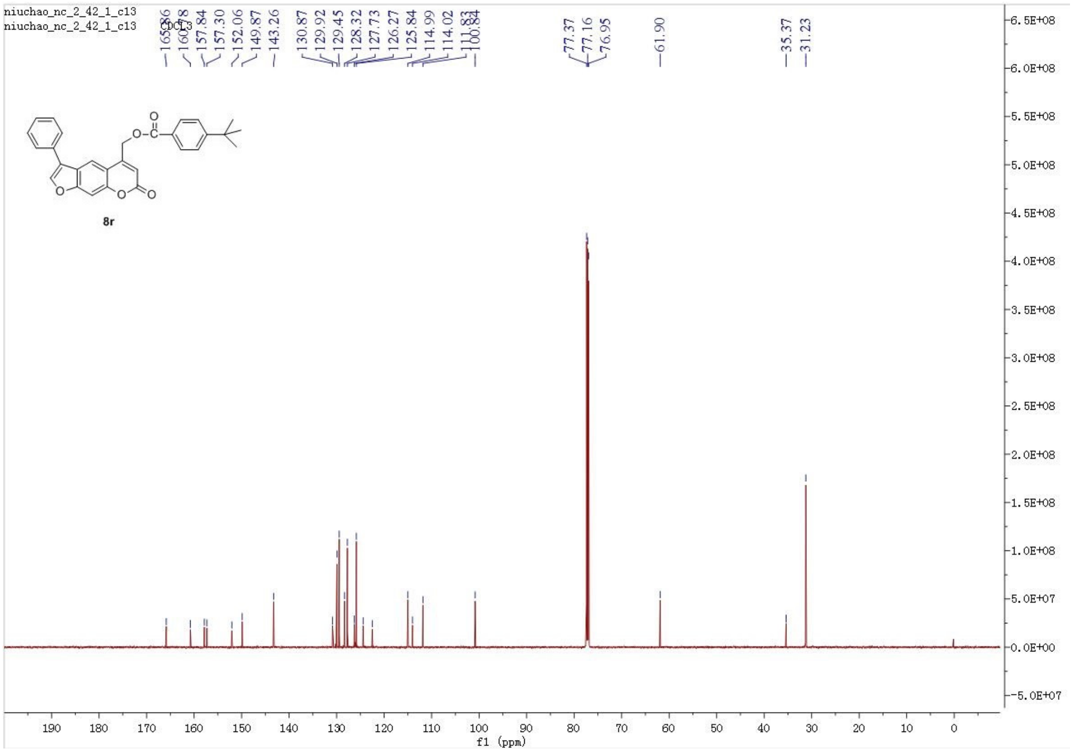

^{1}H NMR and ^{13}C NMR spectra of **8s**

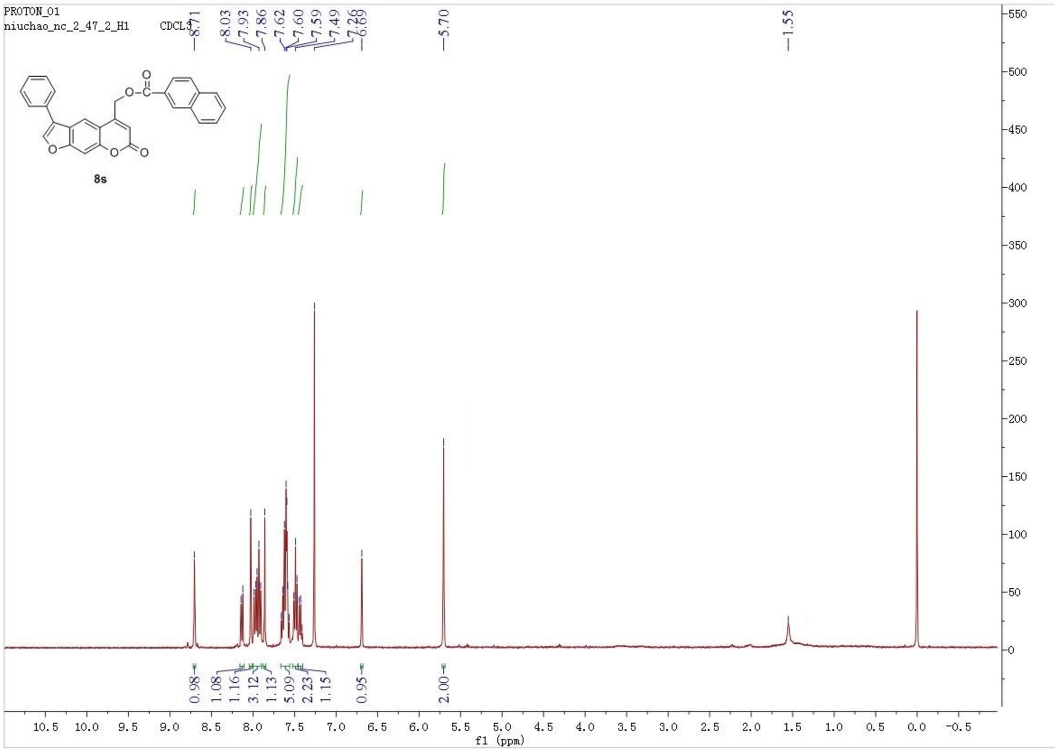

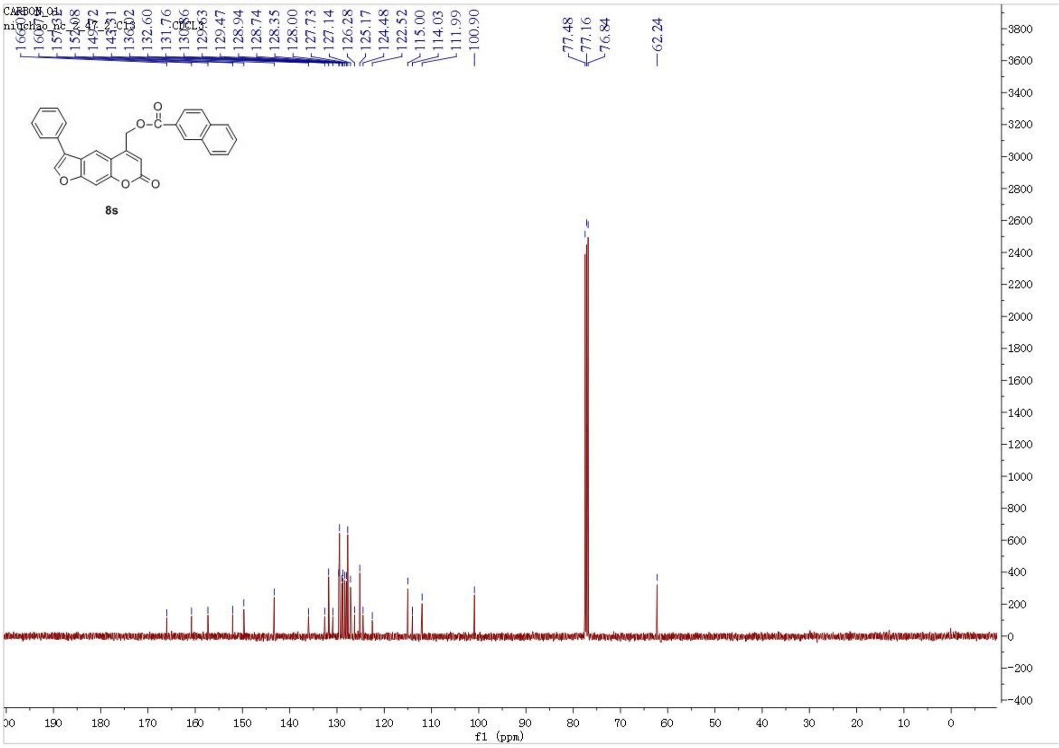

^{1}H NMR and ^{13}C NMR spectra of **8t**

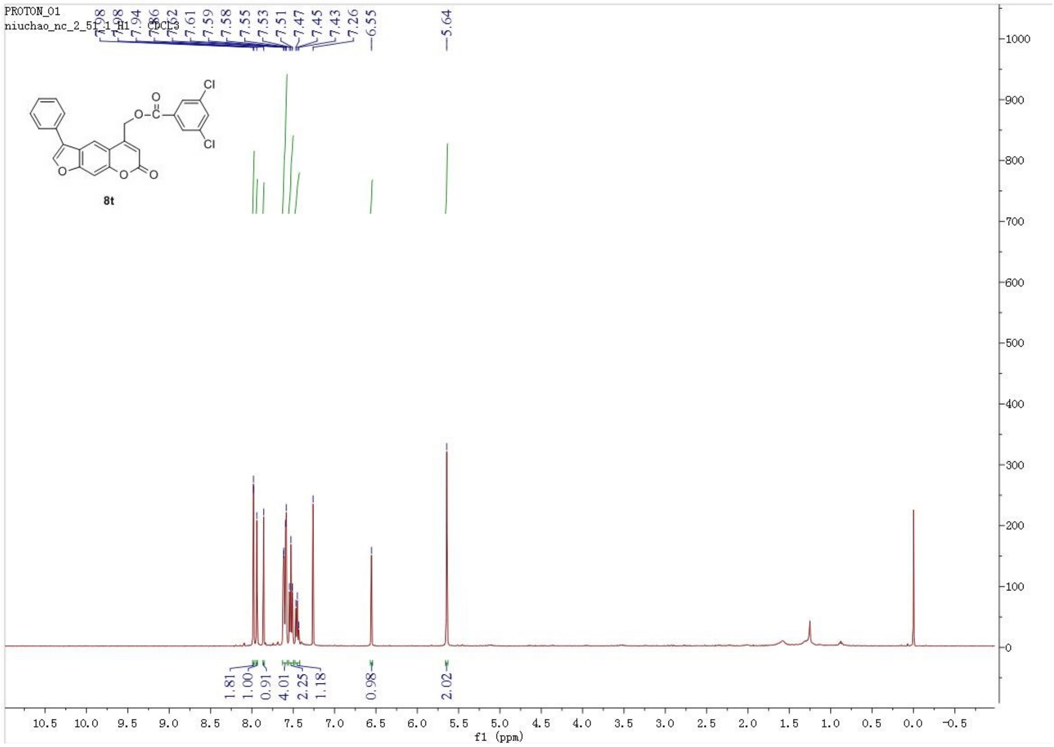

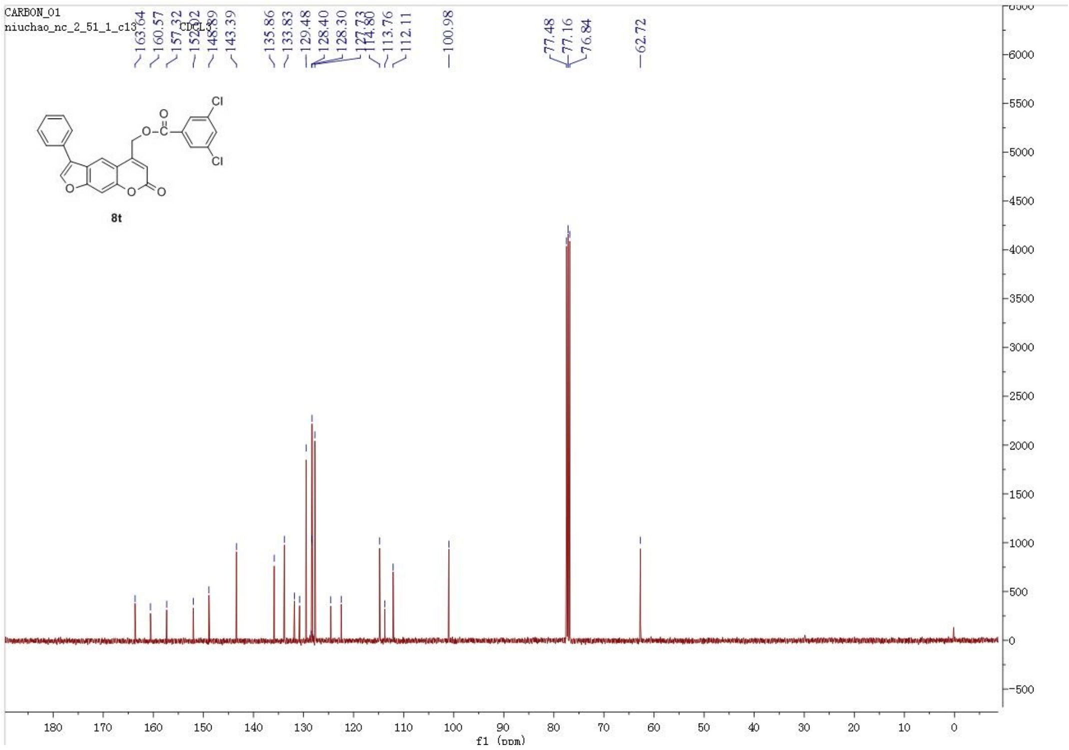

^{1}H NMR and ^{13}C NMR spectra of **8u**

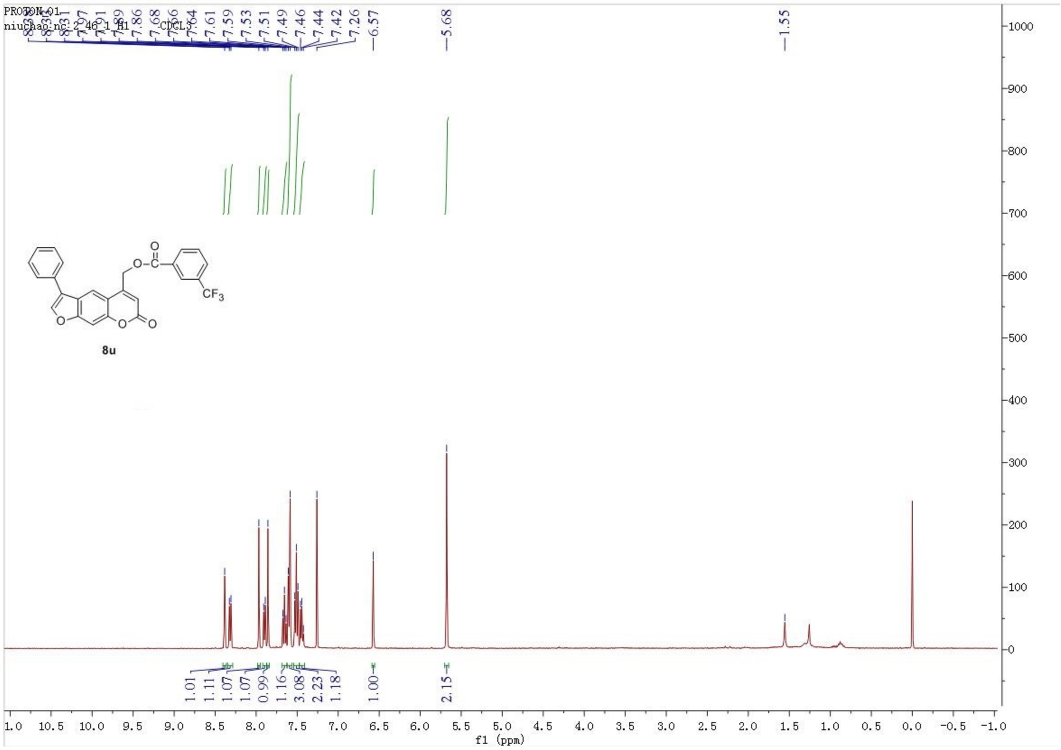

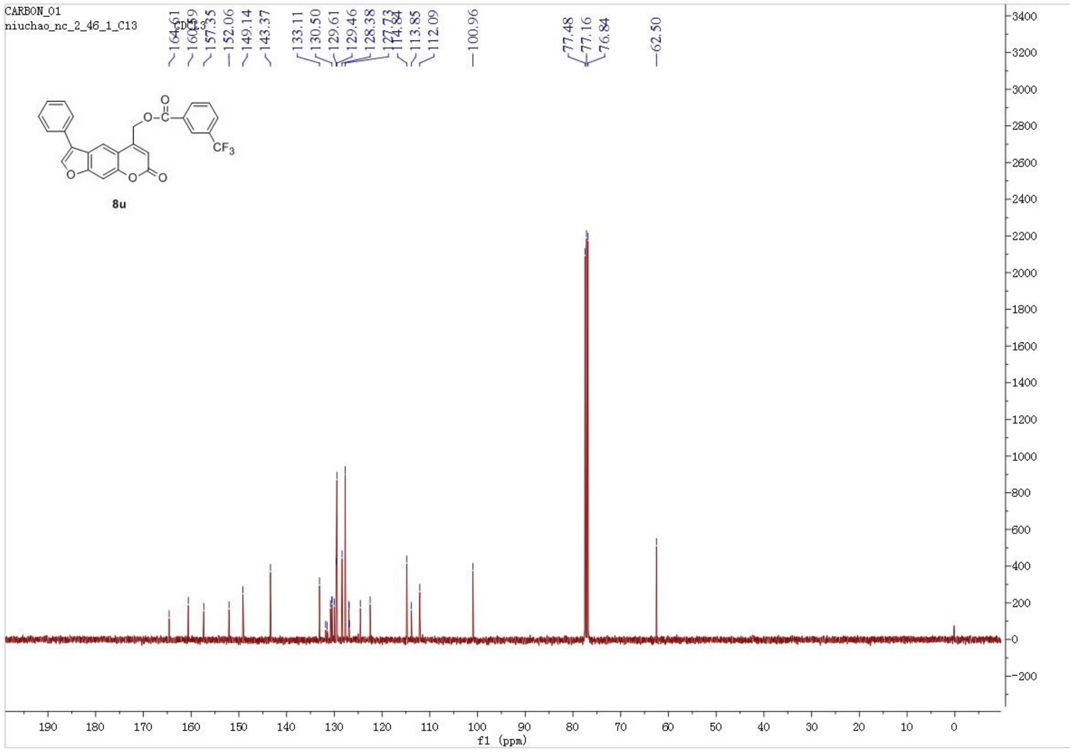

^{1}H NMR and ^{13}C NMR spectra of **8v**

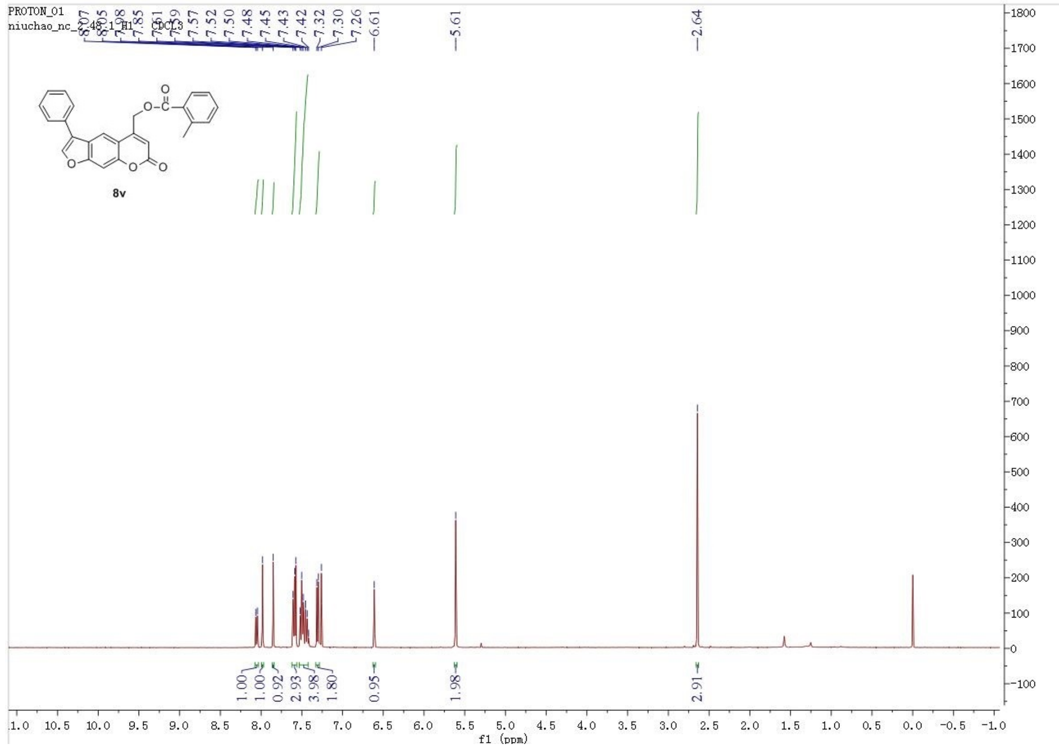

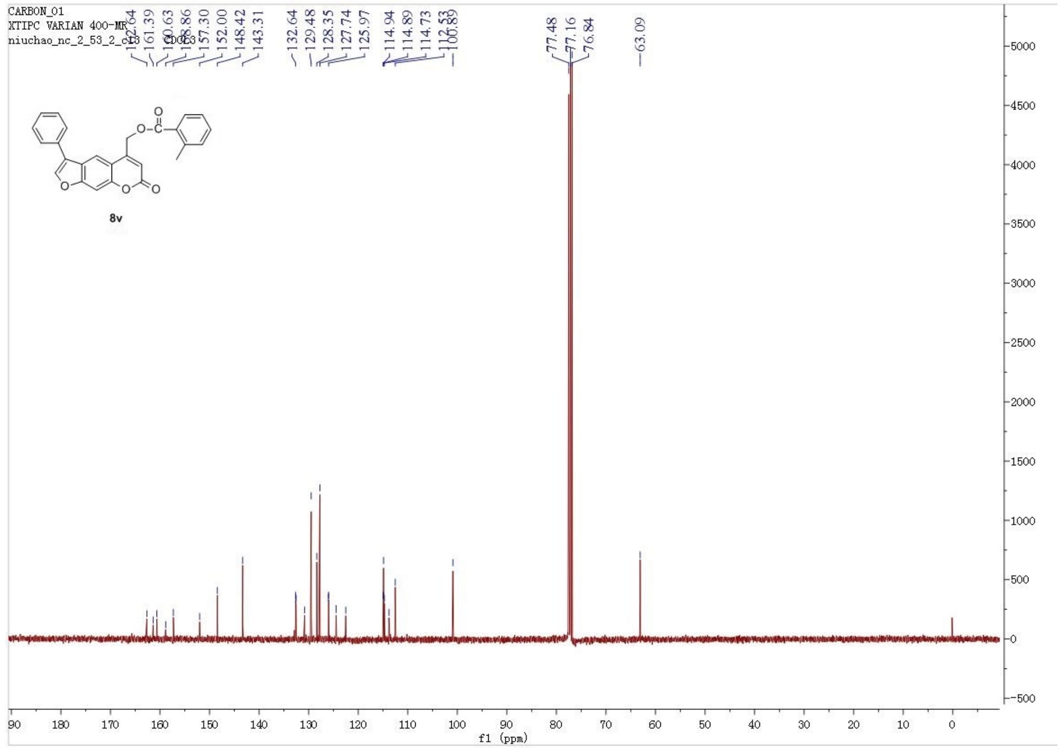

^{1}H NMR and ^{13}C NMR spectra of **8w**

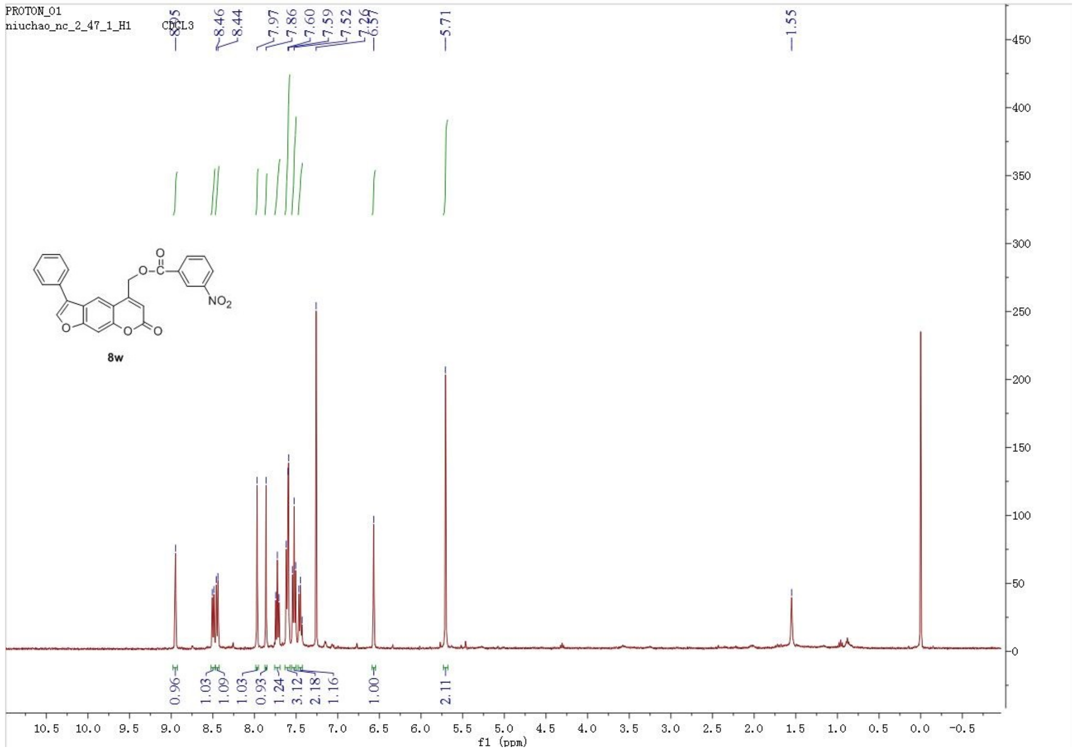

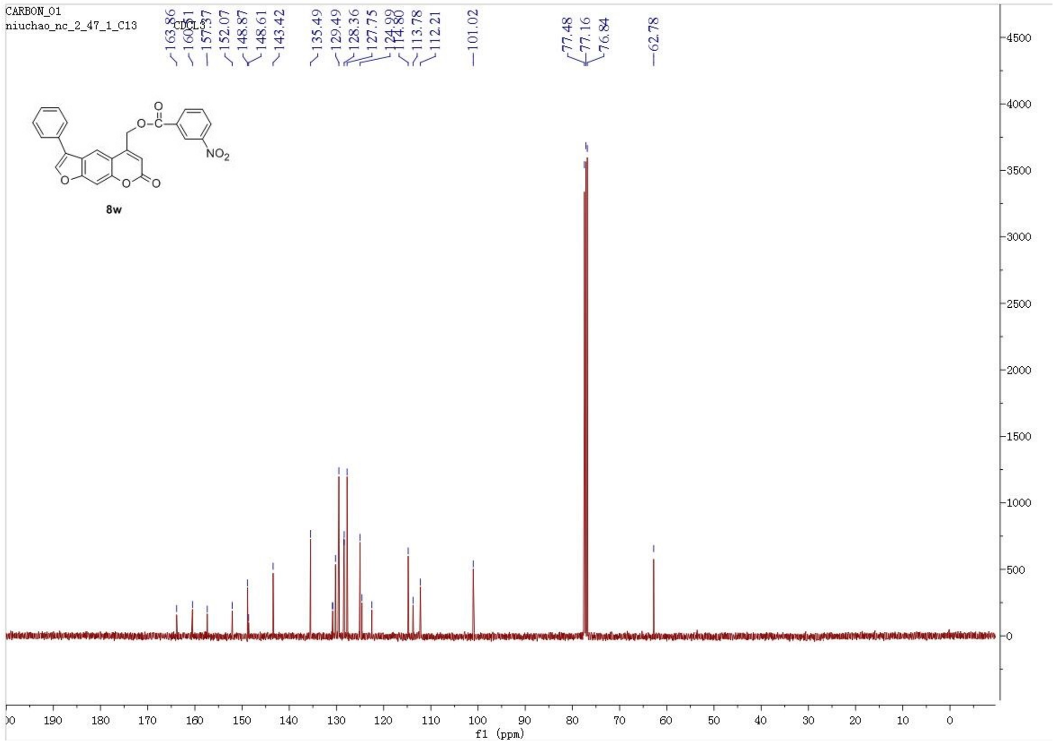

^{1}H NMR and ^{13}C NMR spectra of **8x**

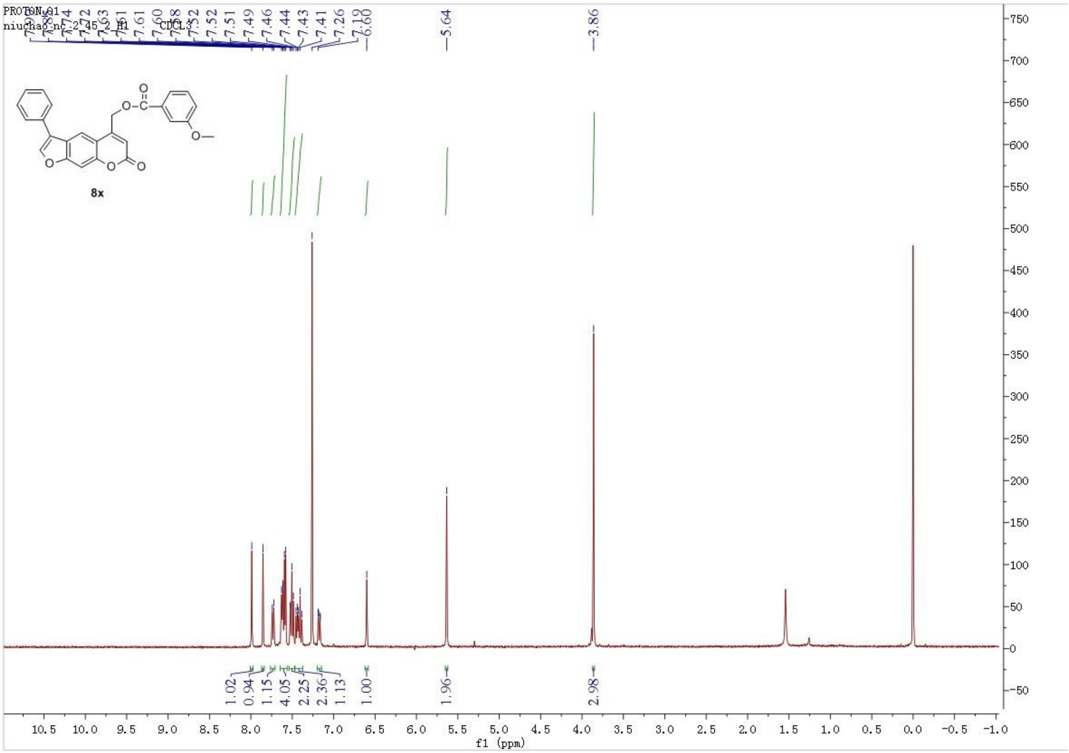

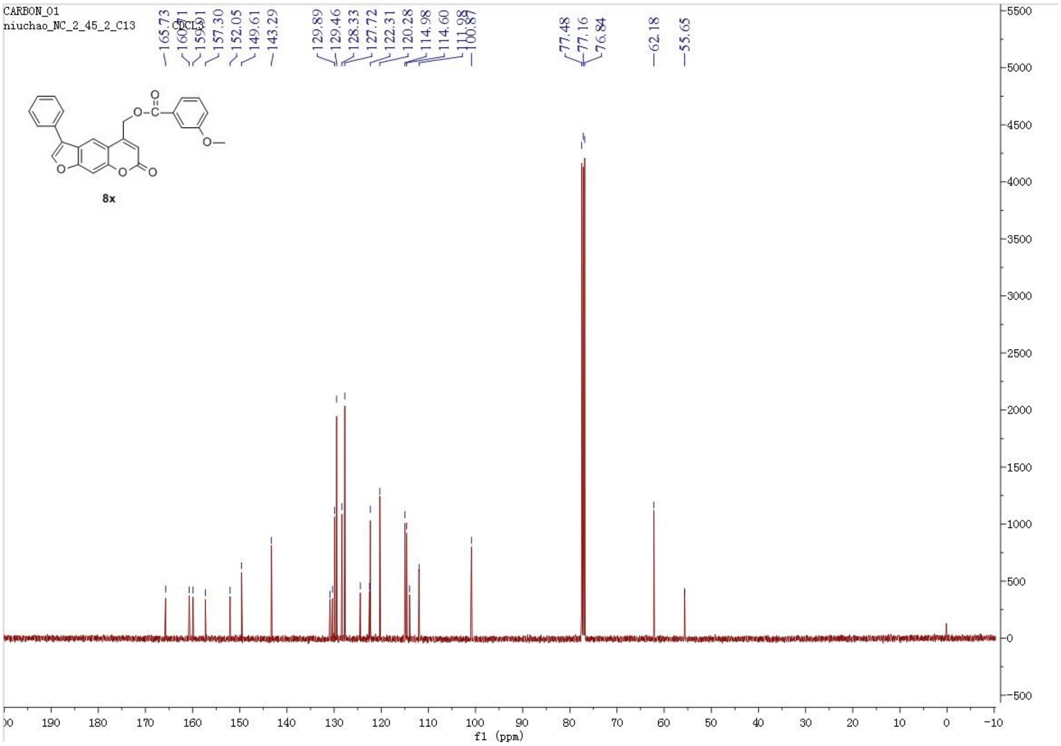

^{1}H NMR and ^{13}C NMR spectra of **8y**

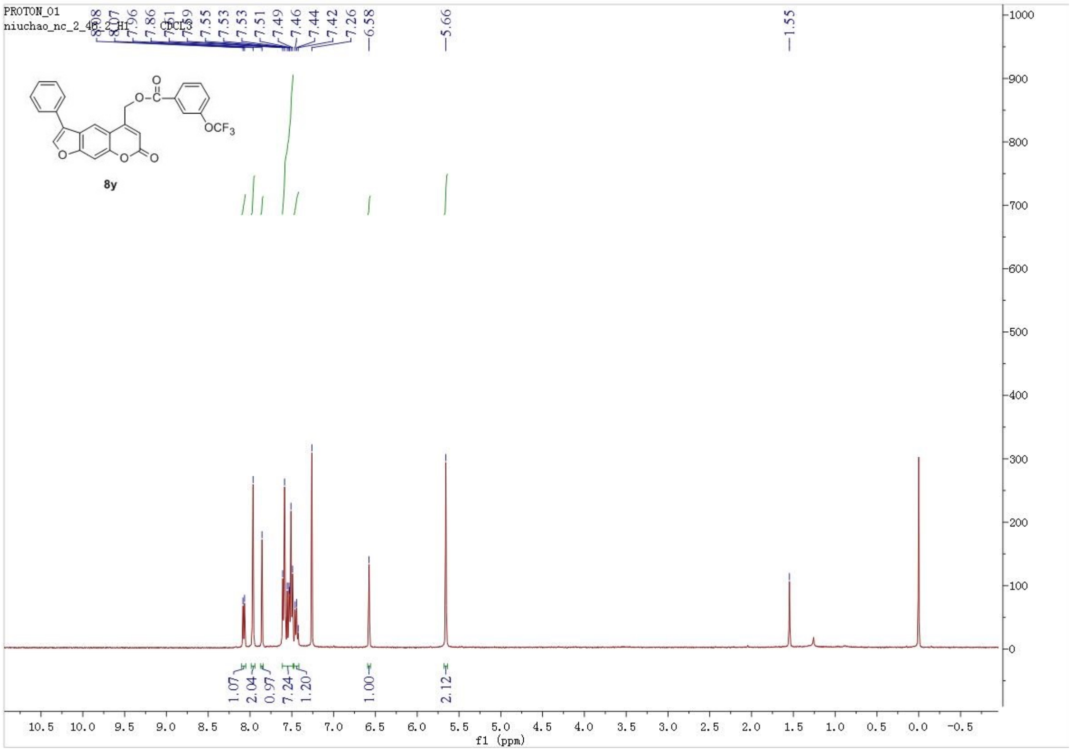

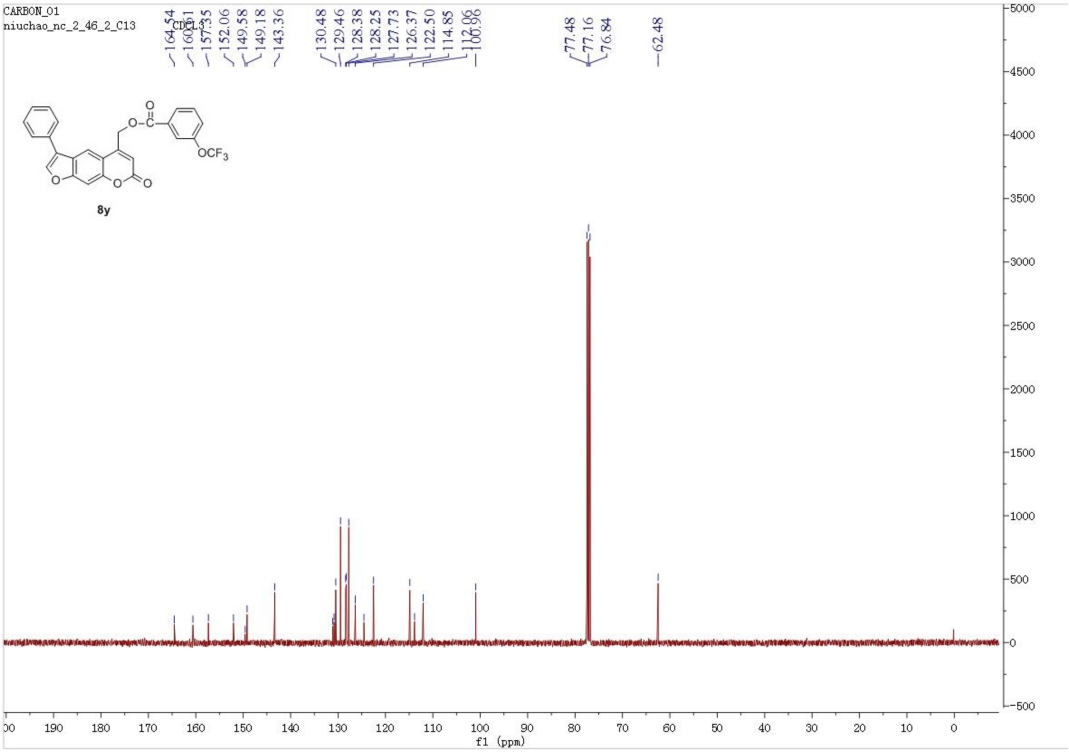

^{1}H NMR and ^{13}C NMR spectra of **8z**

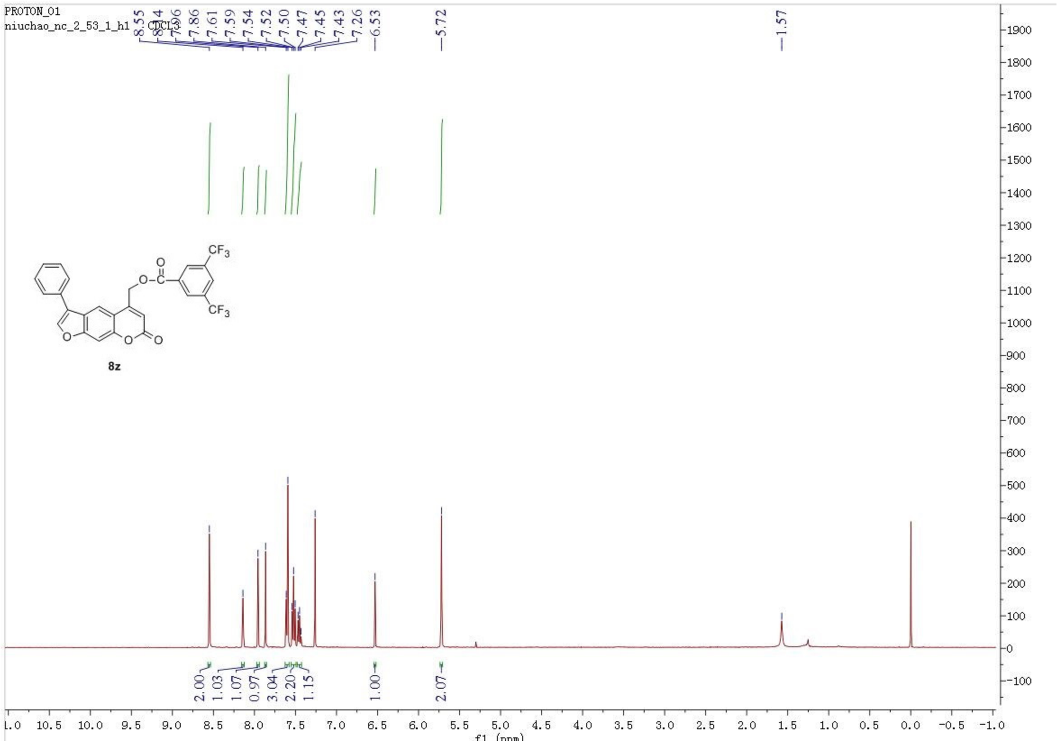

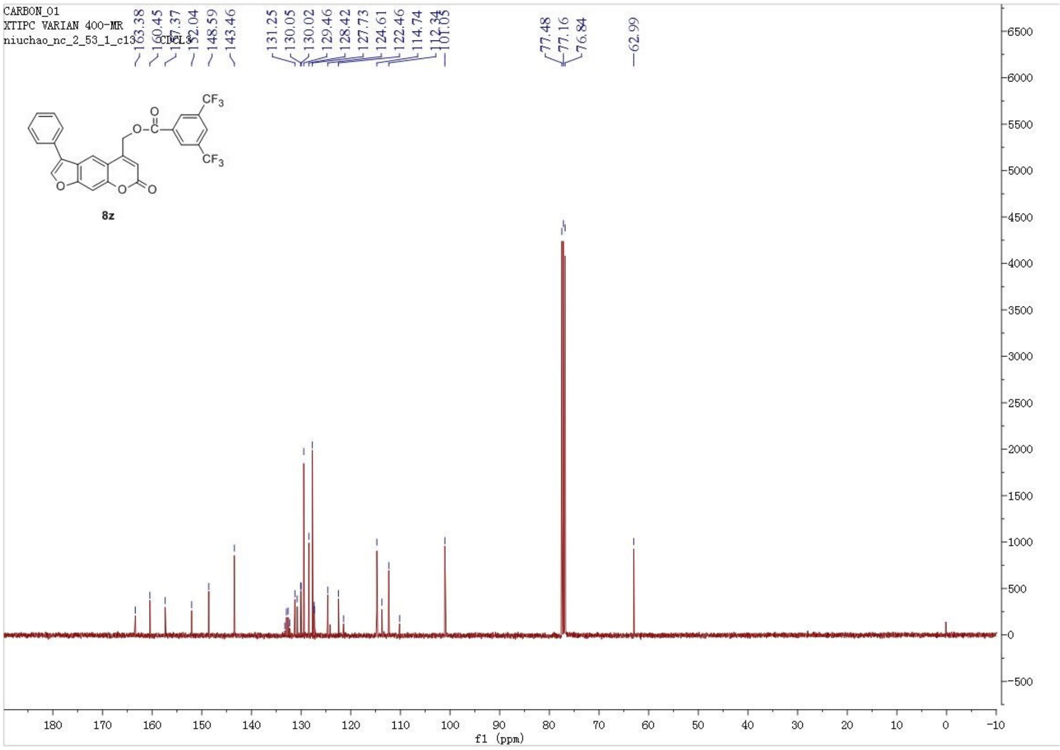

^{1}H NMR and ^{13}C NMR spectra of **8aa**

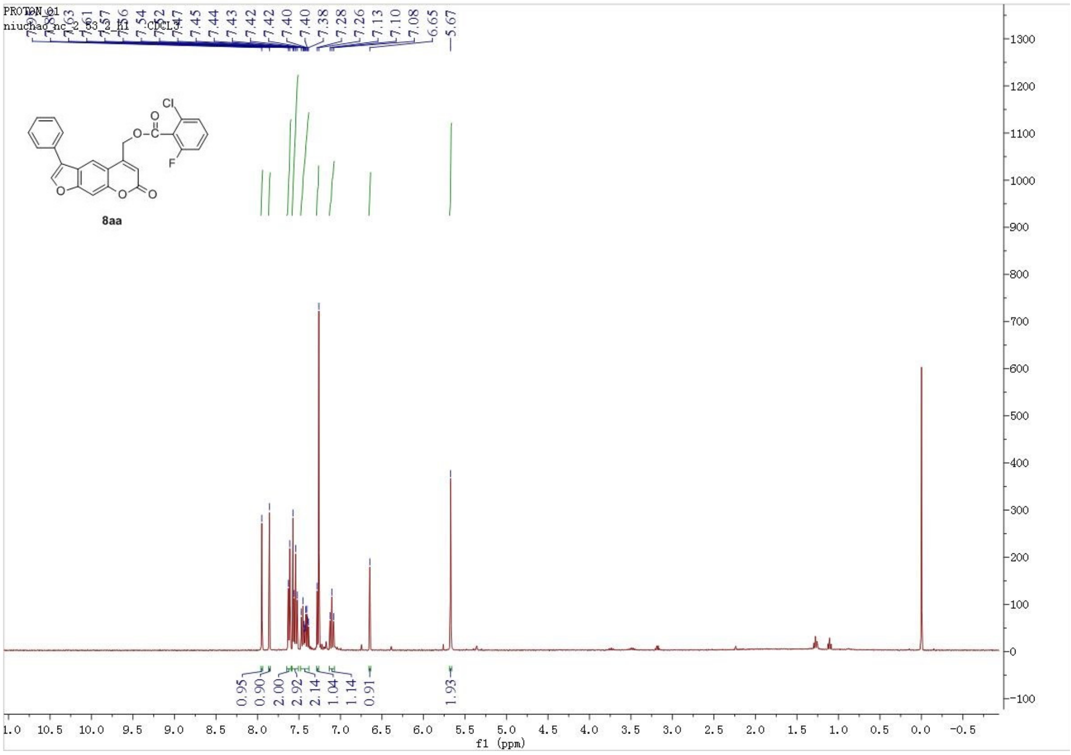

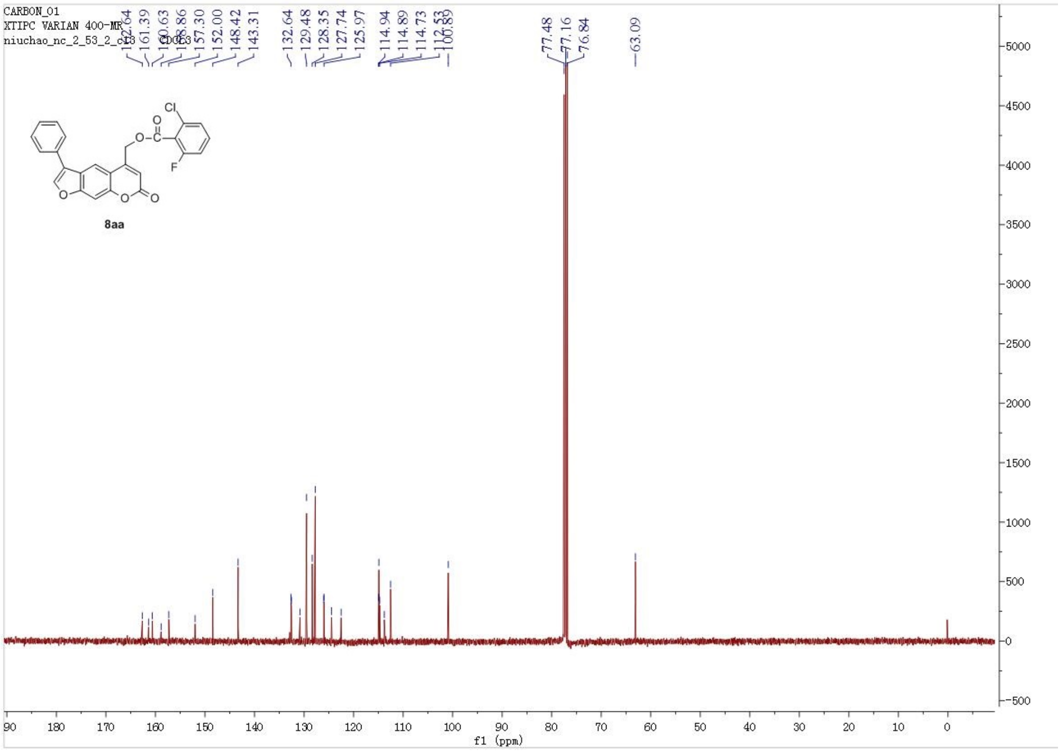

^{1}H NMR and ^{13}C NMR spectra of **8ab**

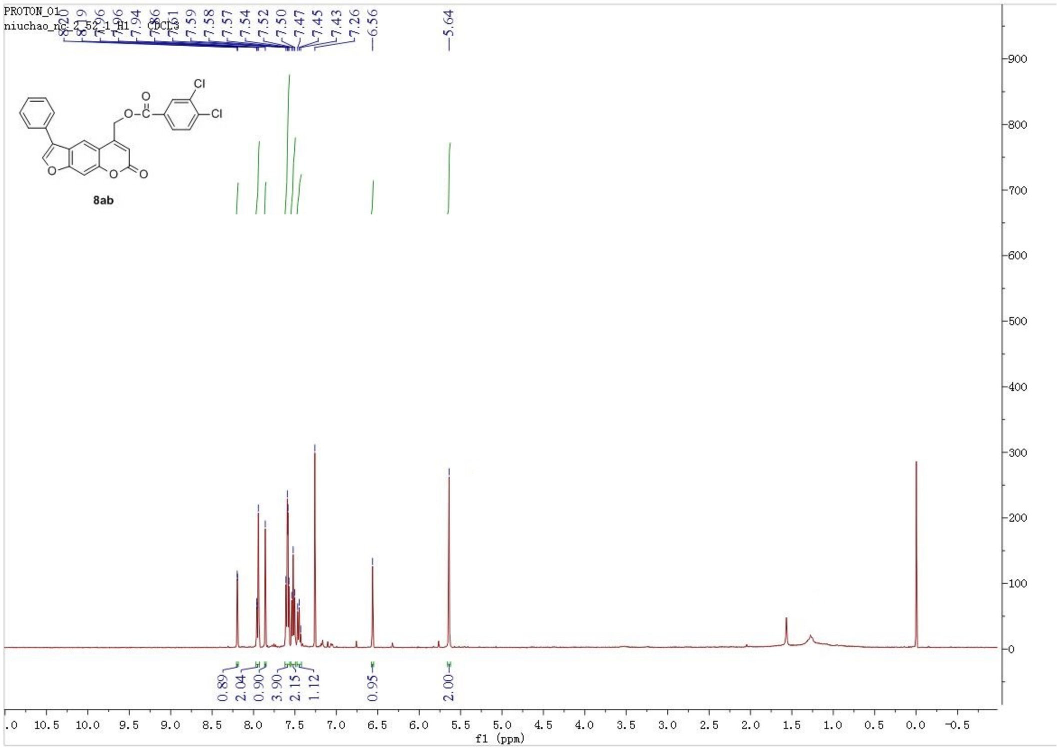

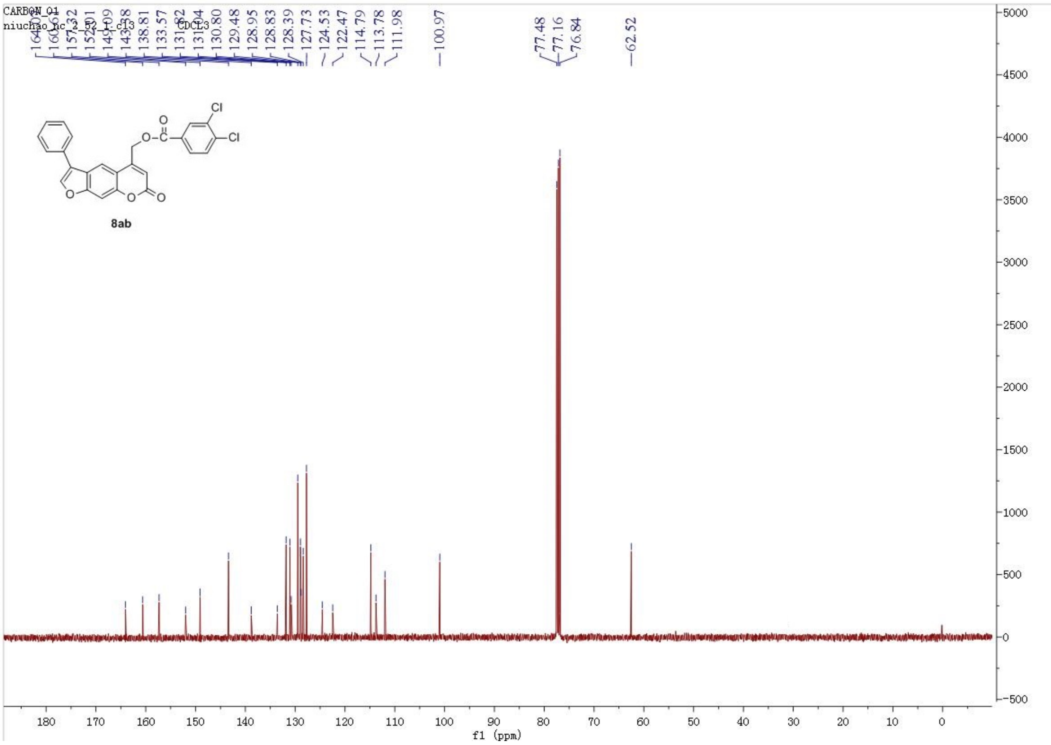

^{1}H NMR and ^{13}C NMR spectra of **8ac**

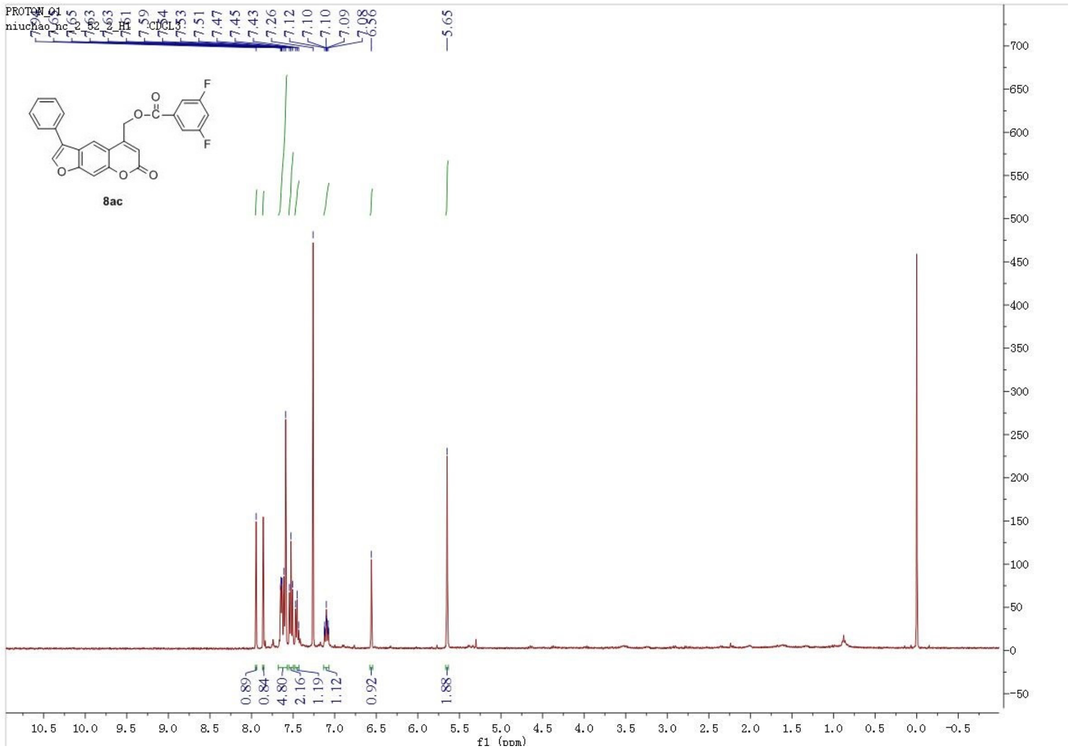

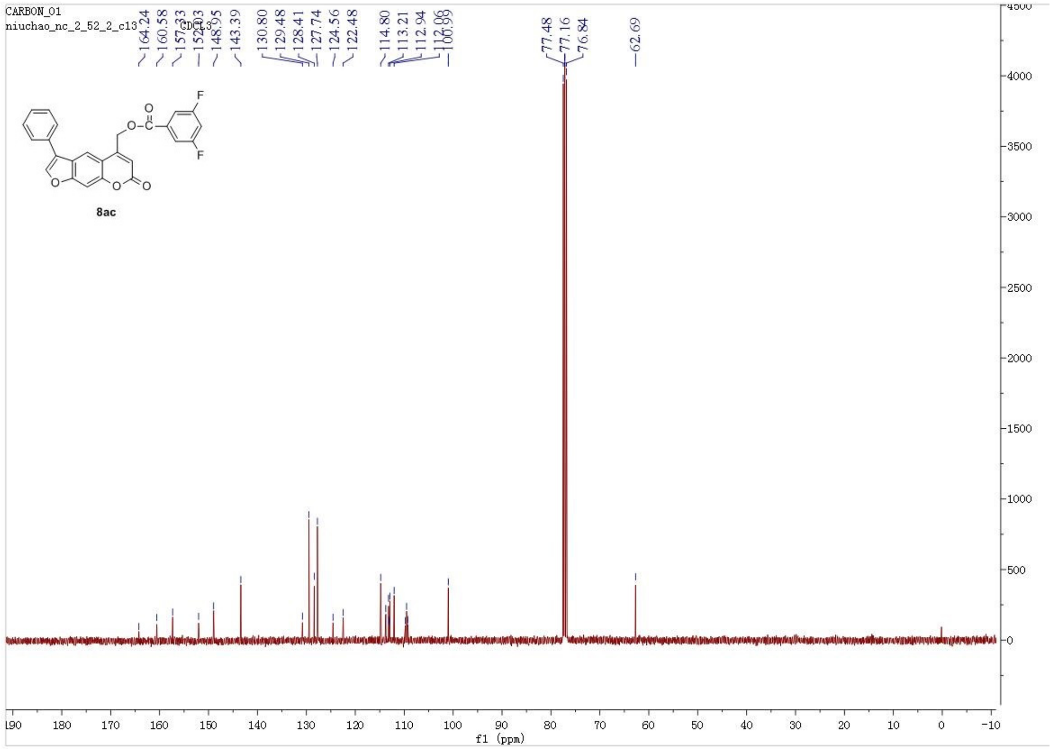

^{1}H NMR and ^{13}C NMR spectra of **8ad**

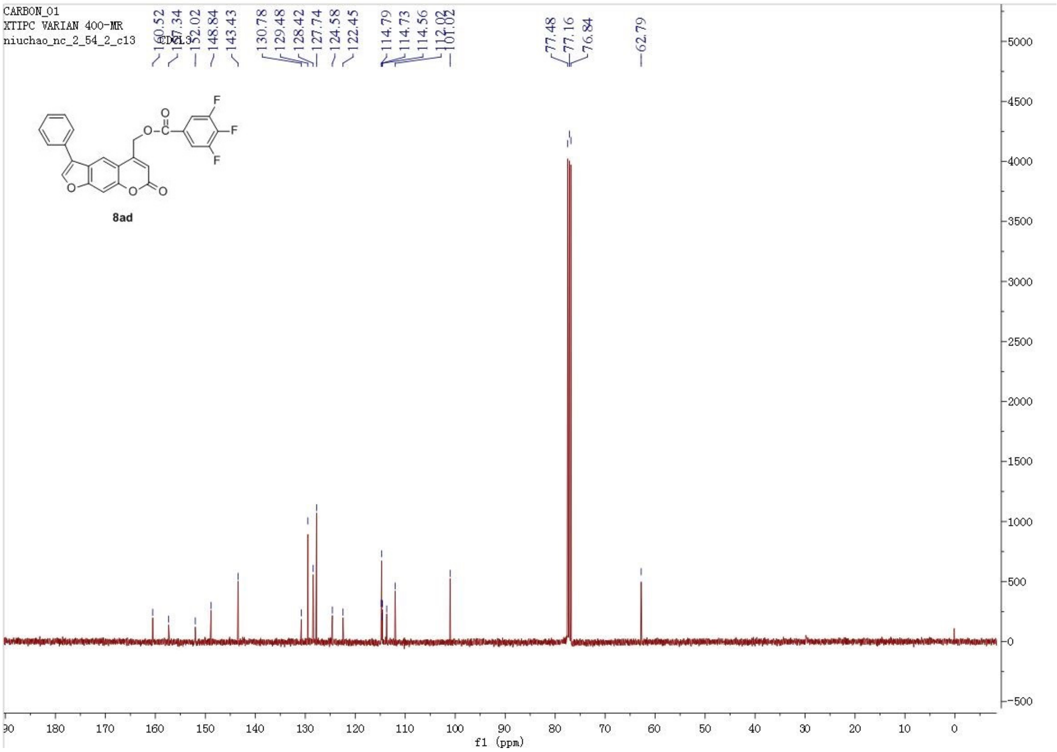

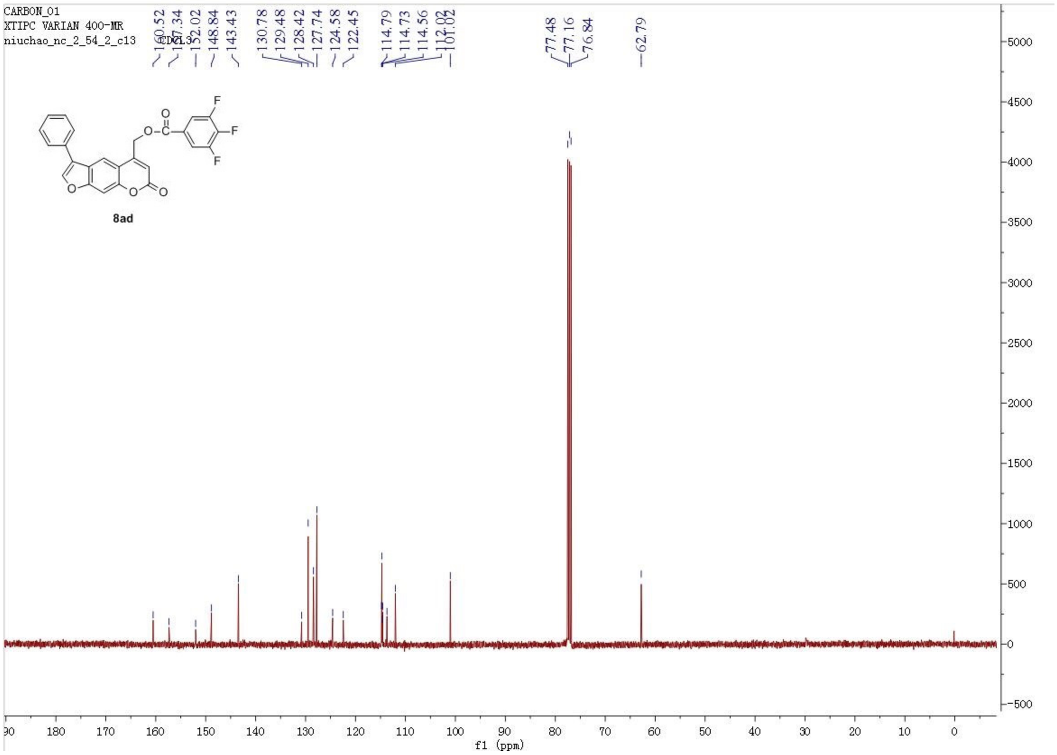

^1H NMR and ^{13}C NMR spectra of **8ad**

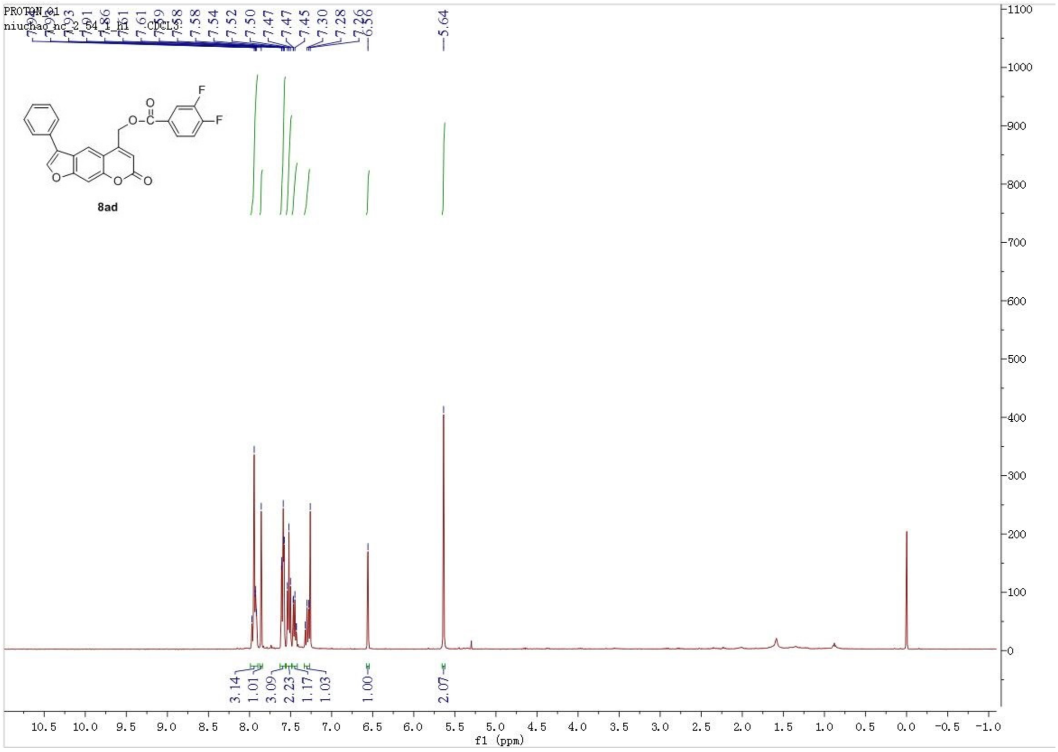

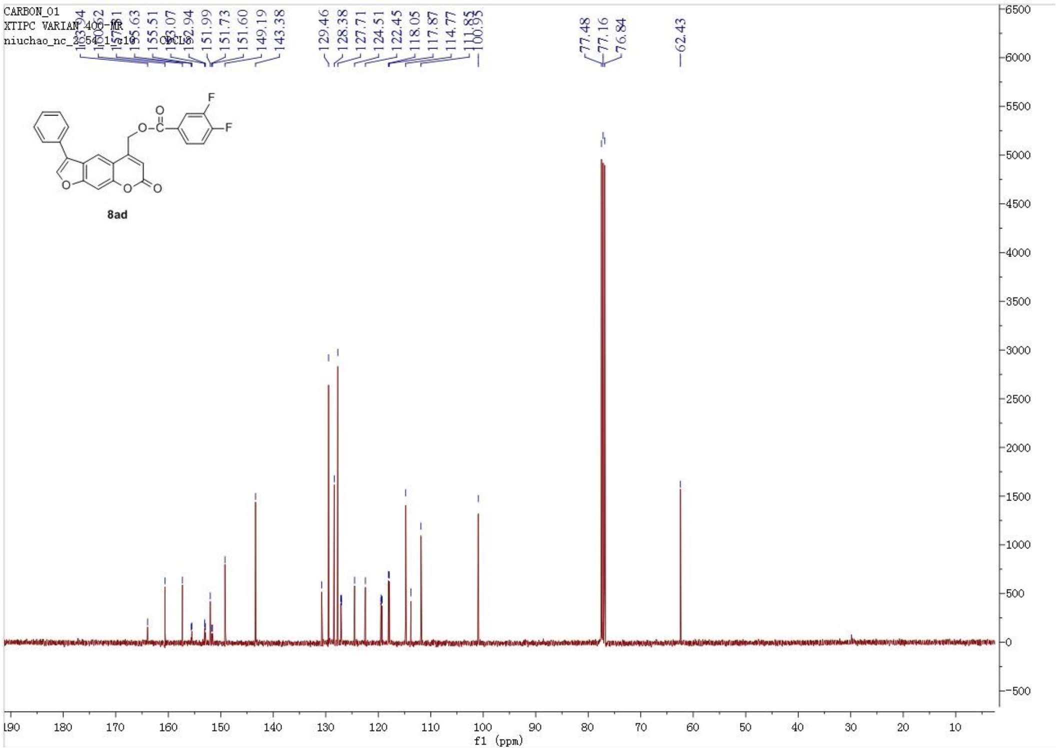

^{1}H NMR and ^{13}C NMR spectra of **8af**

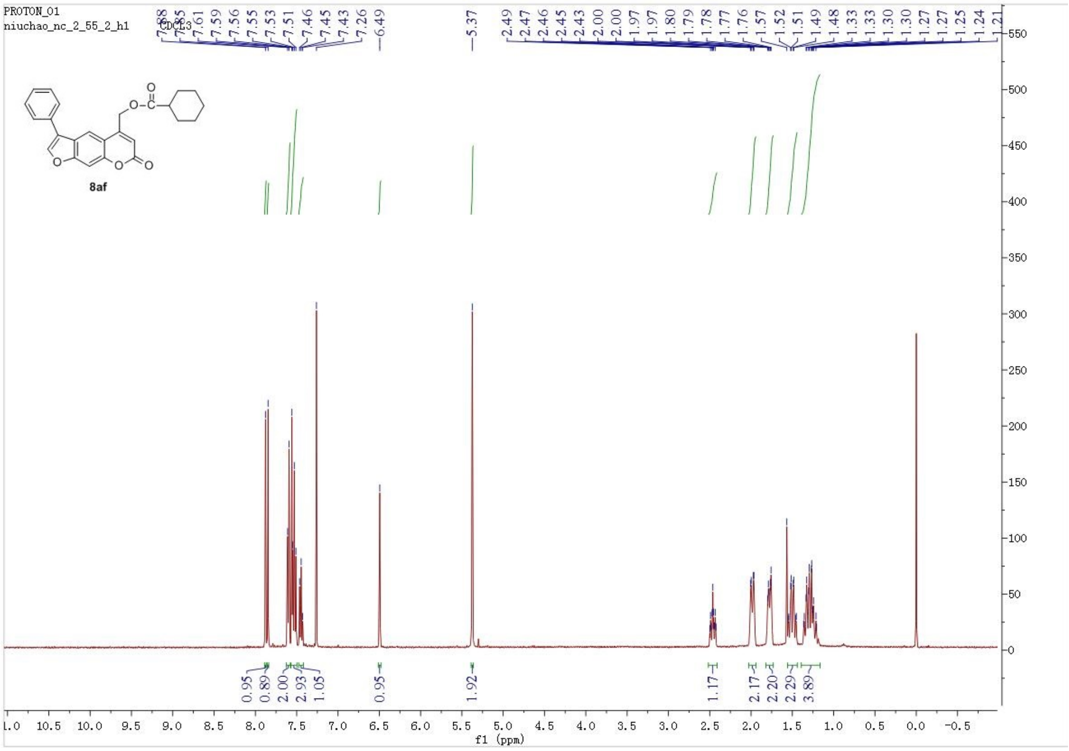

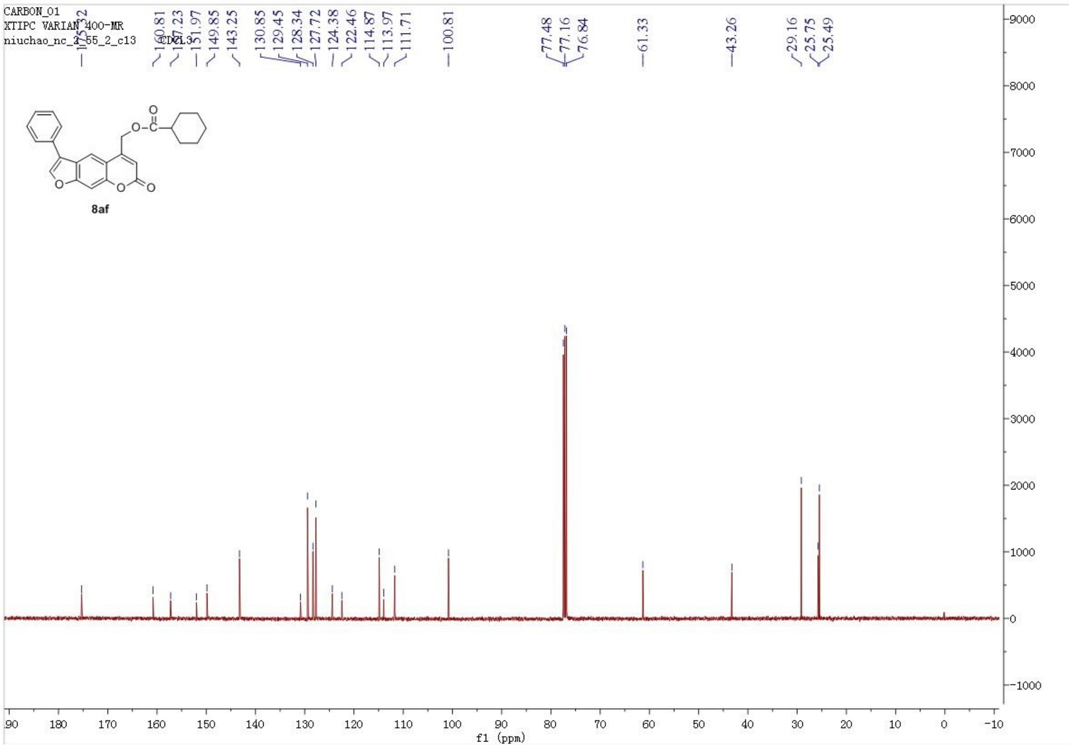

^{1}H NMR and ^{13}C NMR spectra of **8ag**

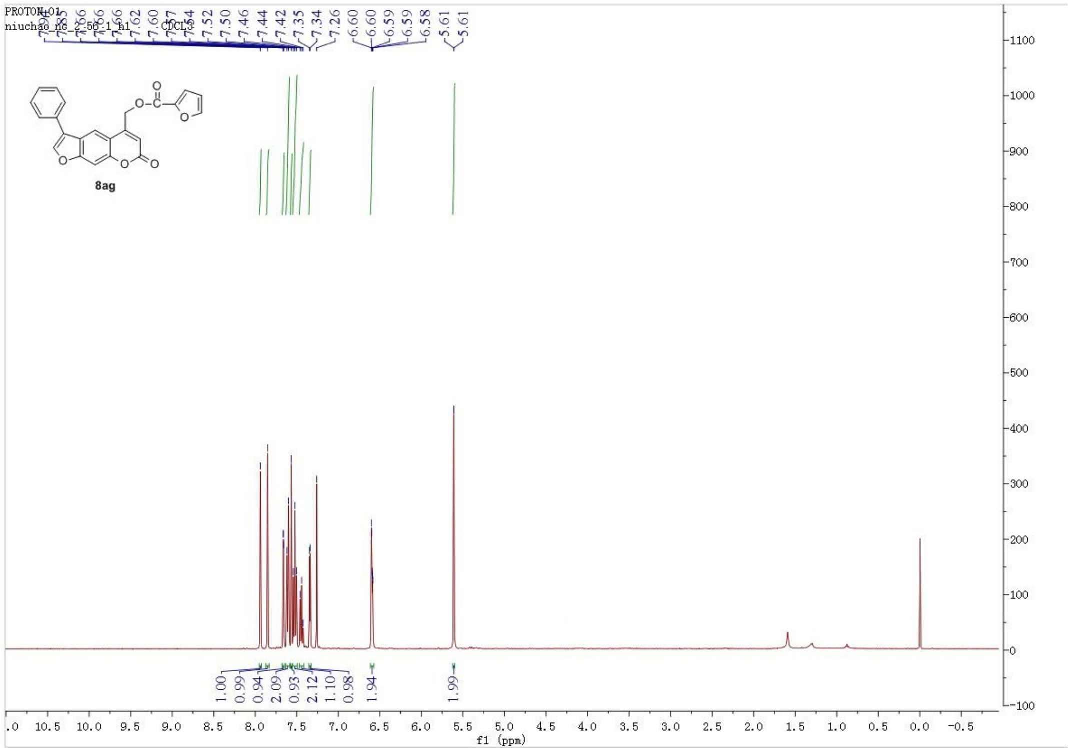

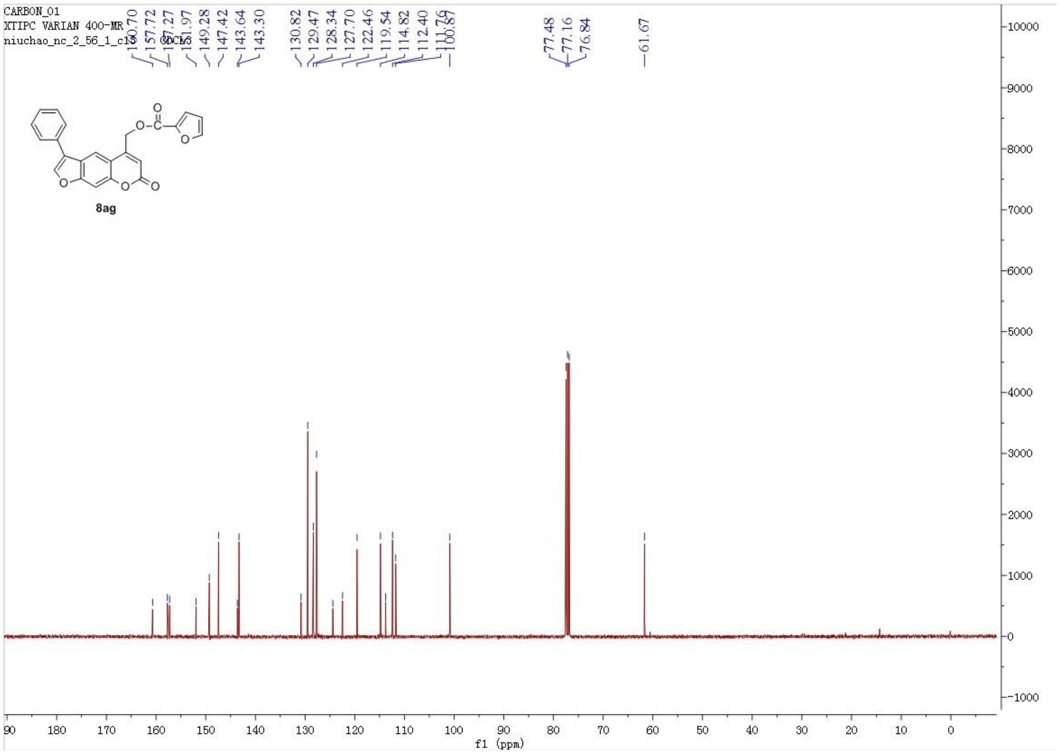

Supplement: Supplementary file 1 [file ijms-23-07959-s001.zip › ijms-1781859-supplementary.pdf]
